# MicroRNA-325-3p Facilitates Immune Escape of Mycobacterium tuberculosis through Targeting LNX1 via NEK6 Accumulation to Promote Anti-Apoptotic STAT3 Signaling

**DOI:** 10.1128/mBio.00557-20

**Published:** 2020-06-02

**Authors:** Beibei Fu, Weiwei Xue, Haiwei Zhang, Rui Zhang, Kelly Feldman, Qingting Zhao, Shanfu Zhang, Lei Shi, Krishna Chaitanya Pavani, Weiqi Nian, Xiaoyuan Lin, Haibo Wu

**Affiliations:** a School of Life Sciences, Chongqing University, Chongqing, China; b School of Pharmaceutical Sciences, Chongqing University, Chongqing, China; c Chongqing Key Laboratory of Translational Research for Cancer Metastasis and Individualized Treatment, Chongqing University Cancer Hospital, Chongqing, China; d Department of Respiratory Medicine, The First Affiliated Hospital of Chongqing Medical University, Chongqing, China; e Department of Molecular and Cell Biology, University of California, Berkeley, California, USA; f Department of Nutrition, Genetics and Ethology, Ghent University, Merelbeke, Belgium; Washington University School of Medicine in St. Louis

**Keywords:** *Mycobacterium tuberculosis*, microRNA, macrophage, ubiquitination, immune escape

## Abstract

Tuberculosis (TB) is an infectious disease caused by Mycobacterium tuberculosis that poses threats to the public. M. tuberculosis survives in macrophages by escaping from immune surveillance and clearance, which exacerbates the bacterial proliferation. However, the molecular mechanisms of this immune escape have not yet been fully understood. Using multiple cell and mouse models, we found that microRNA-325-3p (miR-325-3p) is upregulated after M. tuberculosis infection and *Mir325*-deficient mice show resistance to M. tuberculosis. We demonstrated that miR-325-3p directly targets LNX1, an E3 ubiquitin ligase of NEK6, and that this hampers the proteasomal degradation of NEK6 in macrophages. The abnormal accumulation of NEK6 leads to the activation of STAT3 signaling, thus inhibiting the process of apoptosis and promoting the intracellular survival of M. tuberculosis. Our findings not only reveal a new immune escape pathway of M. tuberculosis but also may provide new insights into the development of therapeutic approaches for drug-resistant TB.

## Abstract (precis)

Intracellular survival of Mycobacterium tuberculosis results in bacterial proliferation and the spread of infection in lungs, consequently deteriorating the conditions of tuberculosis (TB) patients. This research discovers a new immune escape pathway of M. tuberculosis by modulating host miR-325-3p expression, thus leading to the intracellular survival of M. tuberculosis. These findings make a contribution to the understanding of the immune escape of M. tuberculosis, and they provide a theoretical basis for the development of therapeutic approaches for drug-resistant TB.

## INTRODUCTION

Currently, tuberculosis (TB) continues to be the most important cause of death from a single infectious microorganism. Worldwide, up to 10 million people fell ill with TB in 2018 (1). As the causative agent of TB, Mycobacterium tuberculosis typically invades the organism through respiratory tract and causes lesions in lung (2). Mostly, the host immune system recognizes and clears the invasive M. tuberculosis to prevent a further spread of the infection (3, 4). On the other hand, M. tuberculosis can evolve and adopt strategies to escape from the surveillance of the host immune system (4, 5).

MicroRNAs (miRNAs) are single-stranded RNAs (approximately 22 nucleotides in length) that transcriptionally/posttranscriptionally regulate gene expression (6). miRNA-mRNA networks are involved in many immune signaling pathways (7), especially in the immune escape of M. tuberculosis: microRNA let-7 targets A20 to help M. tuberculosis suppress innate immune responses in macrophages (8); microRNA-27a targets endoplasmic reticulum (ER)-located Ca^2+^ transporter CACNA2D3 to inhibit autophagosome formation and to promote the intracellular survival of M. tuberculosis (9). In addition, the results from microRNA sequencing (miRNA-seq) showed that a large amount of miRNAs were up- or downregulated during M. tuberculosis infection (10). These findings suggested that miRNAs are likely to influence the outcome of the encounter between macrophages and M. tuberculosis.

Ubiquitination is a critical posttranslational modification in several cellular responses (11). The ubiquitin system is composed of activating enzyme (E1), conjugating enzyme (E2), and ligase (E3). The high efficiency and exquisite selectivity of ubiquitination reactions are attributable to the function of E3. To be more specific, E3 recognizes substrates based on the presence of a specific ubiquitination signal and catalyzes the formation of an isopeptide bond between a substrate lysine residue and the C terminus of ubiquitin. Ubiquitin-tagged substrates are recognized by proteasomes, which can induce protein degradation (12). The ubiquitin-mediated degradation of pivotal proteins is essential to the precise control of the immune system (13). For example, as a potent inhibitor of interferon-dependent antiviral responses, E3 ubiquitin ligase RNF2 directly binds to STAT1 after interferon stimulation and increases K33-linked polyubiquitination of STAT1 at position K379. In this way, RNF2 promotes the disassociation of STAT1/STAT2 from DNA and consequently suppresses transcription of interferon-stimulated genes (ISG) (14). The immune escape pathway of M. tuberculosis associated with ubiquitination has also been reported. For example, PtpA, a secreted tyrosine phosphatase essential for tuberculosis pathogenicity, can suppress innate immunity by competitively binding to ubiquitin-interacting domain of the host adaptor TAB3 (15). These findings revealed a close link between ubiquitination and the host immune system.

In previous studies, we found that microRNA-325-3p (miR-325-3p) was upregulated in a susceptible mouse substrain after aerosol infection with M. tuberculosis. Also, relatively higher levels of miR-325-3p were found in latent TB patients than in healthy controls (data not published), indicating an important role for miR-325-3p in M. tuberculosis intracellular survival. In this study, we demonstrated for the first time that *Mir325*-deficient mice were resistant to M. tuberculosis infection. More importantly, we found that the ligand of numb-protein X 1 (LNX1), a direct target of miR-325-3p, is an E3 ubiquitin ligase of NIMA-related expressed kinase 6 (NEK6). During M. tuberculosis infection, LNX1 reduction promotes the abnormal accumulation of NEK6, resulting in an inhibition of apoptosis and promotion of the intracellular survival of M. tuberculosis. All these results not only made an important contribution to the understanding of the immune escape pathways of M. tuberculosis but also provided a theoretical basis for the development of therapeutic approaches for drug-resistant TB.

## RESULTS

### miR-325-3p is associated with M. tuberculosis survival in macrophages.

**FIG 1:**
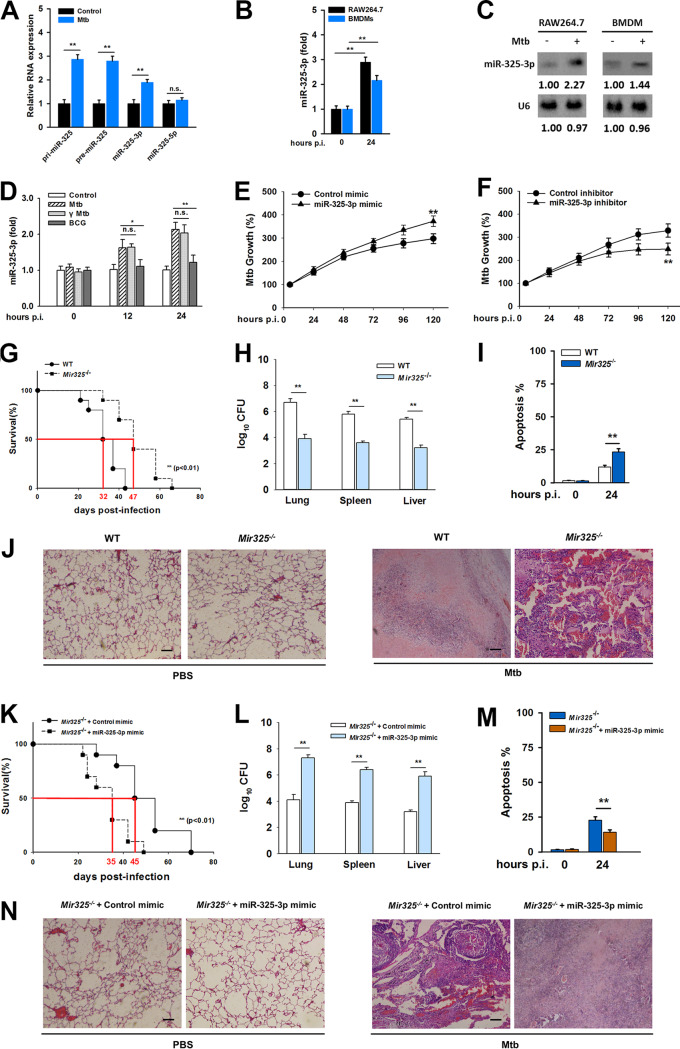
miR-325-3p associates with M. tuberculosis survival in macrophages. (A) The expression changes of pri-miR-325, pre-miR-325, and mature miR-325 were analyzed by qRT-PCR in RAW 264.7 cells infected with M. tuberculosis for 24 h (MOI = 5). (B) Expression levels of miR-325-3p in RAW 264.7 cells and BMDMs after M. tuberculosis infection (MOI = 5). (C) The expression changes of miR-325-3p transcripts were analyzed by Northern blotting in RAW 264.7 cells and BMDMs infected with M. tuberculosis for 24 h (MOI = 5). (D) Expression levels of miR-325-3p in RAW 264.7 cells during infection with M. tuberculosis (MOI = 5), 10 μg/ml of gamma-irradiated M. tuberculosis, or BCG. (E and F) M. tuberculosis growth rates in RAW 264.7 cells transfected with miR-325-3p mimic (E) or miR-325-3p inhibitor (F) (MOI = 5). (G) Survival of WT (*n* = 10) and *Mir325*^−/−^ mice (*n* = 10) after aerosol infection with approximately 400 CFU of M. tuberculosis. (H) M. tuberculosis bacterial loads in lungs, spleens, and livers from the WT and *Mir325*^−/−^ mice at 21 dpi. (I) Apoptosis rates of BMDMs from WT and *Mir325*^−/−^ mice stimulated with M. tuberculosis for 24 h (MOI = 5). (J) Typical lung lesions of the WT and *Mir325*^−/−^ mice at 21 dpi. Scale bar = 100 μm. (K) Survival of *Mir325*^−/−^ mice transfected with control mimic (*n* = 10) or miR-325-3p mimic (*n* = 10) *in vivo*. (L) M. tuberculosis bacterial loads in lungs, spleens, and livers from *Mir325*^−/−^ mice transfected with miR-325-3p mimic *in vivo*. (M) Apoptosis rates of BMDMs from *Mir325*^−/−^ mice transfected with miR-325-3p mimic *in vivo*. BMDMs were stimulated with M. tuberculosis for 24 h (MOI = 5). (N) Typical lung lesions of *Mir325*^−/−^ mice transfected with miR-325-3p mimic *in vivo*. Scale bar = 100 μm. Mouse survival data were plotted as Kaplan-Meier curves and compared by log rank (Mantel-Cox) test. Bacterial loads were analyzed using the Mann-Whitney U test. For other data, statistical significance between groups was determined by two-tailed Student’s *t* test or one-way ANOVA followed by Bonferroni *post hoc* test. All data are presented as the mean ± SDs and were derived from three independent experiments. *, *P* < 0.05; **, *P* < 0.01. n.s., not significant.

To determine the role of miR-325 during M. tuberculosis infection, expression levels of primary (pri-), precursor (pre-), and mature miR-325 transcripts were analyzed in RAW 264.7 cells infected with M. tuberculosis. M. tuberculosis infection increased the expression levels of pri-miR-325, pre-miR-325, and miR-325-3p instead of miR-325-5p (Fig. 1A). The upregulation of miR-325-3p was also observed in mouse bone marrow-derived macrophages (BMDMs) at 24 h postinfection (Fig. 1B). Furthermore, the results from Northern blotting showed elevated miR-325-3p in macrophages after M. tuberculosis infection (Fig. 1C). Since gamma-irradiated M. tuberculosis had an ability to upregulate miR-325-3p similar to that of live M. tuberculosis (Fig. 1D), we used gamma-irradiated M. tuberculosis instead of live M. tuberculosis in some of the following studies. Mycobacterium bovis BCG, which lacks the RD1 genomic region, was unable to upregulate miR-325-3p (Fig. 1D). RD1 encodes two major antigens, ESAT6 and CFP10, which are associated with the virulence of M. tuberculosis strains (16). The loss of ESAT6 or CFP10 affected the upward adjustment of M. tuberculosis to miR-325-3p (see Fig. S1A in the supplemental material). In order to examine whether miR-325-3p plays a role in M. tuberculosis survival, RAW 264.7 cells were transfected with an miR-325-3p mimic or inhibitor (Fig. S1B). After infection with M. tuberculosis, bacterial CFU were scored every 24 h and converted to M. tuberculosis growth rates. The results showed that M. tuberculosis survival was augmented in the presence of the miR-325-3p mimic (Fig. 1E). Conversely, M. tuberculosis survival was compromised in macrophages treated with the miR-325-3p inhibitor (Fig. 1F). Furthermore, the function of miR-325-3p in M. tuberculosis pathogenesis was studied using wild-type (WT) and *Mir325*-deficient (*Mir325*^−/−^) mice. The median survival time of *Mir325*^−/−^ mice aerosol infected with M. tuberculosis, compared to that of the WT mice, was significantly lengthened (Fig. 1G). At 21 days postinfection (dpi), the lungs, spleens, and livers of *Mir325*^−/−^ mice infected with M. tuberculosis had lower bacterial loads than those of the WT mice (Fig. 1H). After M. tuberculosis stimulation, the macrophages from *Mir325*^−/−^ mice showed a higher apoptosis rate (Fig. 1I). Pathology analysis at 21 dpi showed that increased necrotic lesions in lungs of WT mice, while only a trail of inflammation was left in those of *Mir325*^−/−^ mice (Fig. 1J and Table S1). This phenomenon indicated that *Mir325*^−/−^ mice were more resistant to M. tuberculosis infection than WT mice. Finally, an *in vivo* transfection method was used to confirm the functional effect of miR-325-3p. The miR-325-3p mimic or control mimic was transfected into *Mir325*^−/−^ mice through the tail intravenously (Fig. S1C), and the survival rates, organ bacterial burdens, and macrophage death mechanisms were determined afterwards to identify pathological damage. The results showed that *Mir325*^−/−^ mice transfected with the miR-325-3p mimic had restored susceptibility to M. tuberculosis (Fig. 1K to M). Likewise, pathology analysis of lungs supported increased necrotic lung lesions in mice with a higher miR-325-3p level (Fig. 1N and Table S1). Taken together, these data suggested that miR-325-3p is upregulated after M. tuberculosis infection and is unfavorable for host resistance to M. tuberculosis.

10.1128/mBio.00557-20.1 FIG S1 miR-325-3p is upregulated during M. tuberculosis infection. (A) RAW 264.7 cells were infected with two M. tuberculosis mutant strains (loss of ESAT6 or CFP10), and the expressions of miR-325-3p were detected by qRT-PCR (MOI = 5). (B) Expression levels of miR-325-3p in macrophages in the presence of miR-325-3p mimic or inhibitor. (C) Expression levels of miR-325-3p in lungs, spleens, and livers from *in vivo*-transfected mice. Statistical significance between groups was determined by two-tailed Student’s *t* test or one-way ANOVA followed by Bonferroni *post hoc* test. All data are presented as the means ± SDs and were derived from three independent experiments. **, *P* < 0.01. n.s., not significant. Download FIG S1, TIF file, 0.4 MB. Copyright © 2020 Fu et al. 2020 Fu et al. This content is distributed under the terms of the Creative Commons Attribution 4.0 International license.

10.1128/mBio.00557-20.8 TABLE S1 Gross pathology of mice infected with M. tuberculosis. Download Table S1, DOCX file, 0.01 MB. Copyright © 2020 Fu et al. 2020 Fu et al. This content is distributed under the terms of the Creative Commons Attribution 4.0 International license.

### LNX1 is a direct target of miR-325-3p.

**FIG 2:**
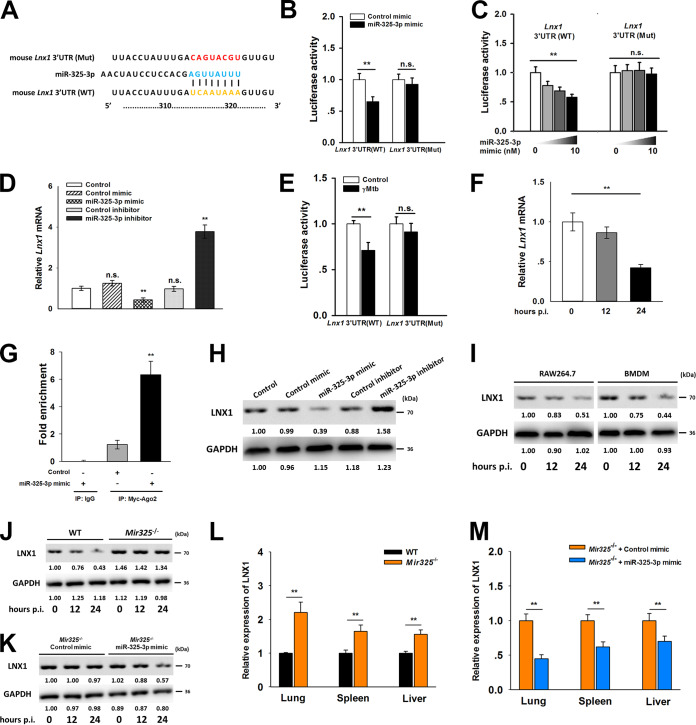
LNX1 is a direct target of miR-325-3p. (A) Predicted binding site of miR-325-3p and *Lnx1* 3′UTR. (B) Luciferase activities in HEK-293T cells cotransfected with miR-325-3p mimic and luciferase reporter constructs containing WT or MUT-type UTRs of *Lnx1*. (C) Luciferase activity changes in HEK-293T cells cotransfected with luciferase reporter constructs and different doses of miR-325-3p mimic. (D) Relative mRNA levels of *Lnx1* in RAW 264.7 cells transfected with miR-325-3p mimic or inhibitor. (E) Luciferase activities in RAW 264.7 cells transfected with luciferase reporter constructs and then infected with 10 μg/ml of gamma-irradiated M. tuberculosis for 24 h. (F) mRNA expression changes of *Lnx1* in RAW 264.7 cells during infection with 10 μg/ml of gamma-irradiated M. tuberculosis. (G) RAW 264.7 cells were transfected with Myc-tagged Ago2 in the presence or absence of miR-325-3p for 24 h. Immunoprecipitation was performed with Myc-antibody or IgG, and the enrichment of *Lnx1* mRNA was calculated by qRT-PCR. (H) Protein levels of LNX1 in HEK-293T cells transfected with miR-325-3p mimic or inhibitor. (I) LNX1 expression levels in RAW 264.7 cells and BMDMs during infection with 10 μg/ml of gamma-irradiated M. tuberculosis. (J) Expression levels of LNX1 in BMDMs from WT and *Mir325*^−/−^ mice after infection with 10 μg/ml of gamma-irradiated M. tuberculosis. (K) After infection with 10 μg/ml of gamma-irradiated M. tuberculosis, the expression levels of LNX1 in BMDMs from *Mir325*^−/−^ mice transfected with control mimic or miR-325-3p mimic were determined. (L) Relative expressions of LNX1 in lungs, spleens, and livers from WT and *Mir325*^−/−^ mice at 21 dpi. (M) Relative expressions of LNX1 in lungs, spleens, and livers from M. tuberculosis-infected *Mir325*^−/−^ mice transfected with miR-325-3p mimic *in vivo* at 21 dpi. Statistical significance between groups was determined by two-tailed Student’s *t* test or one-way ANOVA followed by Bonferroni *post hoc* test. All data are presented as the means ± SDs and were derived from three independent experiments. All blots are representative of three independent experiments. **, *P* < 0.01.

We next investigated whether miR-325-3p targets and regulates specific genes during M. tuberculosis infection. The targets of miR-325-3p were predicted by four microRNA databases (TargetScan Mouse 7.1, miRTarBase, miRDB, and miRcode) and GenBank to obtain reference sequences and functional annotations. A series of transcripts were found as potential targets of miR-325-3p, including *Gtf2a2*, *Cox7a1*, *Prss1*, *Tuba1c*, *Dgat1*, and *Lnx1*; *Lnx1* was selected for further study. *Lnx1* had a specific binding site for miR-325-3p in its 3′ untranslated region (3′ UTR) (Fig. 2A and Fig. S2). The 3′ UTR of *Lnx1* and its mutant were cloned into luciferase reporter vectors. Reporters were transfected into HEK293T cells along with the miR-325-3p mimic, and luciferase activities were measured. Luciferase activity was downregulated in the 3′ UTR of WT *Lnx1* but not in that of the mutant (Fig. 2B). When different concentrations of the miR-325-3p mimic were cotransfected with the WT *Lnx1* 3′ UTR into cells, this caused a downregulation of the luciferase activity in a dose-dependent manner (Fig. 2C). Consistent with luciferase activity results, there was a reverse correlation between miR-325-3p and *Lnx1* mRNA levels (Fig. 2D). In accordance with the upregulation of miR-325-3p during infection with gamma-irradiated M. tuberculosis, dual-luciferase assay and real-time quantitative PCR (qRT-PCR) were used to detect the transcription of *Lnx1* in response to stimulation with gamma-irradiated M. tuberculosis. Luciferase activity was downregulated in the presence of WT *Lnx1* 3′ UTR at 24 h postinfection (Fig. 2E). The *Lnx1* mRNA level was decreased with the prolongation of M. tuberculosis infection (Fig. 2F). RNA-induced silencing complex (RISC) uses miRNA as a template to recognize the complementary mRNA sequence and to activate Argonaute, and Argonaute 2 (Ago2) has been identified as the catalytic center of RISC in mice (17). Therefore, we immunoprecipitated Myc-tagged Ago2 in the presence of the miR-325-3p mimic in RAW 264.7 cells and found that there was a significant enrichment with *Lnx1* mRNA, suggesting that *Lnx1* is a direct target of miR-325-3p (Fig. 2G). In order to further confirm the role of miR-325-3p in regulating LNX1, LNX1 expression levels were evaluated by Western blotting. The results showed that LNX1 was suppressed in the presence of the miR-325-3p mimic but upregulated by the miR-325-3p inhibitor (Fig. 2H). To further confirm the results, BMDMs from WT mice and RAW 264.7 cells were infected with gamma-irradiated M. tuberculosis and the expression levels of LNX1 were measured. Infection with gamma-irradiated M. tuberculosis prevented the expression of LNX1 in both types of macrophages (Fig. 2I). This phenomenon did not appear in the BMDMs from *Mir325*^−/−^ mice (Fig. 2J). However, the downregulation of LNX1 was restored in the *Mir325*^−/−^ mice after transfection with the miR-325-3p mimic (Fig. 2K). In addition, the expression of LNX1 in organs also showed that LNX1 was upregulated in *Mir325*^−/−^ mice but not in *Mir325*^−/−^ mice transfected *in vivo* with the miR-325-3p mimic (Fig. 2L and M). These observations indicated that miR-325-3p targets LNX1 to suppress its expression during M. tuberculosis infection.

10.1128/mBio.00557-20.2 FIG S2 miR-325-3p targets LNX1 in different species. Shown is a schematic presentation of base pairing between the 3′ UTR of *Lnx1* and miR-325-3p in different species. Download FIG S2, TIF file, 0.2 MB. Copyright © 2020 Fu et al. 2020 Fu et al. This content is distributed under the terms of the Creative Commons Attribution 4.0 International license.

### LNX1 is the E3 ubiquitin ligase of NEK6.

**FIG 3:**
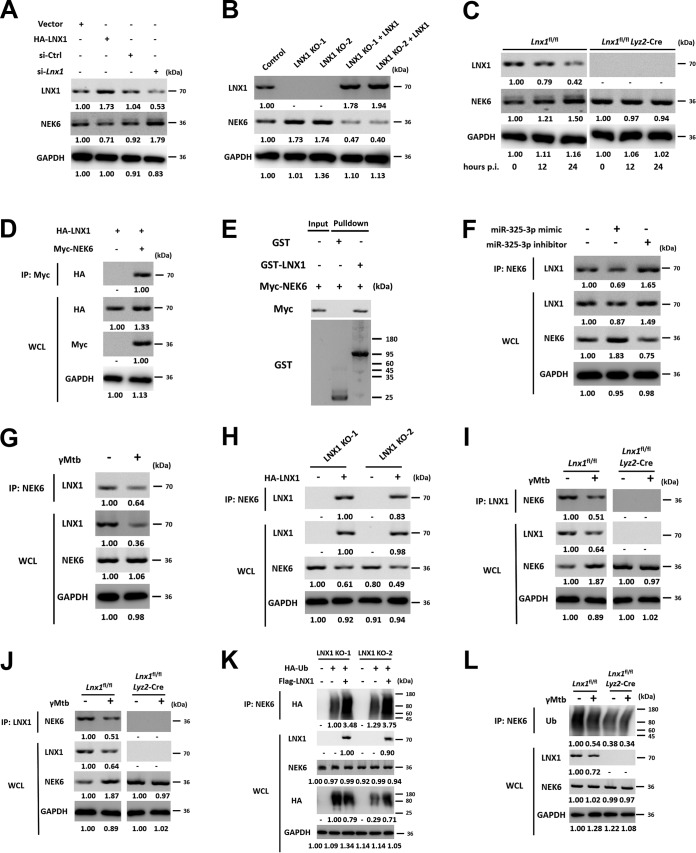
LNX1 is the E3 ubiquitin ligase of NEK6. (A) The expression of LNX1 and NEK6 in the presence of LNX1-overexpressing vector or *Lnx1* siRNA was analyzed using Western blotting. (B) The expression of LNX1 and NEK6 was detected in LNX1-deficient L929 cells and LNX1-deficient cells transfected with LNX1-overexpressing vectors. (C) Expression of LNX1 and NEK6 in BMDMs from *Lnx1*^fl/fl^ and *Lnx1*^fl/fl^*Lyz2*-Cre mice stimulated with 10 μg/ml of gamma-irradiated M. tuberculosis for 12 and 24 h. (D) HEK-293T cells were transfected with HA-tagged LNX1 and Myc-tagged NEK6, and cell lysates were harvested and immunoprecipitated using an anti-Myc antibody. The lower portion represents the immunoblot analysis of whole-cell lysates (WCL) without immunoprecipitation. (E) GST pulldown analysis of the interactions between GST-LNX1 and Myc-NEK6. (F) MiR-325-3p mimic or miR-325-3p inhibitor was transfected into L929 cells. Cell lysates were immunoprecipitated using an anti-NEK6 antibody. The recruitment of LNX1 was analyzed by immunoblotting. The lower portion represents the immunoblot analysis of whole-cell lysates. (G) RAW 264.7 cells were stimulated with 10 μg/ml of gamma-irradiated M. tuberculosis for 24 h. Immunoprecipitation was used to detect the interaction between NEK6 and LNX1. The lower portion represents the immunoblot analysis of whole-cell lysates. (H) The interaction of LNX1 and NEK6 was analyzed using immunoprecipitation in the presence and absence of LNX1. (I) BMDMs from *Lnx1*^fl/fl^ and *Lnx1*^fl/fl^*Lyz2*-Cre mice were stimulated with 10 μg/ml of gamma-irradiated M. tuberculosis for 24 h. The interaction of LNX1 and NEK6 was analyzed by immunoprecipitation. (J) Immunoprecipitation analysis of the polyubiquitination of NEK6 in HEK293T cells cotransfected with Flag-LNX1, Myc-NEK6, and HA-Ub (ubiquitin). (K) HA-Ub was cotransfected with and without Flag-LNX1 into LNX1-deficient L929 cells, and then the polyubiquitination of NEK6 was detected by immunoprecipitation. (L) BMDMs from *Lnx1*^fl/fl^ and *Lnx1*^fl/fl^*Lyz2*-Cre mice were infected with 10 μg/ml of gamma-irradiated M. tuberculosis for 24 h. Immunoprecipitation analysis of the polyubiquitination of NEK6 was carried out. MG132, a 26S proteasome inhibitor, was added during cell culture to inhibit the degradation of NEK6 in J-L. All blots are representative of three independent experiments.

To assess the functional effects of LNX1 during M. tuberculosis infection, we used the yeast two-hybrid system to screen the interacting proteins of LNX1. Since LNX1 has been reported as an E3 ubiquitin ligase (18), UbiBrowser and Ubiqsite were also used to predict potential targets and to search for the substrate of LNX1. Afterwards, potential candidates were further screened by Western blotting, and NEK6 was selected due to its low expression in M. tuberculosis-resistant macrophages after infection. NEK6 is a serine/threonine protein kinase which is ubiquitously expressed in several tissues (19–22), while the antituberculosis mechanism has not been reported. In order to determine the expression patterns of NEK6 and LNX1 in macrophages, hemagglutinin (HA)-tagged LNX1 or *Lnx1* small interfering RNA (siRNA) was transfected into RAW 264.7 cells. The results showed that the changes of NEK6 were negatively correlated with LNX1 (Fig. 3A). Transfection of miR-325-3p or infection with gamma-irradiated M. tuberculosis can also downregulate LNX1, resulting in the accumulation of NEK6 in macrophages (Fig. S3A and B). The CRISPR-Cas9 method was used to create LNX1-deficient L929 cells (LNX1 KO-1 and LNX KO-2). In the absence of LNX1, a higher expression level of NEK6 was observed. After transfection of an LNX1-overexpressing vector into LNX1-deficient cells, NEK6 was suppressed (Fig. 3B). Furthermore, we established myeloid cell-specific LNX1-deficient mice (*Lnx1*^fl/fl^*Lyz2*-Cre) and *Lnx1*^fl/fl^ littermates. BMDMs from *Lnx1*^fl/fl^*Lyz2*-Cre and *Lnx1*^fl/fl^ mice were infected with gamma-irradiated M. tuberculosis, and NEK6 levels were tested at the desired time points. The expression level of NEK6 showed no significant difference during infection in LNX1-deficient macrophages, while an upregulation of NEK6 was identified in cells from *Lnx1*^fl/fl^ mice (Fig. 3C). These data suggested that LNX1 is a negative regulator of NEK6.

10.1128/mBio.00557-20.3 FIG S3 LNX1 is the E3 ubiquitin ligase of NEK6. (A) The expression of LNX1 and NEK6 was analyzed by western blotting in the presence of miR-325-3p mimic or inhibitor. (B) The expression of LNX1 and NEK6 was analyzed in RAW 264.7 cells and BMDMs stimulated with 10 μg/ml of gamma-irradiated M. tuberculosis for 24 h. (C) Expression of UBE1 and UBCH5B in LNX1-deficient L929 cells. (D) Immunoprecipitation analysis of the polyubiquitination of NEK6 in L929 cells transfected with miR-325-3p mimic or inhibitor. (E) Polyubiquitination of NEK6 in RAW 264.7 cells stimulated with 10 μg/ml of gamma-irradiated M. tuberculosis for 24 h. (F) HA-LNX1 and Myc-NEK6 purified from transfected HEK293T cells were incubated with ATP, E1, E2, and ubiquitin. The *in vitro* ubiquitylation of NEK6 was analyzed by immunoblotting using an anti-Ub antibody. (G) Expression levels of *Nek6* mRNA in macrophages. MG132, the 26S proteasome inhibitor, was added during cell culture to inhibit the degradation of NEK6 in panels D and E. Statistical significance between groups was determined by two-tailed Student’s *t* test. All data are presented as the means ± SDs and were derived from three independent experiments. All blots are representative of three independent experiments. Download FIG S3, TIF file, 0.6 MB. Copyright © 2020 Fu et al. 2020 Fu et al. This content is distributed under the terms of the Creative Commons Attribution 4.0 International license.

Next, we investigated whether LNX1 directly bound to NEK6. HA-tagged LNX1 and Myc-tagged NEK6 were cotransfected into RAW 264.7 cells. Coimmunoprecipitation showed that LNX1 directly bound to NEK6 (Fig. 3D). An *in vitro* pulldown assay with Myc-NEK6 and glutathione *S*-transferase (GST)-LNX1 fusion protein also confirmed a direct interaction between LNX1 and NEK6 (Fig. 3E). To further define the LNX1-NEK6 interaction, L929 cells were treated with a synthetic miR-325-3p mimic or inhibitor. miR-325-3p overexpression inhibited NEK6-LNX1 interaction, whereas miR-325-3p inhibition increased NEK6-LNX1 interaction. The formation of LNX1-NEK6 complex was associated with LNX1 expression (Fig. 3F). This phenomenon was also observed in macrophages after stimulation with gamma-irradiated M. tuberculosis (Fig. 3G). In addition, LNX1-NEK6 complex could not be detected in LNX1-deficient cells unless HA-tagged LNX1 was transfected (Fig. 3H). Similarly, in LNX1-deficient BMDMs, LNX1-NEK6 complex was not immunoprecipitated. Meanwhile, NEK6 was not significantly upregulated after infection with gamma-irradiated M. tuberculosis (Fig. 3I), which was consistent with the findings detailed in Fig. 3C.

Ubiquitination is an important enzymatic posttranslational modification; it can induce proteasome-mediated protein degradations (11). Due to the reverse correlation between LNX1 and NEK6, the polyubiquitination of NEK6 in the presence or absence of LNX1 was studied. To better observe the polyubiquitination of NEK6, MG132, a 26S proteasome inhibitor, was used to inhibit the degradation of NEK6 by proteasomes. The results showed that LNX1 did not change the expressions of E1 and E2 (Fig. S3C), while the polyubiquitination of NEK6 was significantly promoted in the presence of LNX1 compared to that in the absence of LNX1 (Fig. 3J and K and Fig. S3D and E). Consistently, the *in vitro* ubiquitination assay confirmed that LNX1 directly targeted NEK6 (Fig. S3F). Moreover, gamma-irradiated-M. tuberculosis-induced downregulation of LNX1 with a decreasing polyubiquitination of NEK6 was observed in BMDMs from *Lnx1*^fl/fl^ mice, whereas this phenomenon did not occur in macrophages from *Lnx1*^fl/fl^*Lyz2*-Cre mice (Fig. 3L). In addition, we confirmed that *Nek6* mRNA level was not associated with LNX1 (Fig. S3G). Taken together, these data indicated that LNX1 is an E3 ubiquitin ligase that directly binds to NEK6 and then promotes the polyubiquitination and proteasome-mediated degradation of NEK6.

### LNX1 promotes K48-linked polyubiquitination of NEK6 at the K174 site.

**FIG 4:**
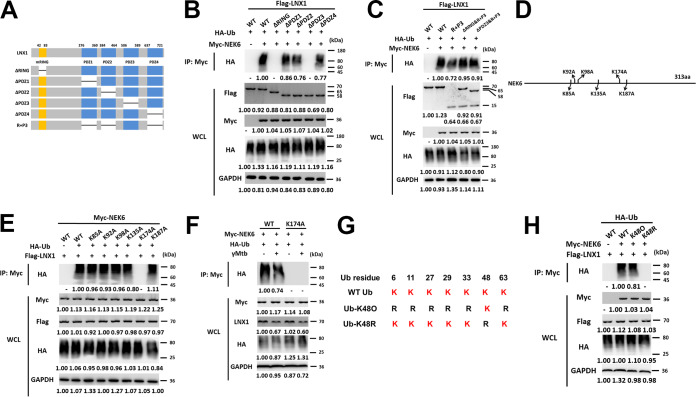
LNX1 promotes K48-linked polyubiquitination of NEK6 at K174 site. (A) A schematic diagram of LNX1 truncations. (B and C) Immunoprecipitation analysis of the polyubiquitination of NEK6 in LNX1 KO-1 cells cotransfected with Flag-tagged LNX1 truncations, HA-Ub, and Myc-NEK6. (D) A schematic diagram of the NEK6 ubiquitination site mutants. (E) Immunoprecipitation analysis of the polyubiquitination of NEK6 in HEK293T cells cotransfected with Myc-tagged NEK6 ubiquitination site mutants, HA-Ub, and Flag-LNX1. (F) K174A mutant of NEK6 was cotransfected with HA-Ub and Flag-LNX1 into RAW 264.7 cells stimulated with 10 μg/ml of gamma-irradiated M. tuberculosis for 24 h, and the polyubiquitination of NEK6 was detected by immunoprecipitation. (G and H) Schematic of WT or mutated Ub (G) and results from immunoprecipitation assays showing the specific lysine linked ubiquitin chains of NEK6 (H). MG132 was added during cell culture to inhibit the degradation of NEK6. All blots are representative of three independent experiments.

Next, we constructed domain truncations of LNX1 to investigate the LNX1-NEK6 interaction (Fig. 4A). In LNX1-deficient L929 cells, coimmunoprecipitation showed that the deletion of RING or PDZ3 domain in LNX1 impaired LNX1-mediated polyubiquitination of NEK6 (Fig. 4B and Fig. S4A). Transfection of RING and PDZ3 domains (R+P3) into LNX1-deficient cells restored the polyubiquitination of NEK6 (Fig. 4C and Fig. S4B). Further, lysine mutants (K85A, K92A, K98A, K135A, K174A, and K187A) of NEK6 were constructed to identify the recognition site of ubiquitin (Fig. 4D). Replacement of lysine with alanine at position 174 (K174A) abrogated LNX1-mediated polyubiquitination of NEK6 (Fig. 4E and Fig. S4C). The ubiquitination and proteasomal degradation pathway of NEK6 was impeded because of the downregulation of LNX1 in macrophages stimulated with gamma-irradiated M. tuberculosis (Fig. 4F); this was consistent with our previous findings (Fig. 3J and K). In contrast, the NEK6 K174A mutant could not be ubiquitinated during infection (Fig. 4F). Considering that different types of polyubiquitin link directly to distinct functions, we cotransfected NEK6 and LNX1 together with a series of ubiquitin mutants (K6O, K11O, K27O, K29O, K33O, K48O, and K63O) (23) into macrophages, each of which contained only one lysine available for polylinkage (Fig. 4G). The results showed that only the K48-linked polyubiquitination of NEK6 can be upregulated by LNX1. Meanwhile, the K48R ubiquitin mutant could not mediate polyubiquitination of NEK6 completely (Fig. 4H and Fig. S4D). Collectively, these results demonstrated that LNX1 promotes K48-linked polyubiquitination of NEK6 at the K174 site. The RING and PDZ3 domains of LNX1 are necessary for the polyubiquitination of NEK6.

10.1128/mBio.00557-20.4 FIG S4 LNX1 promotes K48-linked polyubiquitination of NEK6 at the K174 site. (A and B) Immunoprecipitation analysis of the polyubiquitination of NEK6 in LNX1 KO-2 cells cotransfected with Flag-tagged LNX1 truncations, HA-Ub and Myc-NEK6. (C) Immunoprecipitation analysis of the polyubiquitination of NEK6 in HEK293T cells cotransfected with Myc-tagged NEK6 ubiquitination site mutants, HA-Ub and miR-325-3p inhibitor. (D) A series of ubiquitin mutants (K6O, K11O, K27O, K29O, K33O, K48O, and K63O) were cotransfected with NEK6 and LNX1 into HEK293T cells, and an immunoprecipitation assay was used to screen the specific lysine-linked ubiquitin chains of NEK6. MG132 was added during cell culture to inhibit the degradation of NEK6. All blots are representative of three independent experiments. Download FIG S4, TIF file, 0.8 MB. Copyright © 2020 Fu et al. 2020 Fu et al. This content is distributed under the terms of the Creative Commons Attribution 4.0 International license.

### NEK6 inhibits apoptosis through activation of STAT3.

**FIG 5:**
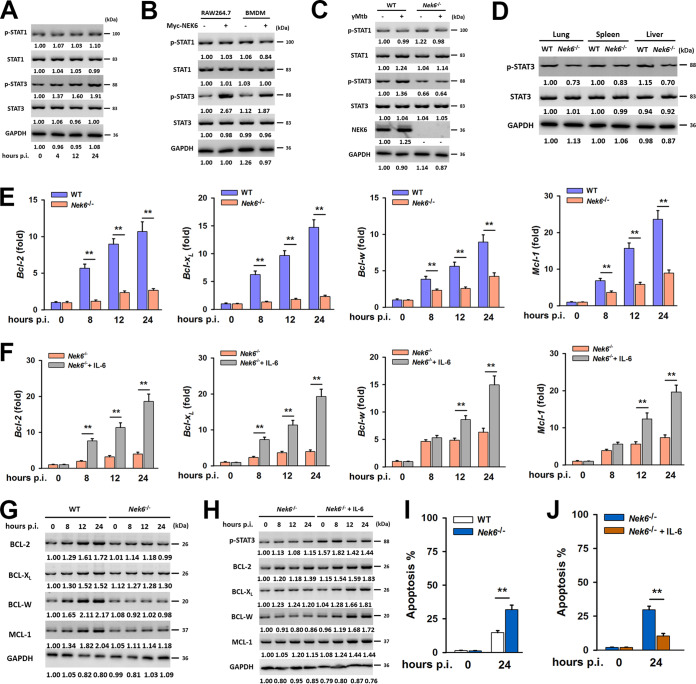
NEK6 inhibits apoptosis through the activation of STAT3. (A) Immunoblot analysis of the phosphorylated STAT1 (p-STAT1), STAT1, phosphorylated STAT3 (p-STAT3), and STAT3 in RAW 264.7 cells stimulated with 10 μg/ml of gamma-irradiated M. tuberculosis. (B) Immunoblot analysis of the p-STAT1, STAT1, p-STAT3, and STAT3 in RAW 264.7 cells and BMDMs transfected with Myc-NEK6. (C) BMDMs from WT and NEK6-deficient (*Nek6*^−/−^) mice were stimulated with 10 μg/ml of gamma-irradiated M. tuberculosis for 24 h. The expressions of p-STAT1, STAT1, p-STAT3, STAT3, and NEK6 were analyzed by Western blotting. (D) Immunoblot analysis of the p-STAT3 and STAT3 in lungs, spleens, and livers from WT and *Nek6*^−/−^ mice at 7 dpi. (E and G) The expression levels of BCL-2, BCL-X_L_, BCL-W, and MCL-1 in 10 μg/ml of gamma-irradiated M. tuberculosis-stimulated BMDMs from WT and *Nek6*^−/−^ mice were calculated by qRT-PCR at the indicated times (E) and by Western blotting (G). (F and H) BMDMs from *Nek6*^−/−^ mice were pretreated with 20 ng/ml IL-6 for 4 h h, then cells were stimulated with 10 μg/ml of gamma-irradiated M. tuberculosis. The expression levels of BCL-2, BCL-X_L_, BCL-W, and MCL-1 were calculated by qRT-PCR (F) and Western blotting (H) at the indicated times. (I) BMDMs from WT and *Nek6*^−/−^ mice were stimulated with 10 μg/ml of gamma-irradiated M. tuberculosis for 24 h, and the apoptosis rates were detected by flow cytometry. (J) BMDMs from *Nek6*^−/−^ mice were pretreated with 20 ng/ml of IL-6 for 4 h, and then cells were stimulated with 10 μg/ml of gamma-irradiated M. tuberculosis for 24 h. The apoptosis rates were detected by flow cytometry. Statistical significance between groups was determined by two-tailed Student’s *t* test. All data are presented as the means ± SDs and were derived from three independent experiments. All blots are representative of three independent experiments. **, *P* < 0.01.

We further investigated the specific role of NEK6 in the intracellular survival of M. tuberculosis. Since NEK6 is a protein kinase targeting STAT3 in mouse skin epidermal cells (24), we first focused on the regulation of the STAT pathway during M. tuberculosis infection. The phosphorylation of STAT3 was upregulated at 24 h postinfection (Fig. 5A). This result was consistent with the observed phosphorylation level of STAT3 with NEK6 overexpression or knockdown (Fig. 5B and Fig. S5A). *Nek6*^−/−^ mice showed a relatively lower level of phosphorylation of STAT3 both in BMDMs (Fig. 5C) and in organs (Fig. 5D) than the controls. STAT3 is a transcription activator that mediates the expression of a variety of genes in response to cell stimuli and thus plays a key role in many cellular processes, such as apoptosis and immune responses (25, 26). Therefore, we tested the downstream genes that could be directly regulated by STAT3 (Fig. S5B). Notably, BCL-2 and its family genes (BCL-X_L_, BCL-W, and MCL-1) were upregulated during infection with gamma-irradiated M. tuberculosis infection in BMDMs from WT mice but not in those from NEK6-deficient mice (Fig. 5E and G). On the other hand, proapoptotic genes (such as Bax, Bik, Bad, and Bak) were not affected in NEK6-deficient macrophages (Fig. S5C). To further confirm our findings, interleukin 6 (IL-6) was used to activate the STAT3 pathway in NEK6-deficient BMDMs (27). As a result, the expression levels of the BCL-2 family were restored (Fig. 5F and H). Due to the anti-apoptotic function of the BCL-2 family, NEK6-sufficient macrophages showed an inhibition of apoptosis, which was beneficial to the intracellular survival of M. tuberculosis (Fig. 5I and J). BCL-2 family genes regulate cell apoptosis by controlling mitochondrial membrane permeability (28). Based on this, we also found that NEK6-deficient BMDMs released cytochrome *c* from mitochondria to the cytoplasm and showed a high production of reactive oxygen species (ROS) (Fig. S5D to F). Taken together, these data indicated that NEK6 regulates innate immune responses through phosphorylation of STAT3 and participates in the regulation of apoptosis during M. tuberculosis infection.

10.1128/mBio.00557-20.5 FIG S5 NEK6 regulates immune response through activating STAT3. (A) Immunoblot analysis of p-STAT1, STAT1, p-STAT3, and STAT3 in RAW 264.7 cells and BMDMs transfected with *Nek6* siRNA. (B) BMDMs from wild-type (WT) and *Nek6*^−/−^ mice were stimulated with 10 μg/ml of gamma-irradiated M. tuberculosis for 24 h, and the relative expression and secretion of IL-6 and IL-10 were detected by qRT-PCR and enzyme-linked immunosorbent assay (ELISA) at the indicated times. (C) The expression levels of BAX, BCL-X_s_, BAD, and BAK in gamma-irradiated-M. tuberculosis (10 μg/ml)-stimulated BMDMs from WT and *Nek6*^−/−^ mice were calculated by qRT-PCR. (D and E) BMDMs from WT and *Nek6*^−/−^ mice were stimulated with 10 μg/ml of gamma-irradiated M. tuberculosis for 24 h, the relative reactive oxygen species (ROS) levels (D) and the ratios of GSH/GSSG (E) were detected. (F) BMDMs from WT and *Nek6*^−/−^ mice were stimulated with 10 μg/ml of gamma-irradiated M. tuberculosis for 24 h, and the cytochrome *c* in cytoplasm and mitochondria was analyzed by Western blotting. Statistical significance between groups was determined by two-tailed Student’s *t* test. All data are presented as the means ± SDs and were derived from three independent experiments. All blots were representative of three independent experiments. **, *P* < 0.01. Download FIG S5, TIF file, 0.8 MB. Copyright © 2020 Fu et al. 2020 Fu et al. This content is distributed under the terms of the Creative Commons Attribution 4.0 International license.

### Suppression of the NEK6/STAT3 pathway contributes to the defense against TB.

**FIG 6:**
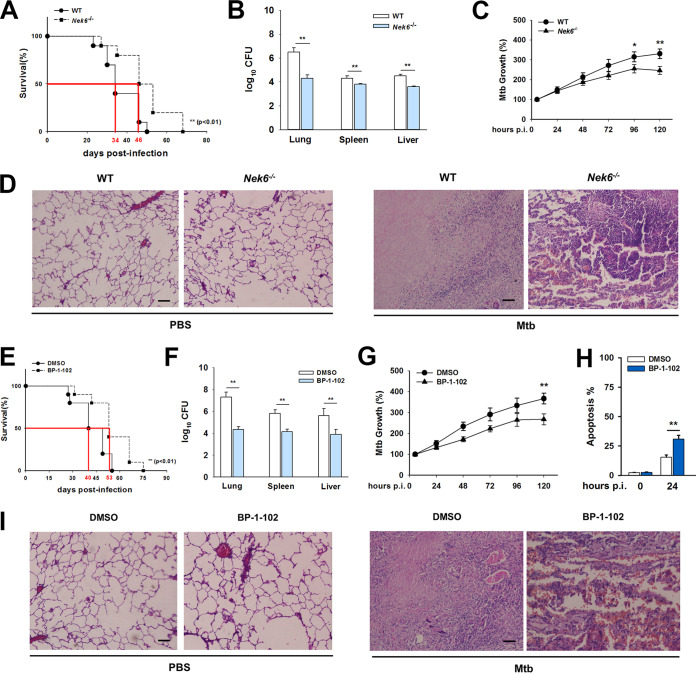
Suppression of NEK6/STAT3 pathway contributes to the defense against TB. (A) Survival of WT (*n* = 10) and *Nek6*^−/−^ mice (*n* = 10) after aerosol infection with approximately 400 CFU of M. tuberculosis. (B) M. tuberculosis bacterial loads in lungs, spleens, and livers of WT and *Nek6*^−/−^ mice at 21 dpi. (C) M. tuberculosis growth rates in BMDMs from WT and *Nek6*^−/−^ mice (MOI = 5). (D) Typical lung lesions of WT and *Nek6*^−/−^ mice at 21 dpi. Scale bar = 100 μm. (E) Survival of *Lnx1*^fl/fl^*Lyz2*-Cre mice (*n* = 10) and *Lnx1*^fl/fl^*Lyz2*-Cre mice orally administered BP-1-102 (3 mg/kg) (*n* = 10) after aerosol infection with approximately 400 CFU of M. tuberculosis. (F) M. tuberculosis bacterial loads in lungs, spleens, and livers of the *Lnx1*^fl/fl^*Lyz2*-Cre mice and *Lnx1*^fl/fl^*Lyz2*-Cre mice orally administered BP-1-102 (3 mg/kg) at 21 dpi. (G) M. tuberculosis growth rates in BMDMs from *Lnx1*^fl/fl^*Lyz2*-Cre mice and *Lnx1*^fl/fl^*Lyz2*-Cre mice treated with BP-1-102 (MOI = 5). (H) BMDMs from *Lnx1*^fl/fl^*Lyz2*-Cre mice were treated with BP-1-102 and infected with M. tuberculosis (MOI = 5) for 24 h; apoptosis rates were then detected by flow cytometry. (I) Typical lung lesions of the *Lnx1*^fl/fl^*Lyz2*-Cre mice and *Lnx1*^fl/fl^*Lyz2*-Cre mice orally administered BP-1-102 (3 mg/kg) at 21 dpi. Scale bar = 100 μm. Mouse survival data were plotted as Kaplan-Meier curves and compared by log rank (Mantel-Cox) test. Bacterial loads were analyzed using the Mann-Whitney U test. For other data, statistical significance between groups was determined by two-tailed Student’s *t* test. All data are presented as the means ± SDs and were derived from three independent experiments. *, *P* < 0.05; **, *P* < 0.01.

To better understand the role of the NEK6-mediated immune response to M. tuberculosis infection, we measured the survival rates of *Nek6*^−/−^ mice infected with M. tuberculosis. *Nek6*^−/−^ mice showed an improved median survival time compared to that of WT mice (Fig. 6A). Bacterial burdens were lower in the lungs, spleens, and livers of *Nek6*^−/−^ mice (Fig. 6B). In the BMDMs from *Nek6*^−/−^ mice, M. tuberculosis multiplied more slowly (Fig. 6C). Through pathology scoring, less pathological changes were observed in the lungs of *Nek6*^−/−^ mice, whereas WT mice showed larger necrotic lesions (Fig. 6D and Table S1). The fact that *Nek6*^−/−^ mice were more resistant to M. tuberculosis infection suggested that NEK6 contributed to the intracellular survival of M. tuberculosis. Moreover, to assess the effects of STAT3 on NEK6-mediated immune responses, a model of myeloid cell-specific LNX1-deficient mice (*Lnx1*^fl/fl^*Lyz2*-Cre) that has a higher NEK6 expression in BMDMs was constructed. LNX1-deficient mice were treated with oral administration of BP-1-102 (a selective STAT3 inhibitor that is orally bioavailable) to suppress the activation of STAT3 (29). BP-1-102 had no direct toxic effect on intracellular M. tuberculosis (Fig. S6). LNX1-deficient mice were treated with BP-1-102, and their littermates were infected with M. tuberculosis. Figure 6E to I and Table S1 show that LNX1-deficient mice were extremely susceptible to M. tuberculosis, with increased necrotic lesions in the lungs (Fig. 6I). However, LNX1-deficient mice treated with BP-1-102 showed suppression of STAT3 accompanying a better resistance to M. tuberculosis. All these findings suggested that NEK6 is a negative regulator in the antituberculosis immune responses through the excessive activation of STAT3.

10.1128/mBio.00557-20.6 FIG S6 BP-1-102 had no direct toxic effect on intracellular M. tuberculosis. Shown are M. tuberculosis growth rates in BMDMs from *Lnx1*^fl/fl^*Lyz2*-Cre mice oral administered BP-1-102 (3 mg/kg) (MOI = 5). Statistical significance between groups was determined by two-tailed Student’s *t* test. All data are presented as the means ± SDs and were derived from three independent experiments. Download FIG S6, TIF file, 0.09 MB. Copyright © 2020 Fu et al. 2020 Fu et al. This content is distributed under the terms of the Creative Commons Attribution 4.0 International license.

**FIG 7:**
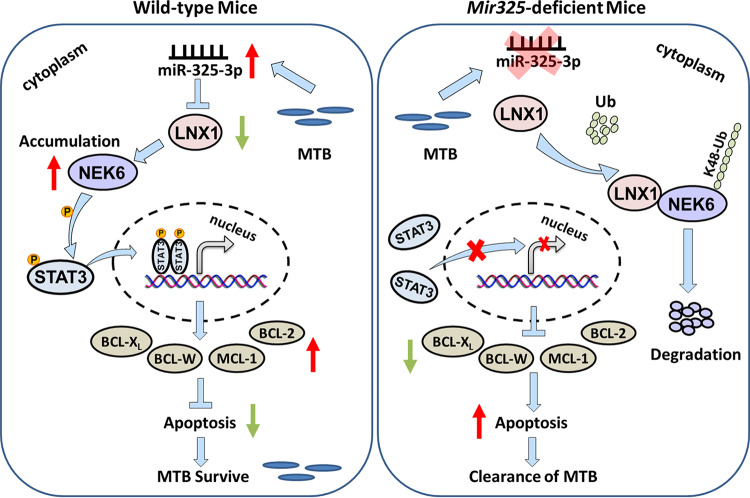
Schematic representation of miR-325-3p-facilitated immune escape of M. tuberculosis.

Collectively, our results indicate that we discovered a new immune escape pathway of M. tuberculosis by modulating host miR-325-3p expression. miR-325-3p targets LNX1 and results in the abnormal accumulation of NEK6, thus leading the inhibition of cell apoptosis and the intracellular survival of M. tuberculosis (Fig. 7).

## DISCUSSION

miRNAs play significant roles in regulatory mechanisms of various biological processes, including host-pathogen interactions. More than 30 miRNAs have been shown to participate in immune responses during M. tuberculosis infection (10). miR-155 is upregulated in TB patients and in experimental settings upon M. tuberculosis infection, while the regulatory effect of miR-155 on M. tuberculosis clearance is controversial (30–32). A lot of miRNAs are involved in regulating innate immune signaling pathways through targeting genes. Let-7 targets A20 to suppress innate immune responses (8), miRNA-27a targets ER-located Ca^2+^ transporter CACNA2D3 to inhibit autophagy (9), and miR-30a targets MyD88 to inhibit Toll-like receptor (TLR)/MyD88 activation and cytokine expression (33). In the previous study, we found that miR-325-3p was upregulated in a susceptible mouse substrain after aerosol infection with M. tuberculosis, yet there had been no interpretation of this phenomenon (data not published). In this study, we established mouse models and surprisingly, the survival time of M. tuberculosis-infected *Mir325*^−/−^ mice was significantly improved. This indicated that miR-325 might worsen the illness in M. tuberculosis infection.

The gene encoding LNX1, an E3 ubiquitin ligase, is one of the target genes of miR-325-3p. Human c-Src kinase is an important substrate of LNX1. The ubiquitination of c-Src by LNX1 has multiple effects on cell growth and cell differentiation (18, 34). In this study, we proved that LNX1 and its substrate, NEK6, were downstream of miR-325-3p during M. tuberculosis infection. miR-325-3p directly targets LNX1, resulting in the accumulation of NEK6. The LNX1-NEK6 axis demonstrates the role of posttranslational modifications in host-M. tuberculosis interaction. LNX1 contains a RING domain and four PDZ domains, which are necessary for the recognition of substrates. For instance, the ubiquitination of PTPRF interacting protein alpha 1 is dependent on a PDZ binding motif, which contains a carboxyl-terminal cysteine that binds to LNX1 PDZ2 (18). In this study, we illustrated that the RING and PDZ3 domains of LNX1 are necessary for the formation of the LNX1-NEK6 complex. Moreover, we showed that LNX1 catalyzes K48-linked polyubiquitination of NEK6 at the K174 site.

The NEK protein kinase subfamily can be identified in most eukaryotes, and its functions have been partially reported. For instance, NLRP3 inflammasome activation requires the interaction of NLRP3 with centrosomal kinase NEK7 (35). NEK6 is a protein kinase of CDK7, HSP70, and STAT3 (24, 36, 37). The accumulation of NEK6 certainly phosphorylated STAT3. In the classic JAK/STAT3 pathway, Tyr705 is a readout of JAK/STAT signaling (38). NEK6 may compete with JAK in STAT3 signaling. Future studies are needed to clarify the mechanisms by which NEK6 accumulation leads to the phosphorylation of STAT3 at Tyr705. As a transcriptional regulator, STAT3 has key roles in vertebrate development and mature tissue function, including the control of inflammation and immunity (39). STAT3 signaling is a major intrinsic pathway for inflammation. The continuous activation of STAT3 in tumor cells increases the expression of cytokines (transforming growth factor β [TGF-β] and IL-10) and then promotes tumor development and metastasis in a positive-feedback manner (40). Also, STAT3-deficient macrophages, neutrophils, and dendritic cells (DCs) produce elevated amounts of proinflammatory cytokines upon TLR4 activation, including tumor necrosis factor alpha (TNF-α), IL-6, IL-12, and gamma interferon (IFN-γ) (41, 42). However, in our research, the activation of STAT3 by NEK6 inhibited cell apoptosis through regulation of the transcription of anti-apoptotic BCL-2 family genes. Apoptotic cell death is one of the most important manners for the macrophages to eliminate the intracellular M. tuberculosis. The inhibition of apoptosis gives M. tuberculosis a chance to develop latent infection (43). Notably, BP-1-102, a selective inhibitor of STAT3, was proved to relieve pulmonary nodules in M. tuberculosis-infected mice through oral administration. This result provides a potential therapeutic approach to TB patients. Interestingly, we found that not only proinflammatory factor IL-6 but also anti-inflammatory factor IL-10 was raised by the phosphorylated STAT3 during M. tuberculosis infection. Accordingly, the involvement of inflammation with the NEK6-mediated immune escape of M. tuberculosis requires further investigation.

Together, our findings showed that miR-325-3p participates in the regulation of apoptosis during M. tuberculosis infection through targeting LNX1. According to the previous study, miR-325-3p directly targets RIPK3 to program necrosis in mice (44). Silencing of RIPK3 increases the viability of cardiomyocytes under hypoxic conditions (44). Apoptosis or necrosis, the outcome of the encounter between macrophages and M. tuberculosis, is known to be the ultimate factor to influence the susceptibility of the host to M. tuberculosis. It would be interesting to investigate further whether RIPK3 is involved in the immune responses of the host during M. tuberculosis infection.

## MATERIALS AND METHODS

### Ethics statement.

This study was carried out in strict accordance with the guidelines for the care and use of animals of Chongqing University. All animal experimental procedures were approved by the Animal Ethics Committees of the School of Life Sciences, Chongqing University.

### Mice and treatment.

C57BL/6N and stock *Mir325^tm1Mtm^*/Mmjax mice were purchased from Jackson Laboratory. C57BL/6N-*Nek6^tm1cyagen^* mice (KOCMP-03942-NEK6) were purchased from Cyagen Biosciences (Guangzhou, China). *In vivo* transfection was performed by tail vein injection [N/P ratio (the number of nitrogen residues of *in vivo* − jetPEI reagent per nucleic acid phosphate) = 8] using the jetPEI *in vivo* transfection reagent (Polyplus Transfection Inc., New York, NY) according to the manufacturer’s instructions as previously described (45, 46). Injections were repeated 3 times per week for living mice. To generate myeloid cell-specific LNX1-deficient (*Lnx1*^fl/fl^*Lyz2*-Cre) mice (C57BL/6N background), we hybridized *Lnx1*^ﬂ/ﬂ^ mice (containing the *loxP* sequence ﬂanking 2 exon of *Lnx1*) with *Lyz2*-Cre mice. We used 8-week-old *Lnx1*^ﬂ/ﬂ^*Lyz2*-Cre mice and *Lnx1*^ﬂ/ﬂ^ littermates. To inhibit the activity of STAT3, *Lnx1*^fl/fl^*Lyz2*-Cre mice were orally administered BP-1-102 (3 mg/kg of body weight) as previously described (29).

### Cell culture and treatment.

RAW 264.7, HEK293T, and L929 cells were purchased from the American Type Culture Collection (ATCC; Manassas, VA) and were grown adherently to plastic plates (Costar; Corning, Cambridge, MA). The culture medium was composed of Dulbecco’s modified Eagle’s medium (DMEM; Gibco, San Jose, CA) and 10% fetal bovine serum (FBS; Gibco). BMDMs were obtained by culturing bone marrow cells as previously described (47). In brief, bone marrow cells were cultured in RPMI 1640 (Gibco) supplemented with 10% heat-inactivated FBS, 2 mM l-glutamine, 1% nonessential amino acids, 100 U of penicillin, 100 μg of streptomycin, and 22 ng of macrophage colony-stimulating factor (M-CSF; PeproTech, Rocky Hill, NJ)/ml for 6 days at 37°C and 5% CO_2_. After 6 days of culture, adherent macrophages were switched into antibiotic-free media and seeded at 10^5^ per well. Plasmid DNA and synthetic siRNA were transfected into cells using Lipofectamine 3000 transfection reagent (Invitrogen, Life Technologies, CA). For IL-6 treatment, BMDMs were obtained by culturing bone marrow cells from C57BL/6N-*Nek6^tm1cyagen^* mice. A total of 20 ng/ml of IL-6 was used to pretreat BMDMs for 4 h before infection with gamma-irradiated M. tuberculosis.

### Bacterial culture and infection.

M. tuberculosis strain H37Rv (ATCC) was grown in Middlebrook 7H9 (BD Biosciences, San Jose, CA) medium supplemented with 10% oleic acid-albumin-dextrose-catalase (OADC; BD Biosciences) and 0.05% Tween 80 (Sigma-Aldrich, St. Louis, MO). Bacterial colonies were counted on Middlebrook 7H10 agar (BD Biosciences) supplemented with 10% OADC after incubation at 37°C for 21 days. Eight-week-old mice were aerosol infected with approximately 400 CFU of M. tuberculosis using a Glas-Col inhalation exposure system (Glas-Col Inc., Terre Haute, IN) and killed at the desired time points. Cells were infected with M. tuberculosis at a multiplicity of infection (MOI) of 5 for the desired time or treated with 10 μg/ml of gamma-irradiated M. tuberculosis (strain H37Rv; BEI Resources, Manassas, VA) for 24 h.

### CFU assay.

The organs were harvested at the desired time points. A CFU assay was performed by plating 10-fold serial dilutions of each tissue homogenate on Middlebrook 7H10 agar plates. The colonies were counted after 4 to 6 weeks of incubation at 37°C in 5% CO_2_.

### Pathology scoring system.

Pathology scores were assigned to the lungs of mice as previously described, with partial modification (48). Lung lobes were sliced to examine lesions and evaluate them using a semiquantitative gross pathology scoring system as follows: 0, no visible lesions; 1, no external gross lesions but lesions seen upon slicing; 2, fewer than five gross lesions <4 mm in diameter; 3, more than five gross lesions <4 mm in diameter; 4, more than one distinct gross lesion >4 mm in diameter; and 5, gross coalescing lesions. The scores of the individual lobes were added to calculate the lung score. The typical lesions were fixed with 10% buffered formaldehyde for more than 24 h, embedded in paraffin, sectioned, and stained with hematoxylin and eosin (H&E) according to the standard procedure. Photographs were obtained by microscopy (Carl Zeiss, Jena, Germany).

### Plasmid construction.

The 3′ UTR of *Lnx1* (NCBI accession number NM_001159578) was amplified and inserted the psicheck-2 vector (Promega, Madison, WI) through standard molecular cloning methods and confirmed by sequencing. Full-length coding sequences of LNX1 and NEK6 (NCBI accession number NM_001159631) were inserted into pCMV-HA, pCMV-Myc, pFLAG-CMV-4, or pGEX-4T-1. The K48R, K63R, and K48/63R mutants were kindly provided by Yonghui Zheng (Michigan State University). The WT ubquitin and mutants were purchased from Addgene. All the primers used for plasmids construction are listed in Table S2.

10.1128/mBio.00557-20.9 TABLE S2 Primers used for plasmid construction. Download Table S2, DOCX file, 0.01 MB. Copyright © 2020 Fu et al. 2020 Fu et al. This content is distributed under the terms of the Creative Commons Attribution 4.0 International license.

### Real-time quantitative PCR.

To analyze gene expression, total RNA samples were isolated using TRIzol reagent (Invitrogen). Purified RNA was reverse transcribed using a SYBR PrimeScript RT-PCR kit (TaKaRa, Otsu, Shiga, Japan). The expression of mRNAs was quantified using a SYBR Premix *Ex Taq* II kit (TaKaRa). qRT-PCR was performed on an ABI StepOnePlus PCR system (Applied Biosystems, Foster City, CA), and the results were normalized to *Gapdh* mRNA levels. To analyze miRNA expression, total RNA samples were isolated using a mirVana miRNA isolation kit (Ambion, Austin, TX). Purified RNA was reverse transcribed using a miScript II RT kit (Qiagen, Hilden, Germany). The expression levels of mature miRNAs were quantified using a miScript SYBR green PCR kit containing 10× miScript universal primer (Qiagen) according to the manufacturer’s instructions. Quantization of U6 was performed to normalize miRNA expression levels. Data were analyzed using the threshold cycle (2^−ΔΔ^*^CT^*) method. All primer sequences used for qRT-PCR are listed in Table S3.

10.1128/mBio.00557-20.10 TABLE S3 Primers used for qRT-PCR. Download Table S3, DOCX file, 0.01 MB. Copyright © 2020 Fu et al. 2020 Fu et al. This content is distributed under the terms of the Creative Commons Attribution 4.0 International license.

### Immunoprecipitation and immunoblot analysis.

Cell lysates were fractionated by sodium dodecyl sulfate-polyacrylamide gel electrophoresis and transferred to polyvinylidene fluoride membranes (Millipore, Bedford, MA). Blots were probed with 1/1,000 anti-glyceraldehyde-3-phosphate dehydrogenase (anti-GAPDH; AF0006), 1/1,000 anti-COXIV (AC610), 1/1,000 anti-GST (AF2299), 1/1,000 anti-Flag (AF0036) (Beyotime, Jiangsu, China), 1/500 anti-LNX1 (AV43367), 1/500 anti-NEK6 (AV48756), 1/1,000 anti-HA (SAB2702196), 1/1,000 anti-Myc (SAB2702192), 1/500 anti-UBA1 (HPA000289) (Sigma-Aldrich), 1/500 anti-ubiquitin (sc-271289) (Santa Cruz Biotechnology, Santa Cruz, CA), 1/500 anti-STAT1 (AHO0832), 1/200 anti-phospho-STAT1 (Tyr701) (44-376G), 1/500 anti-STAT3 (MA1-13042), 1/200 anti-phospho-STAT3 (Tyr705), 1/500 anti-BCL-2 (MA5-11757), 1/200 anti-BCL-X_L_ (MA5-15142), 1/200 anti-BCL-W (PA5-78865), 1/200 anti-MCL1 (PA5-86174), 1/500 anti-cytochrome *c* (45-6100), and 1/200 anti-UBE2D2 (PA5-86740) (Invitrogen). Immunoblots were revealed using SuperSignal West Pico substrate (Thermo Fisher Scientific, San Jose, CA). The relative intensity of protein bands were quantified using Image J software and normalized to the controls. For immunoprecipitation (IP), cells were collected and lysed with 1× lysis buffer (Thermo Fisher Scientific), and the lysates were subsequently incubated with appropriate antibodies and protein A/G beads (Thermo Fisher Scientific) overnight at 4°C. The beads were washed three times with IP buffer (Thermo Fisher Scientific), followed by immunoblot analysis. The original blot data are listed in Fig. S7.

10.1128/mBio.00557-20.7 FIG S7 Original data of immunoprecipitation and immunoblot analysis. Download FIG S7, PDF file, 1.8 MB. Copyright © 2020 Fu et al. 2020 Fu et al. This content is distributed under the terms of the Creative Commons Attribution 4.0 International license.

### Luciferase assays.

A dual-luciferase reporter (DLR) assay system (Promega) was used to perform luciferase assays as previously described (48). In brief, cells were cotransfected with luciferase reporter plasmid and internal control plasmid pRL-SV40. Cells were lysed for DLR assays 24 h after treatment. Data were collected with a VICTOR X5 multilabel plate reader (PerkinElmer, Waltham, MA), and relative luciferase activities were measured by firefly luciferase luminescence divided by *Renilla* luciferase luminescence.

### Flow cytometry.

Annexin V staining, paired with propidium iodide (PI), was used to identify apoptotic cells with an annexin V-fluorescein isothiocyanate (FITC) apoptosis detection kit (BD Biosciences). Flow cytometry analysis was performed according to standard procedures.

### Statistical analysis.

All data were derived from at least three independent experiments and are presented as the means ± standard deviations (SD) unless otherwise indicated. Two-tailed Student’s *t* test was used to compare the means between two groups, and one-way analysis of variance (ANOVA) followed by Bonferroni *post hoc* test was used for multiple comparisons. Mouse survival data were plotted as Kaplan-Meier curves and compared by log rank (Mantel-Cox) test. Bacterial loads were analyzed using the Mann-Whitney U test. All blots are representative of three independent experiments. A *P* value of <0.05 was considered significant.
