# Supplementary figures and images for: MicroRNA-325-3p Facilitates Immune Escape of Mycobacterium tuberculosis through Targeting LNX1 via NEK6 Accumulation to Promote Anti-Apoptotic STAT3 Signaling
Source: mBio. 2020 Jun 2;11(3):e00557-20. doi: 10.1128/mBio.00557-20 (PMC7267881; doi:10.1128/mBio.00557-20)

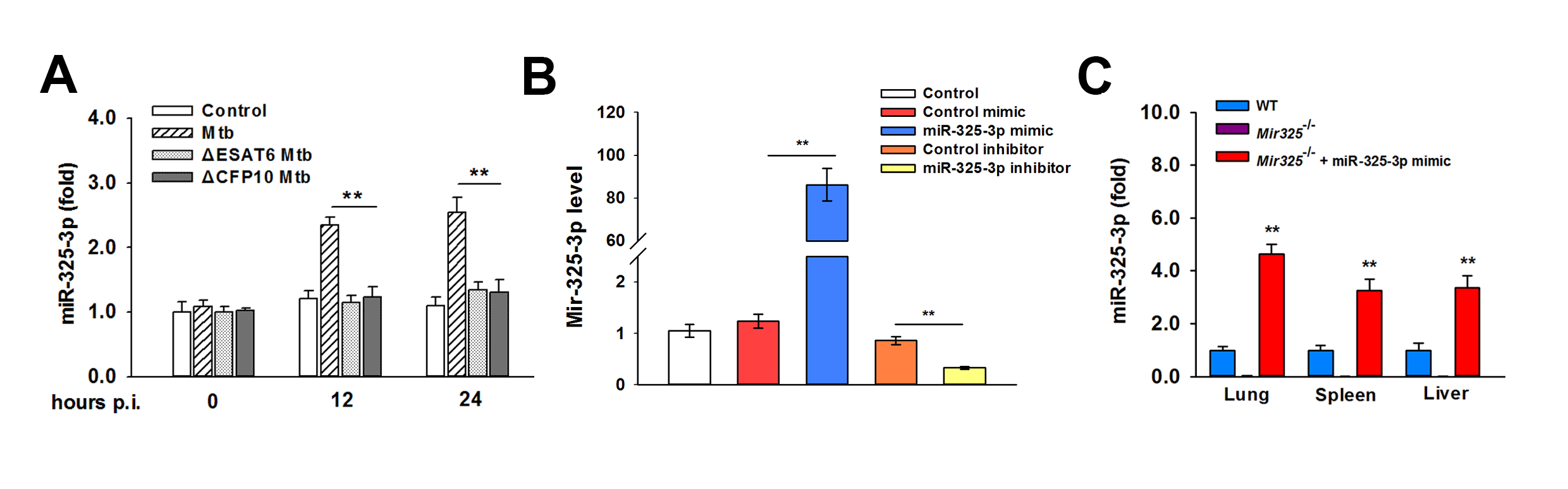

Supplement: FIG S1 [file mBio.00557-20-sf001.tif]

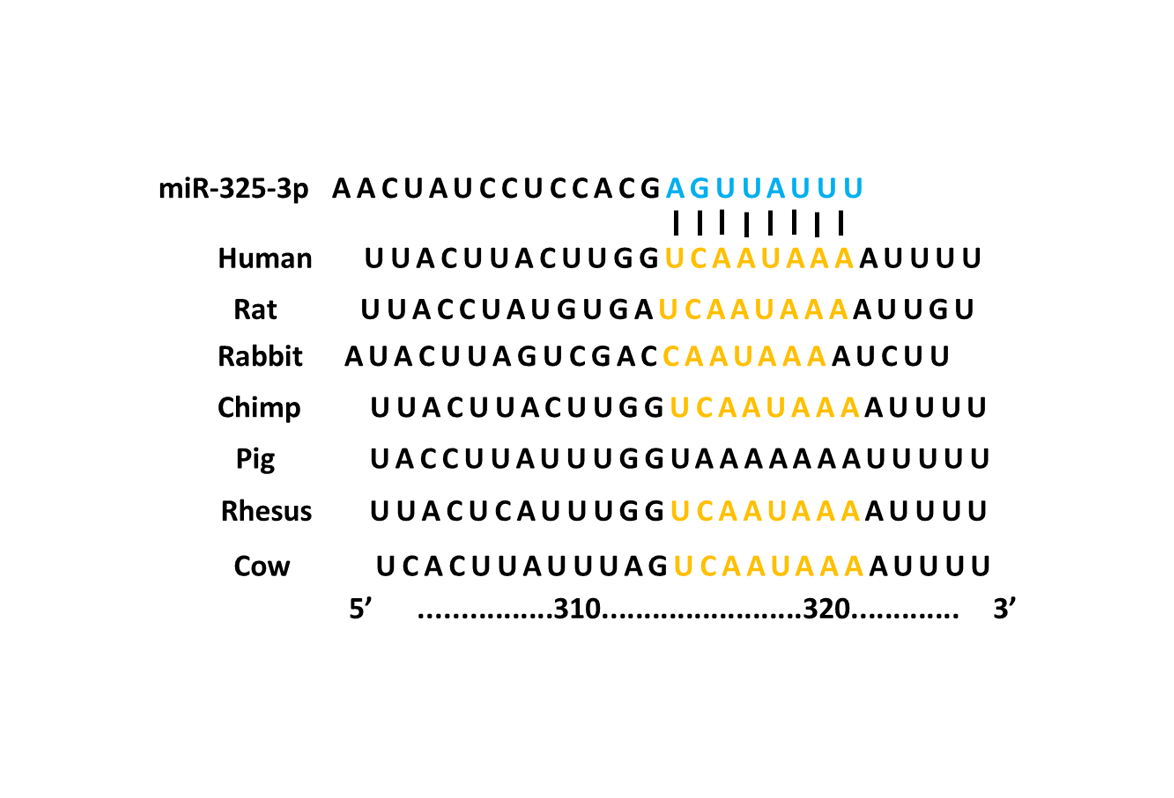

Supplement: FIG S2 [file mBio.00557-20-sf002.tif]

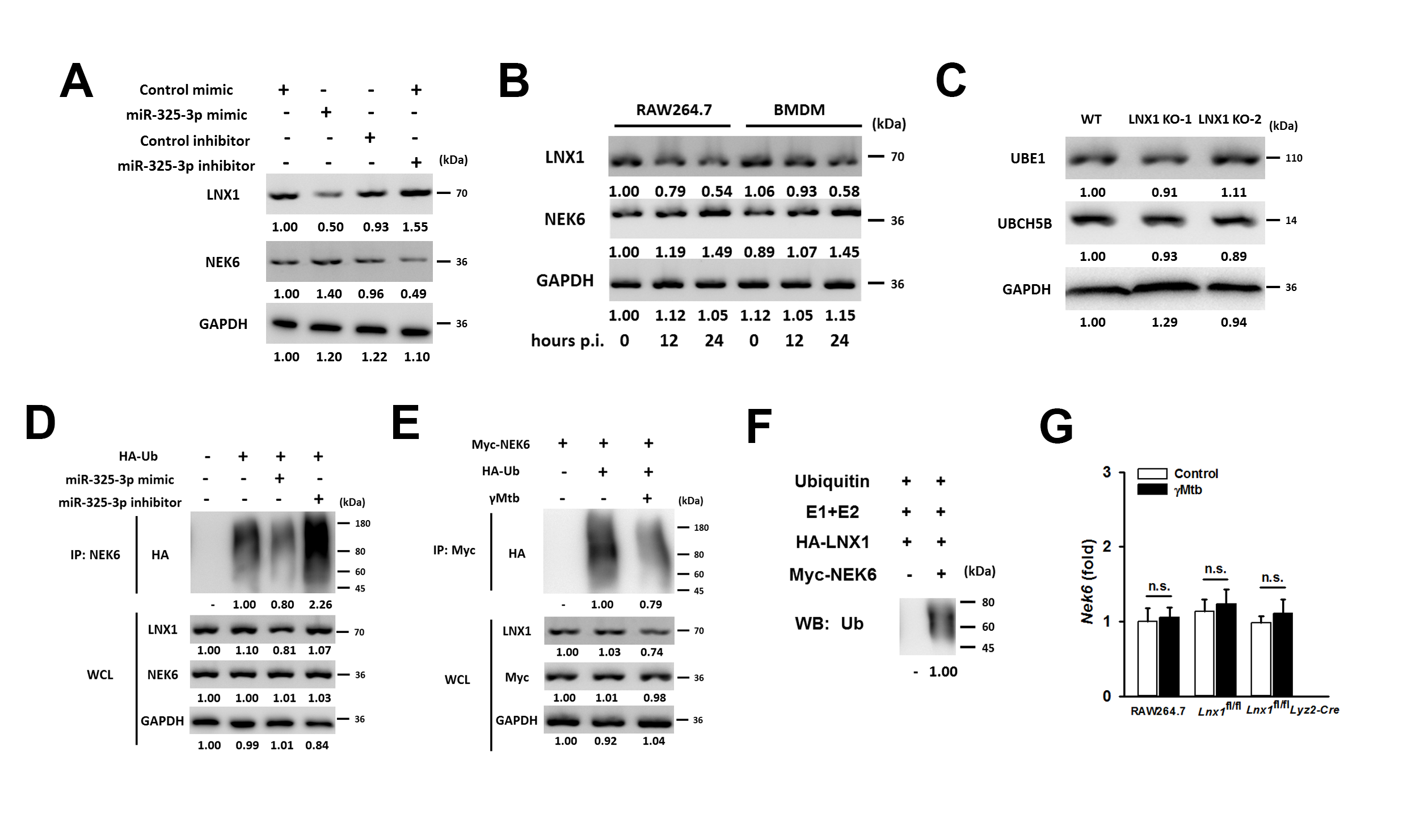

Supplement: FIG S3 [file mBio.00557-20-sf003.tif]

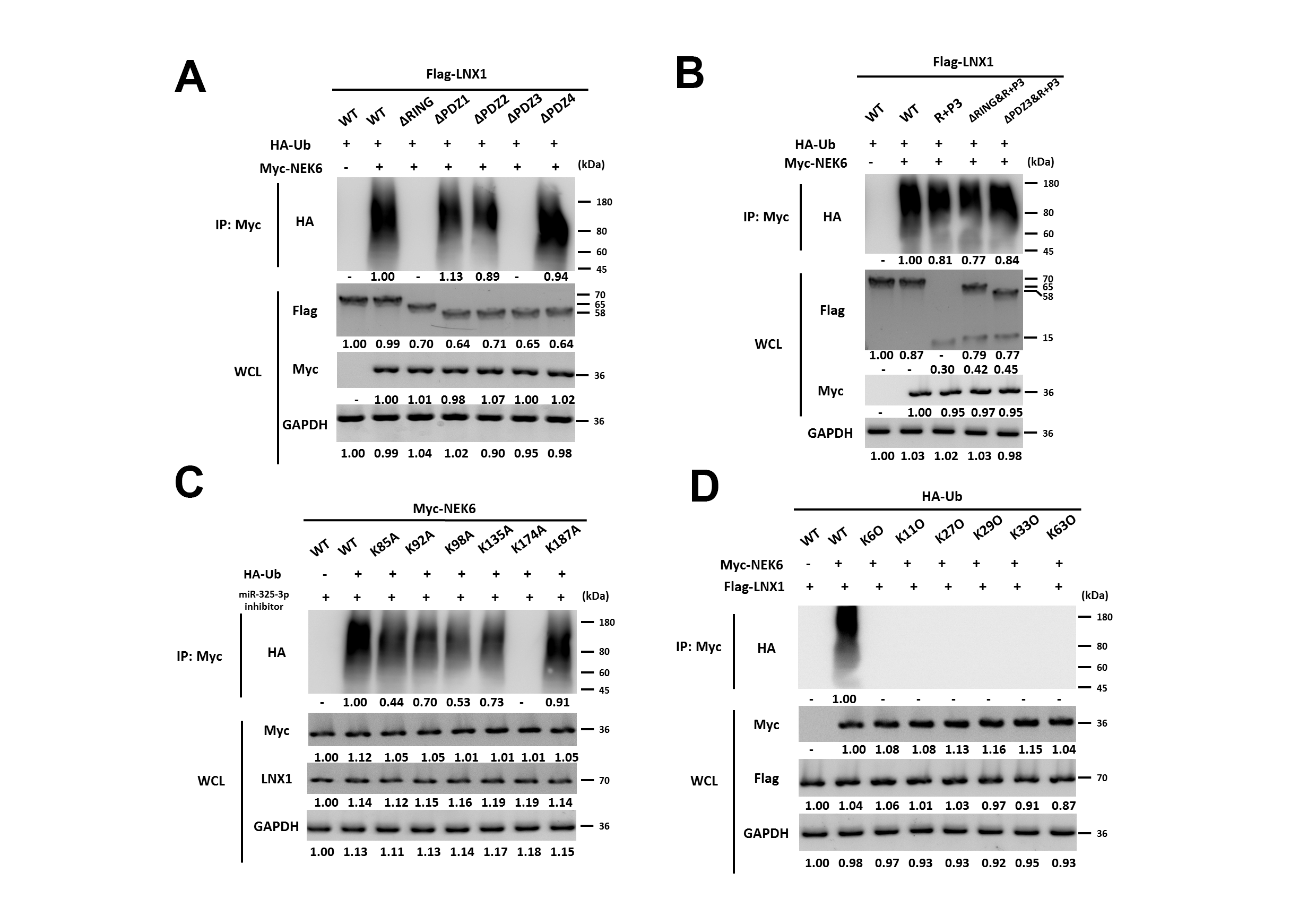

Supplement: FIG S4 [file mBio.00557-20-sf004.tif]

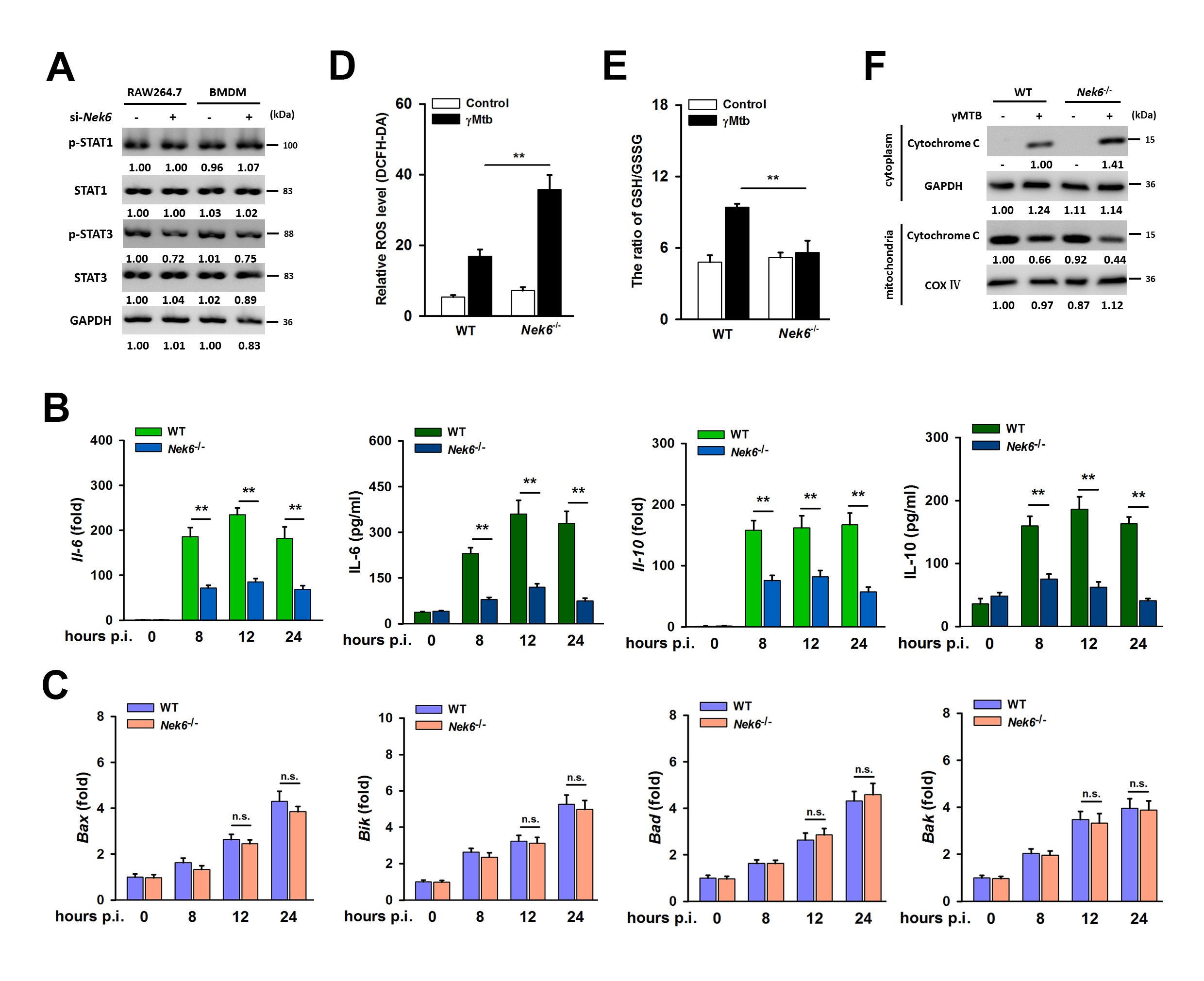

Supplement: FIG S5 [file mBio.00557-20-sf005.tif]

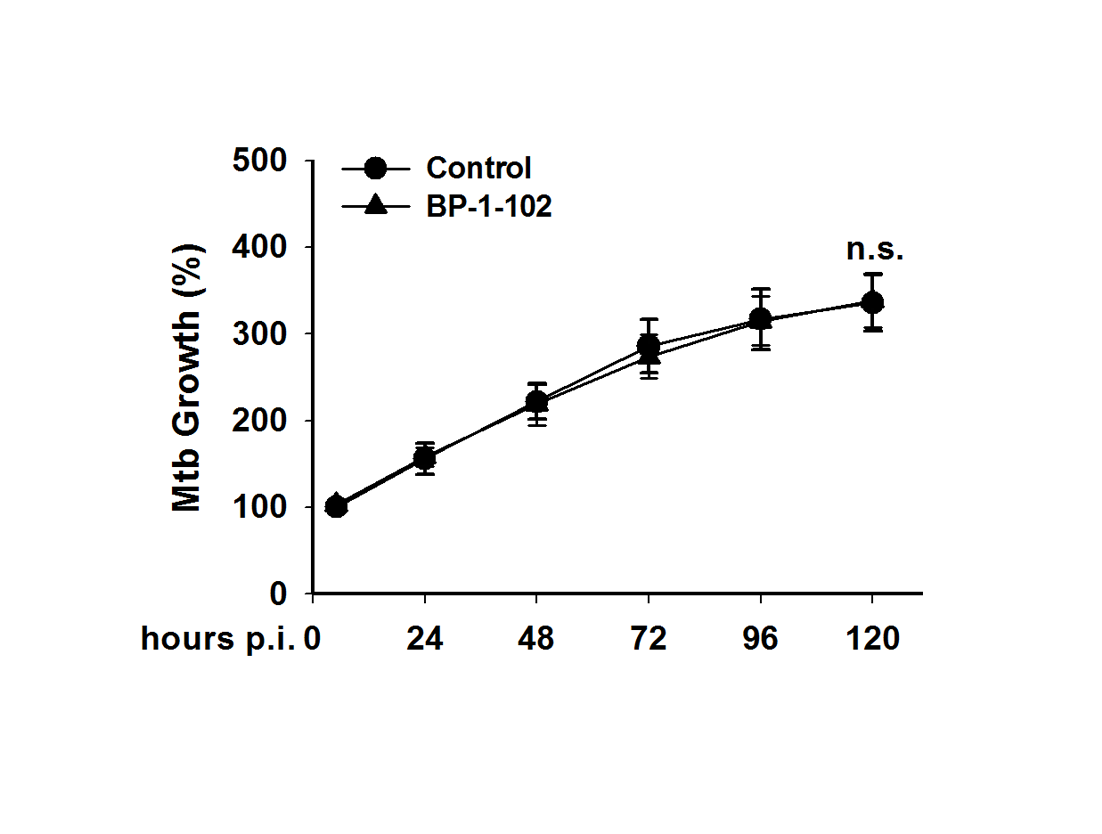

Supplement: FIG S6 [file mBio.00557-20-sf006.tif]
