# Supplementary material for: MicroRNA-325-3p Facilitates Immune Escape of Mycobacterium tuberculosis through Targeting LNX1 via NEK6 Accumulation to Promote Anti-Apoptotic STAT3 Signaling
Source: mBio. 2020 Jun 2;11(3):e00557-20. doi: 10.1128/mBio.00557-20 (PMC7267881; doi:10.1128/mBio.00557-20)
Supplement: TABLE S1 [file mBio.00557-20-st001.docx]

**Table S1** **Gross pathology of mice infected with Mtb**

| **Animal Group** | **Lung Score** | **Mean ± SD*** |
| --- | --- | --- |
| WT mice | 13 | 12.67±2.52 |
|  | 10 |  |
|  | 15 |  |
| *Mir325*^-/-^ mice | 7 | 6.33±1.15 |
|  | 5 |  |
|  | 7 |  |
| *Mir325*^-/-^ mice transfected with miR-325-3p mimic | 10 | 10.00±1.00 |
|  | 11 |  |
|  | 9 |  |
| *Nek6*^-/-^ mice | 3 | 4.67±1.53 |
|  | 5 |  |
|  | 6 |  |
| *Lnx1*^fl/fl^*Lyz2*-Cre mice | 14 | 15.67±1.53 |
|  | 16 |  |
|  | 17 |  |
| *Lnx1*^fl/fl^*Lyz2*-Cre mice oral administrated with BP-1-102 | 5 | 4.67±0.58 |
|  | 5 |  |
|  | 4 |  |

***Median values per group (n = 3)**
