# Supplementary material for: MicroRNA-325-3p Facilitates Immune Escape of Mycobacterium tuberculosis through Targeting LNX1 via NEK6 Accumulation to Promote Anti-Apoptotic STAT3 Signaling
Source: mBio. 2020 Jun 2;11(3):e00557-20. doi: 10.1128/mBio.00557-20 (PMC7267881; doi:10.1128/mBio.00557-20)
Supplement: TABLE S2 [file mBio.00557-20-st002.docx]

**Table S2** **Primers used for plasmids construction**

| **Name** | **Forward** | **Reverse** |
| --- | --- | --- |
| psicheck-2-Lnx1 3’UTR WT | AATCCTCGAGCCACAGATAGGCTAGG | TATAAGCGGCCGCCTGTTCTGAGCCA |
| psicheck-2-Lnx1 3’UTR mut | TACCTATTTGACAGTACGTGTTGTA | TAATACAACACGTACTGTCAAATAG |
| Myc-Ago2 | AACGGAATTCCCACCATGTACTCGGG | TCAAGCGGCCGCATTAAAGTGTTTTAA |
| HA-LNX1 | AATCCTCGAGCCATGAACCAACCGGAC | AATAAGCGGCCGCAATATCATACTTTTCT |
| GST-LNX1 | AATAGAATTCGCCATGAACCAACCGGAC | ATAAGTCGACTCTGGCATTTTTTACAATAT |
| Flag-LNX1 | AATAGAATTCCATGAACCAACCGGACCT | ATAAGGTACCGCATTTTTTACAATATATT |
| Myc-NEK6 | AATAGAATTCGGATGGCAGGACAGCCCA | ATAACTCGAGTGCAAAGGCTGGCCCACA |
