# Supplementary material for: MicroRNA-325-3p Facilitates Immune Escape of Mycobacterium tuberculosis through Targeting LNX1 via NEK6 Accumulation to Promote Anti-Apoptotic STAT3 Signaling
Source: mBio. 2020 Jun 2;11(3):e00557-20. doi: 10.1128/mBio.00557-20 (PMC7267881; doi:10.1128/mBio.00557-20)
Supplement: TABLE S3 [file mBio.00557-20-st003.docx]

**Table S3** **Primers used for qRT-PCR**

| **Name** | **Forward** | **Reverse** |
| --- | --- | --- |
| pri-miR-325 | TTTGCTTCTGTTTCCTG | AGATGCTCCCTTTTGAG |
| pre-miR-325 | CTTGGTTCCTAGTAGGTGCT | CCAGAGCCTAGCACAGTGC |
| miR-325-3p | TTTATTGAGCACCTCCTATCAA | Universal Primer |
| miR-325-5p | CCTAGTAGGTGCTCAGTAAGTGT | Universal Primer |
| U6 | CTCGCTTCGGCAGCACA | AACGCTTCACGAATTTGCGT |
| *Lnx1* | GCAGGGACAACTCTGAGAAC | GCGATAAATATGCTGGATGA |
| *Bcl-2* | CGGGAGAACAGGGTATGA | CAGGCTGGAAGGAGAAGA |
| *Bcl-x_l_* | CTCTTTCGGGATGGAGTAAA | TAGGTGGTCATTCAGATAGGT |
| *Bcl-w* | TGTGGCATTCTTTGTCTTTG | CAGACGTGTCTCCAGGTAGG |
| *Mcl-1* | AAGAGCGTAAACCAAGAAAG | AAGAGCGTAAACCAAGAAAG |
| *Bax* | ACTGGACAGCAATATGGAGC | CAAAGTAGAAGAGGGCAACC |
| *Bik* | CTCAGGGAAAACATCTGGTC | TCACTGAAGCTGCAAATACC |
| *Bad* | GAGTCGCCACAGTTCGTA | CTCATCGCTCATCCTTCG |
| *Bak* | GGAATGCCTACGAACTCTTC | GATCCATCTGGCGATGTAAT |
| *Gapdh* | CCCTTAAGAGGGATGCTGCC | TACGGCCAAATCCGTTCACA |
