# Supplementary material for: MicroRNA-325-3p Facilitates Immune Escape of Mycobacterium tuberculosis through Targeting LNX1 via NEK6 Accumulation to Promote Anti-Apoptotic STAT3 Signaling
Source: mBio. 2020 Jun 2;11(3):e00557-20. doi: 10.1128/mBio.00557-20 (PMC7267881; doi:10.1128/mBio.00557-20)
Supplement: FIG S7 [file mBio.00557-20-sf007.pdf]

# Figure 2H

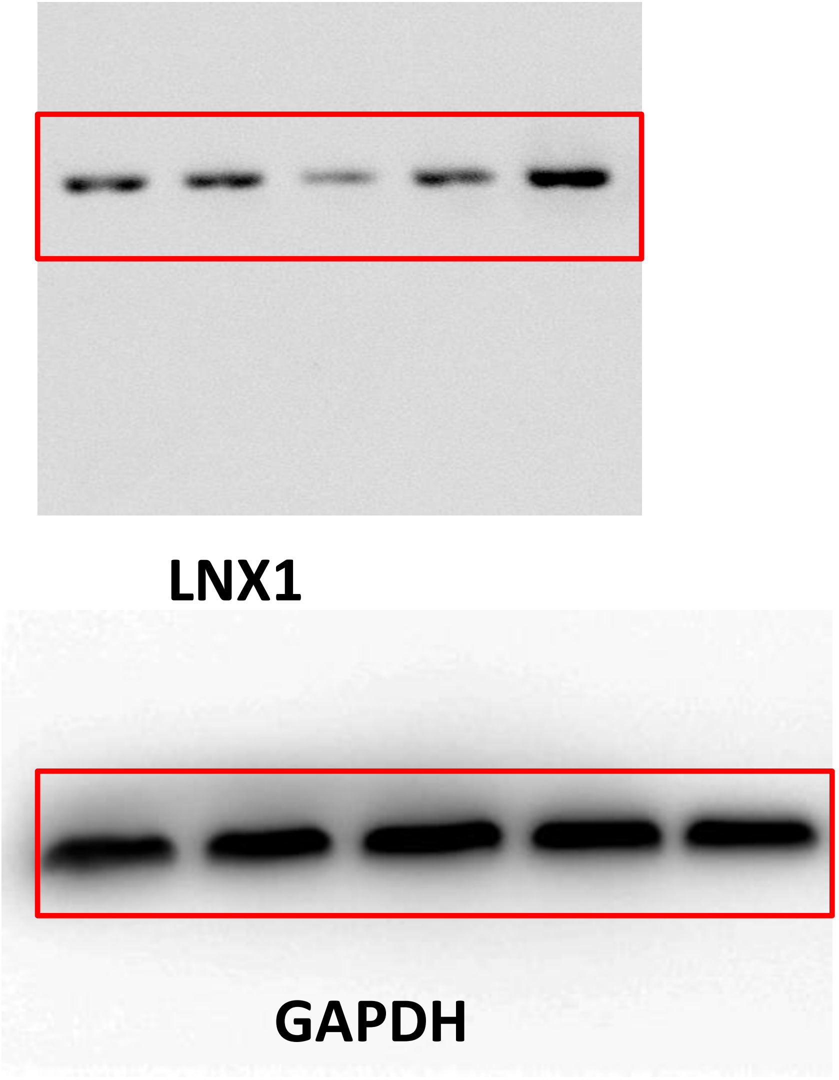

# Figure 2I

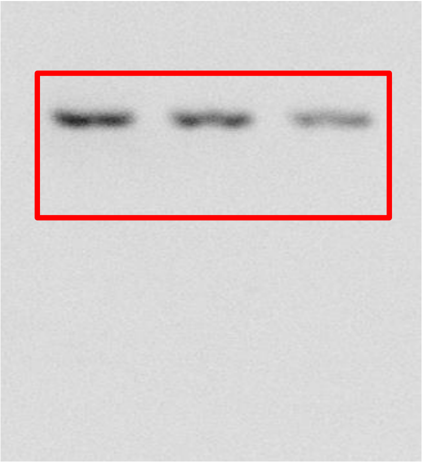

RAW264.7 LNX1

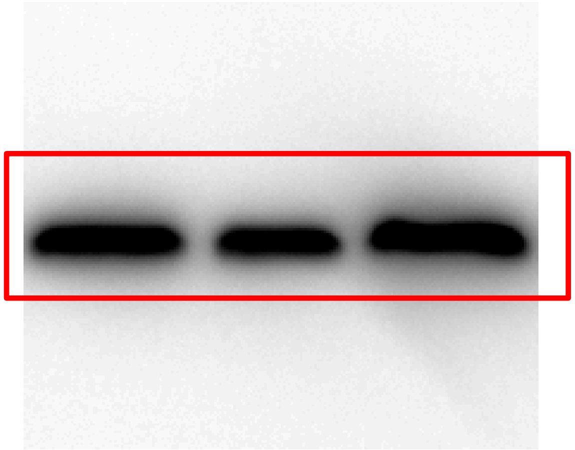

RAW264.7 GAPDH

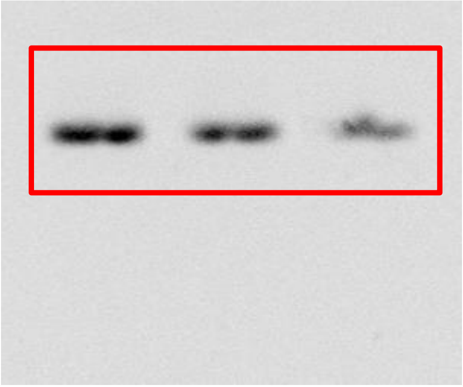

BMDM LNX1

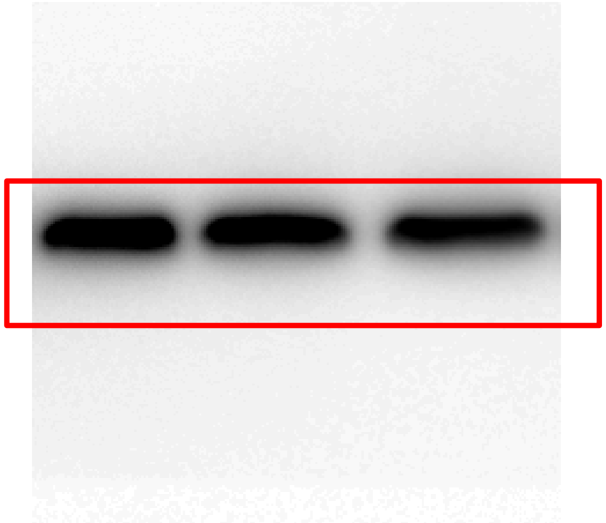

BMDM GAPDH

# Figure 2J

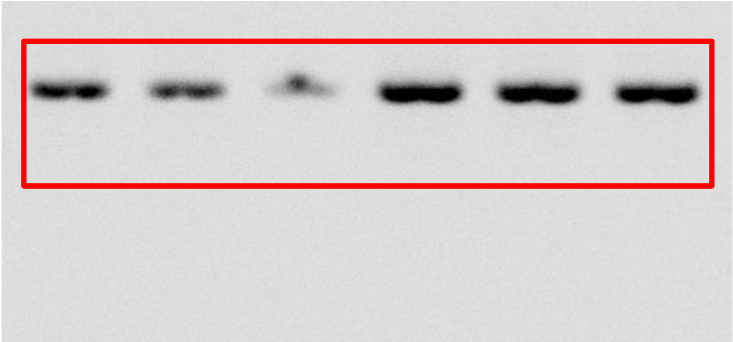

LNX1

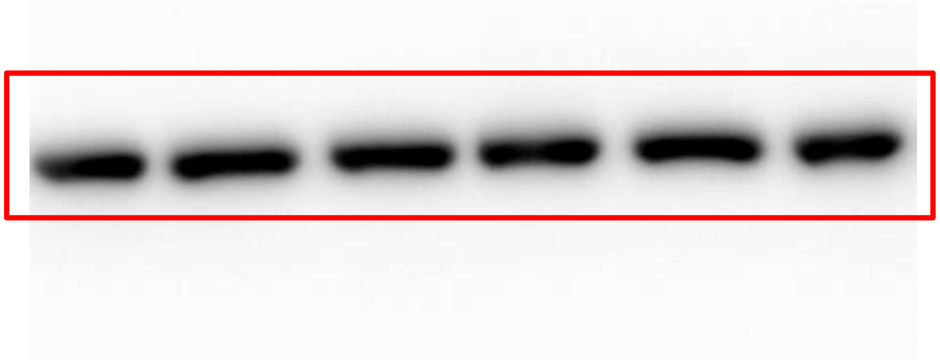

GAPDH

# Figure 2K

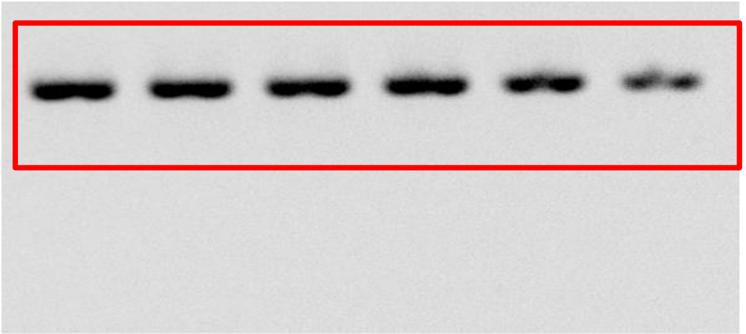

LNX1

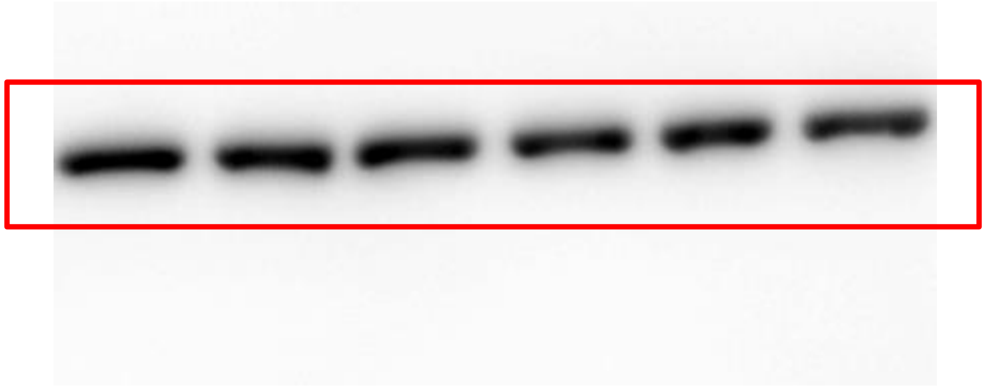

GAPDH

# Figure 3A

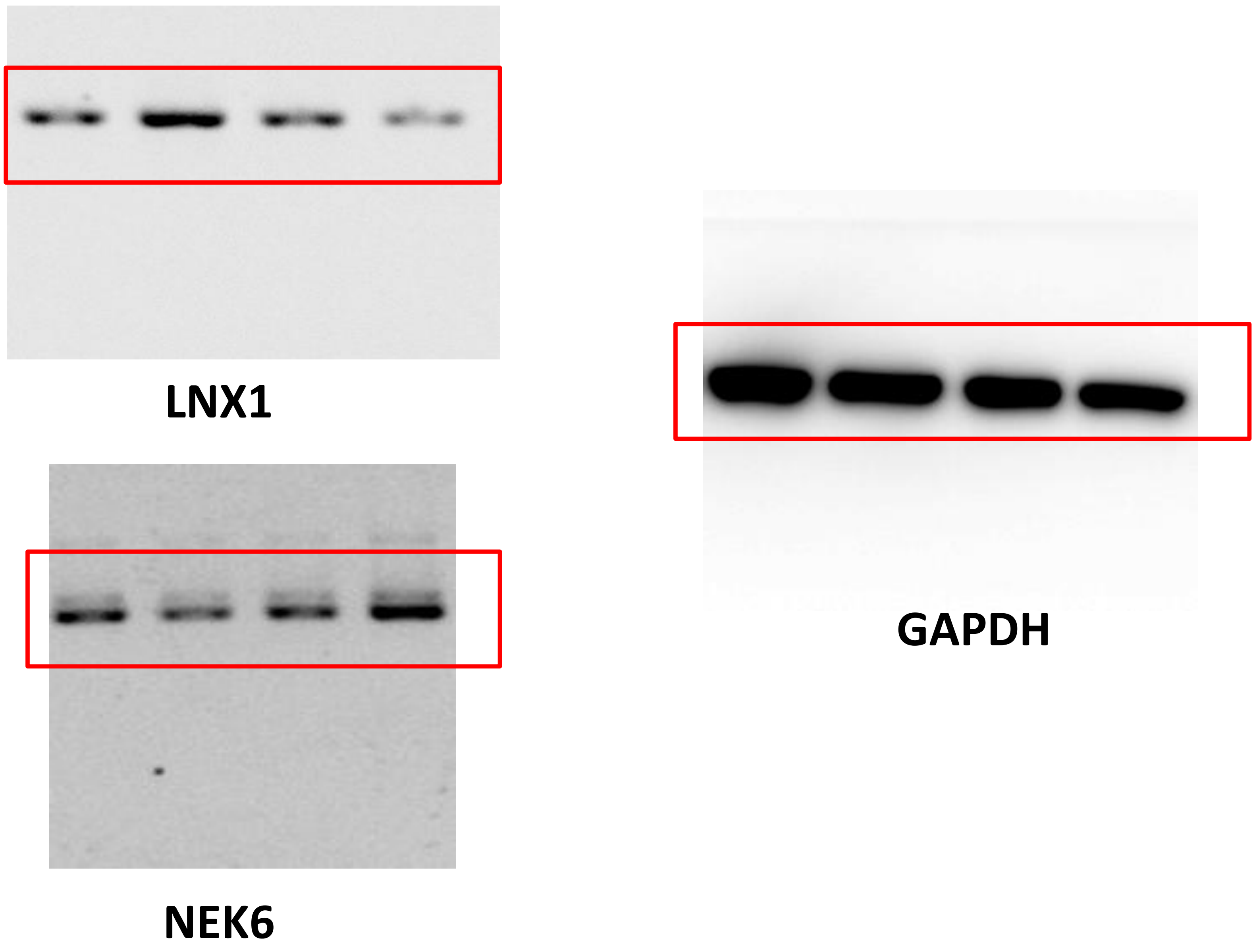

# Figure 3B

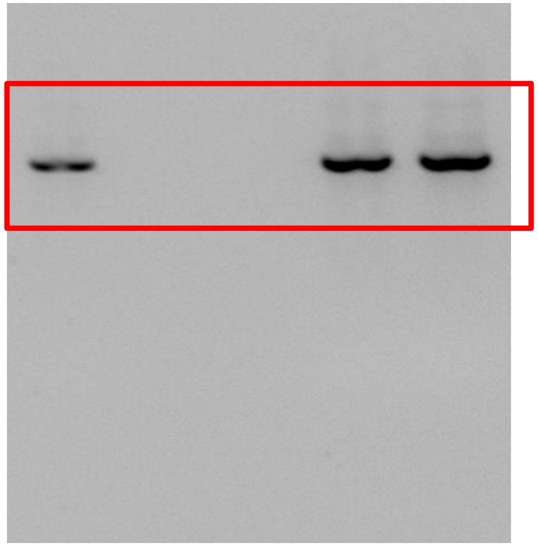

LNX1

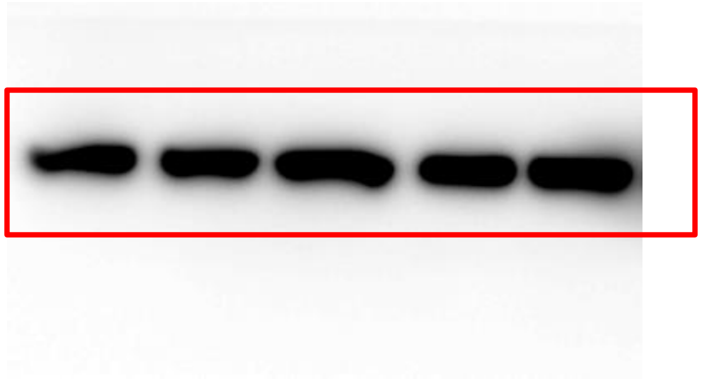

GAPDH

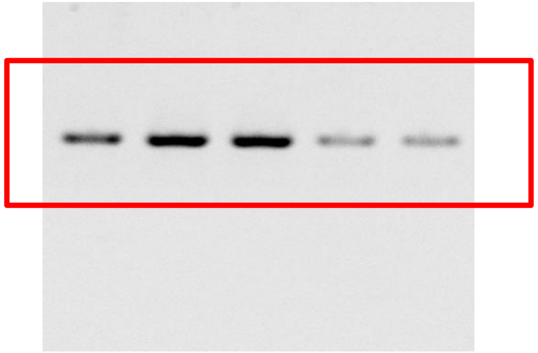

NEK6

**Figure 3C**

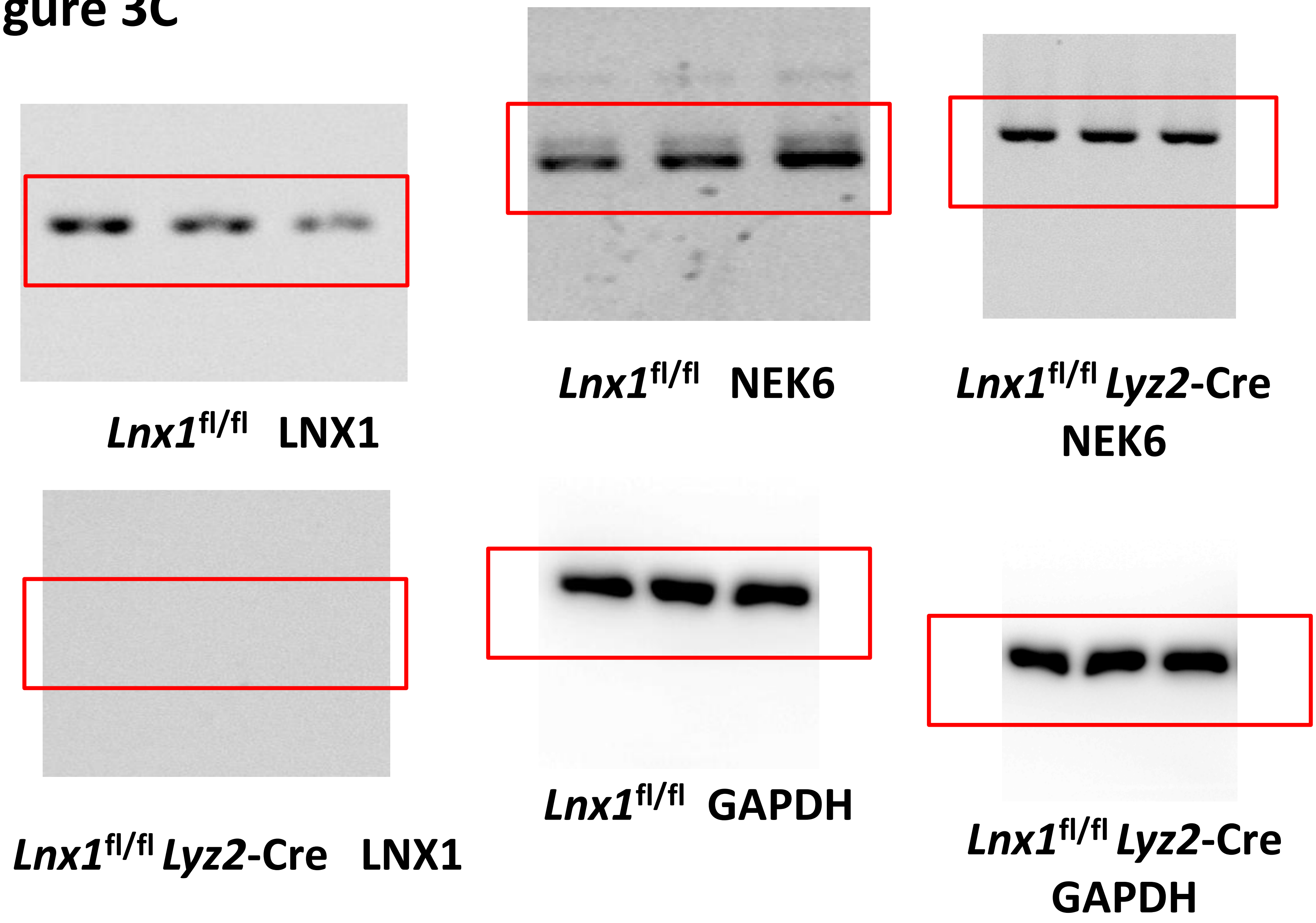

*Lnx1*fl/fl LNX1
*Lnx1*fl/fl NEK6
*Lnx1*fl/fl *Lyz2*-Cre NEK6
*Lnx1*fl/fl *Lyz2*-Cre LNX1
*Lnx1*fl/fl GAPDH
*Lnx1*fl/fl *Lyz2*-Cre GAPDH

# Figure 3D

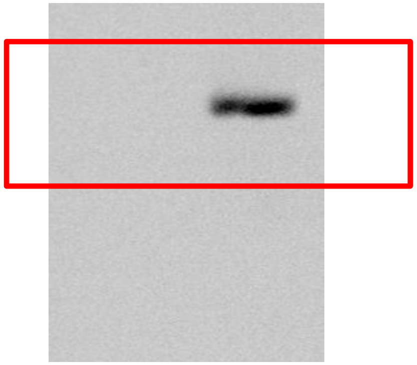

IP:Myc IB:HA

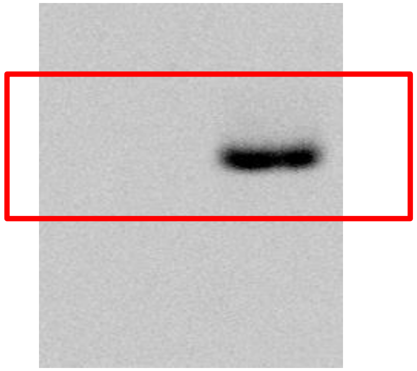

WCL IB:Myc

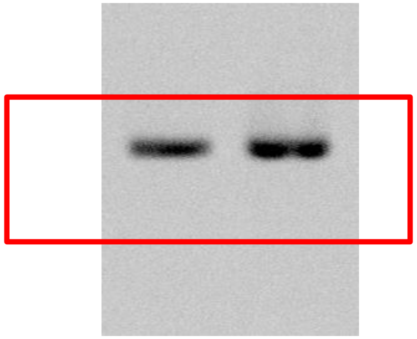

WCL IB:HA

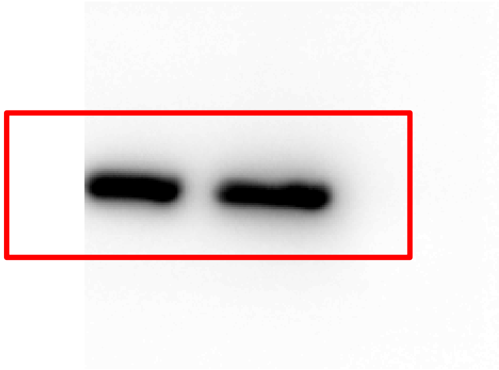

WCL IB:GAPDH

# Figure 3E

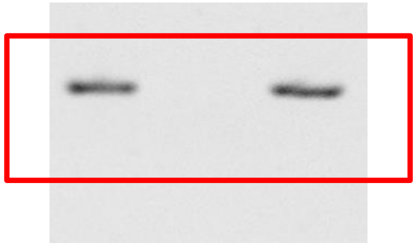

Myc

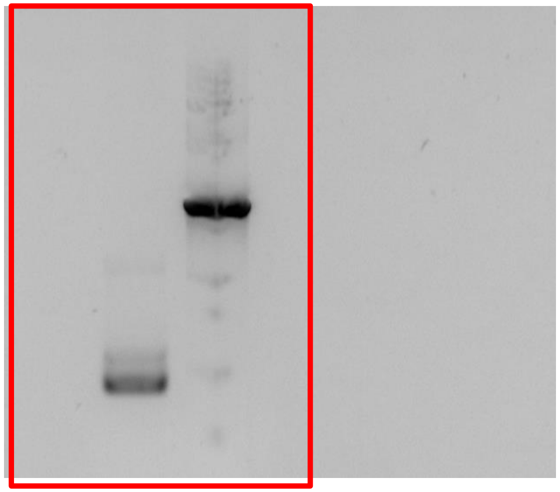

GST

# Figure 3F

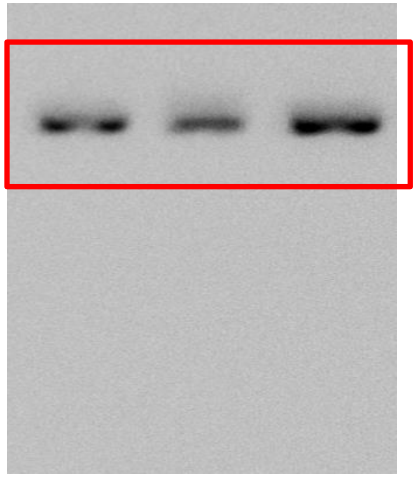

IP:NEK6 IB:LNX1

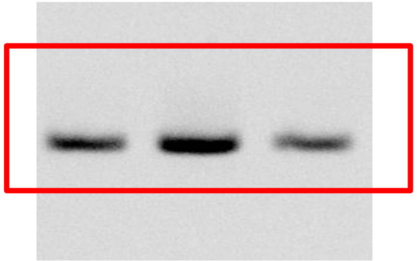

WCL IB:NEK6

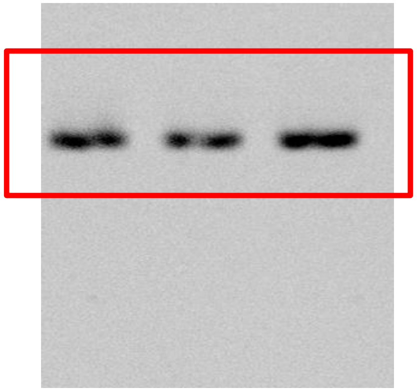

WCL IB:LNX1

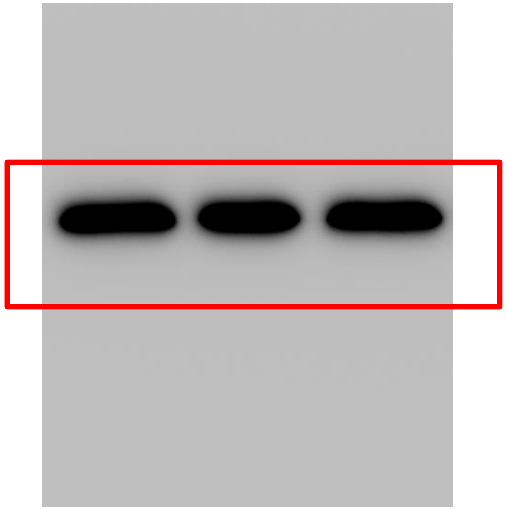

WCL IB:GAPDH

# Figure 3G

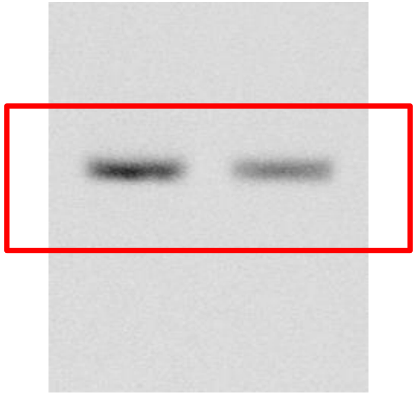

IP:NEK6 IB:LNX1

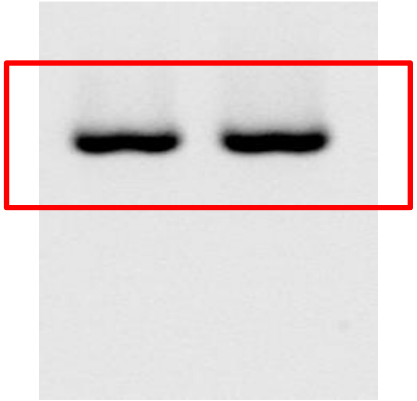

WCL IB:NEK6

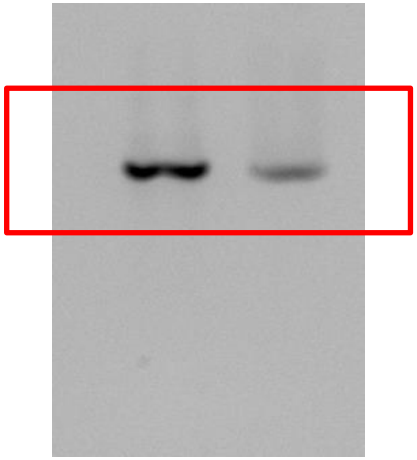

WCL IB:LNX1

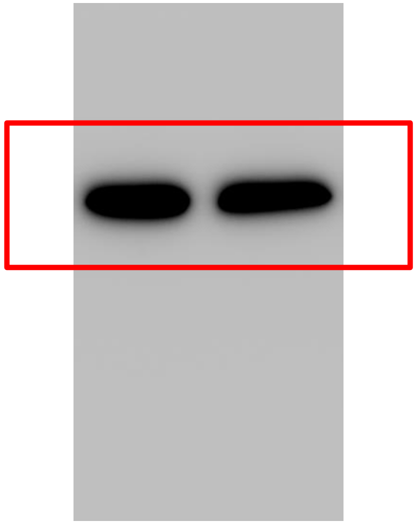

WCL IB:GAPDH

# Figure 3H

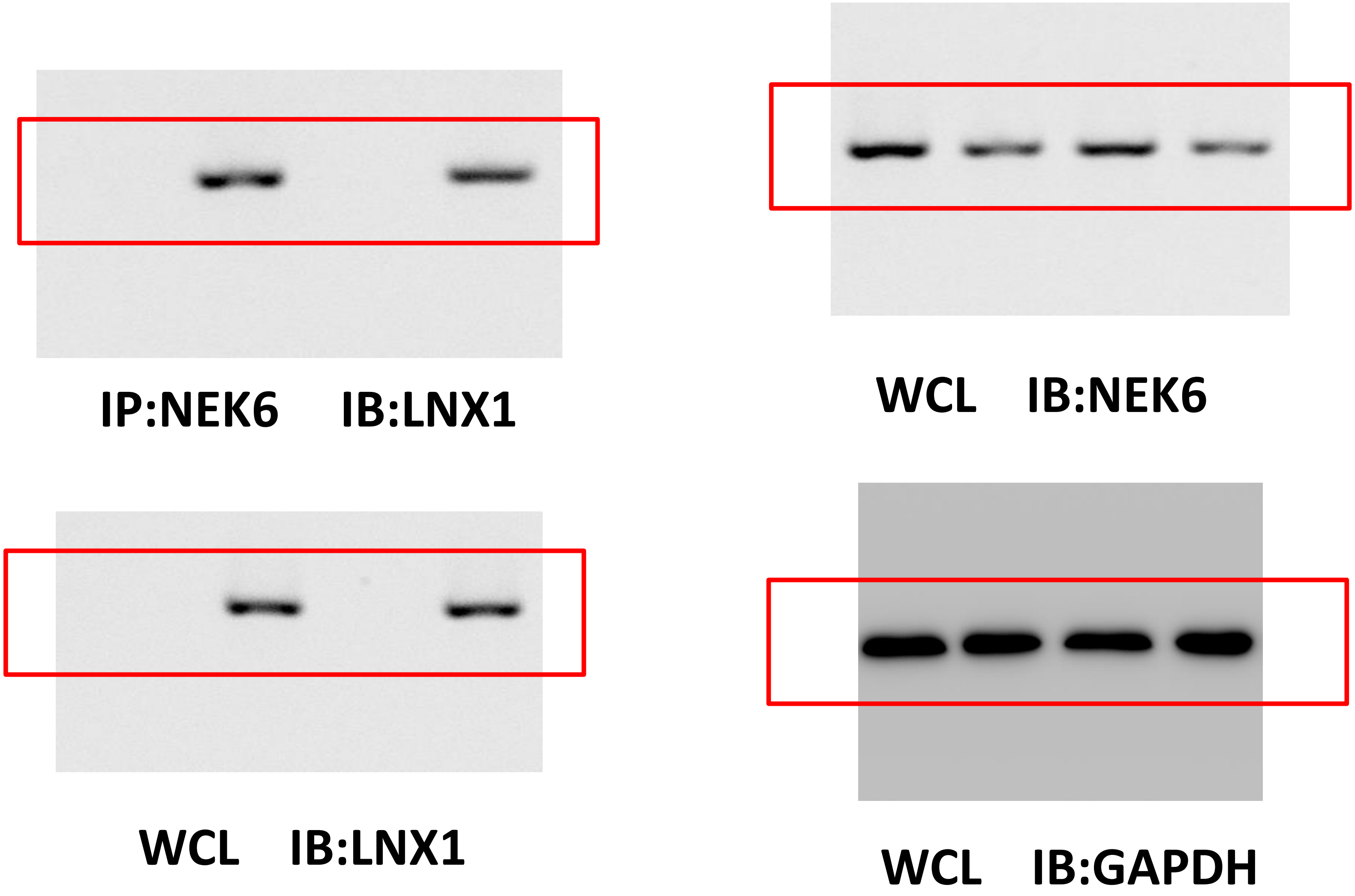

# Figure 3I

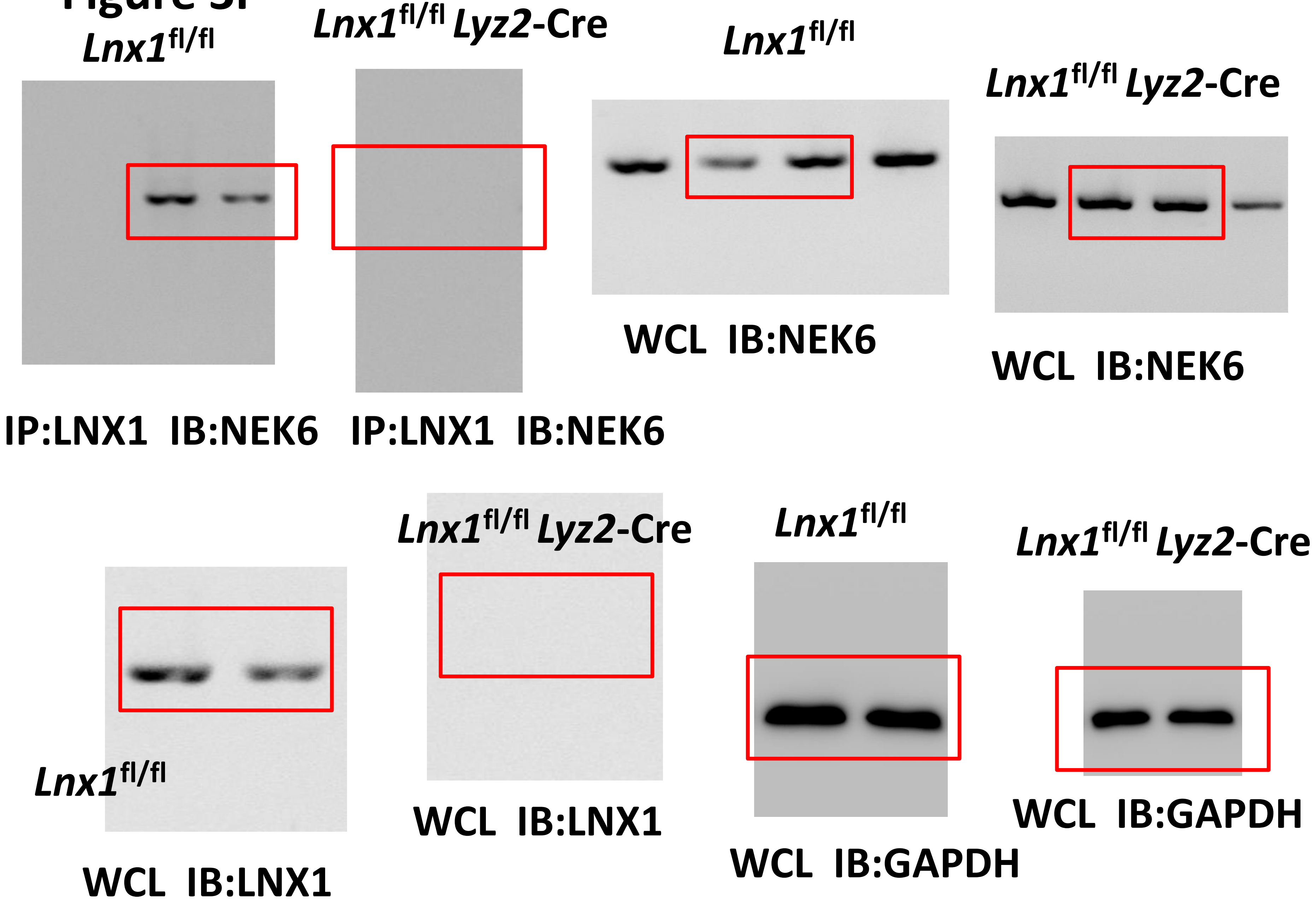

Lnx1fl/fl
Lnx1fl/fl Lyz2-Cre
Lnx1fl/fl
Lnx1fl/fl Lyz2-Cre
WCL IB:NEK6
WCL IB:NEK6
IP:LNX1 IB:NEK6
IP:LNX1 IB:NEK6
Lnx1fl/fl Lyz2-Cre
Lnx1fl/fl
Lnx1fl/fl Lyz2-Cre
Lnx1fl/fl
WCL IB:LNX1
WCL IB:GAPDH
WCL IB:GAPDH
WCL IB:LNX1

# Figure 3J

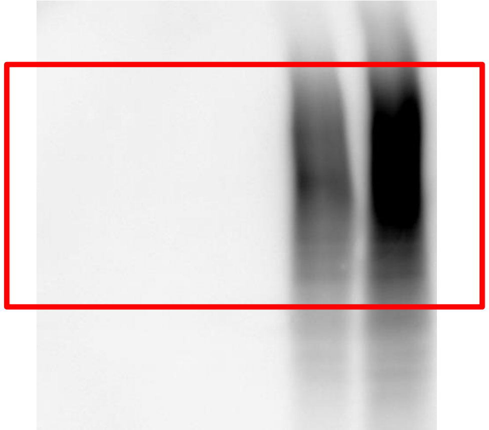

IP:Myc IB:HA

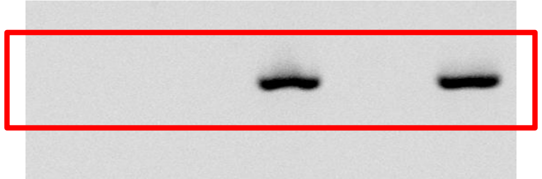

WCL IB:Flag

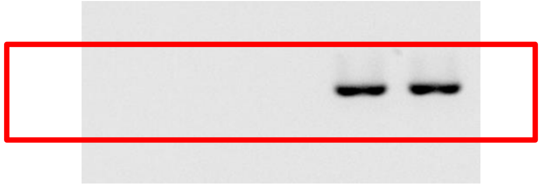

WCL IB:Myc

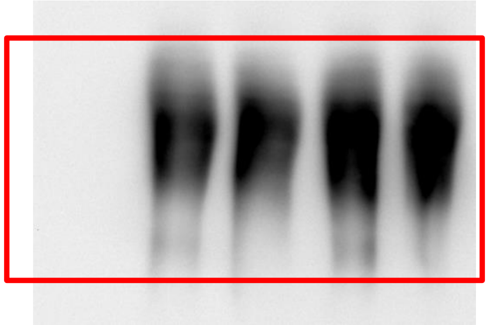

WCL IB:HA

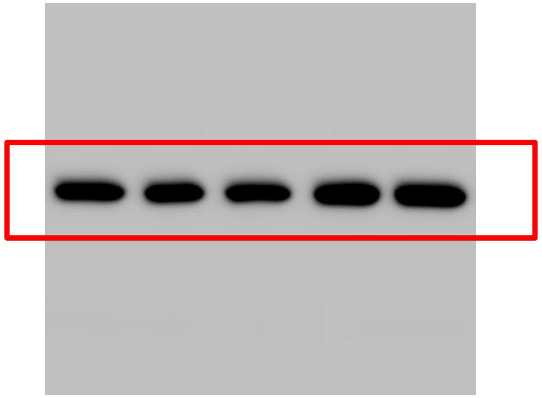

WCL IB:GAPDH

# Figure 3K

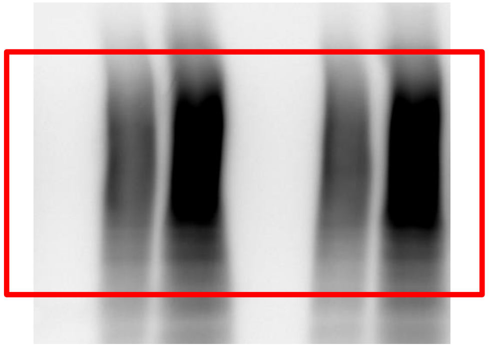

IP:NEK6 IB:HA

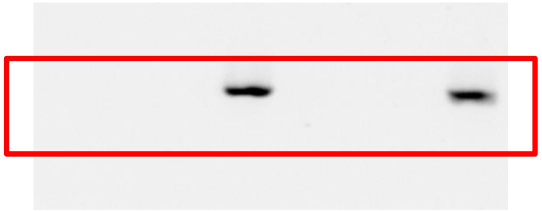

WCL IB:LNX1

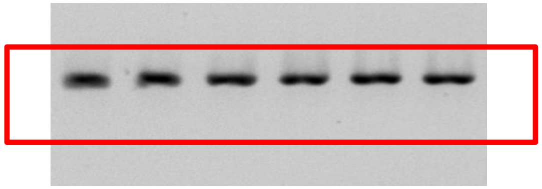

WCL IB:NEK6

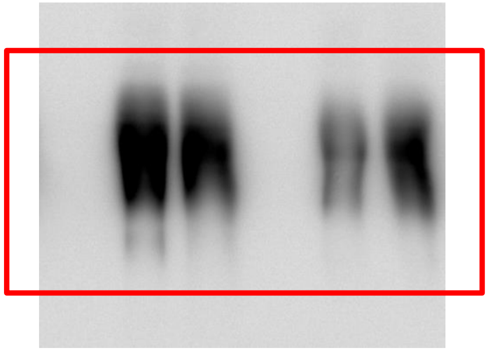

WCL IB:HA

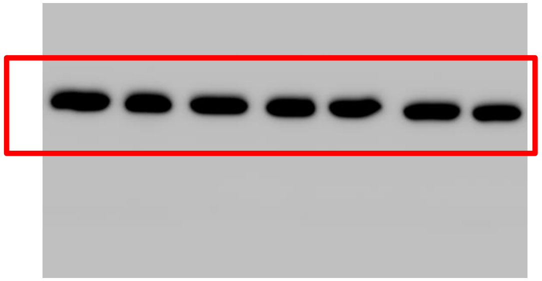

WCL IB:GAPDH

# Figure 3L

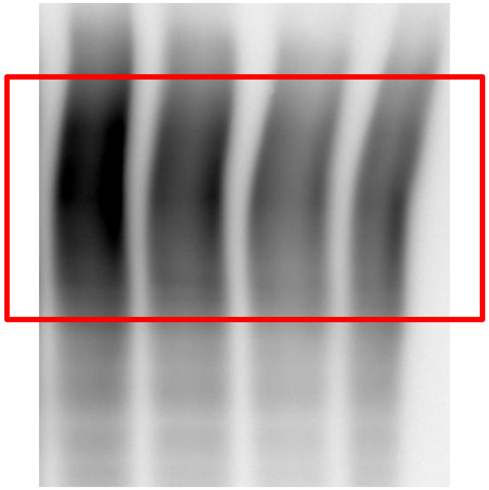

IP:NEK6 IB:Ub

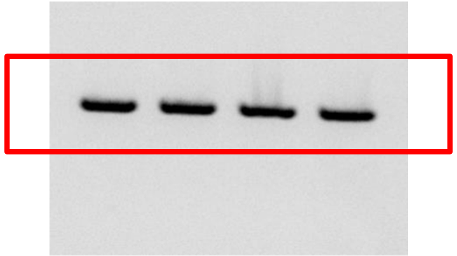

WCL IB:NEK6

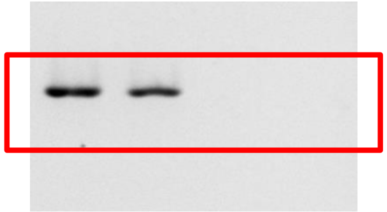

WCL IB:LNX1

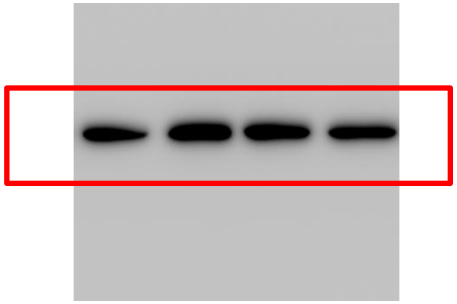

WCL IB:GAPDH

Figure 4B

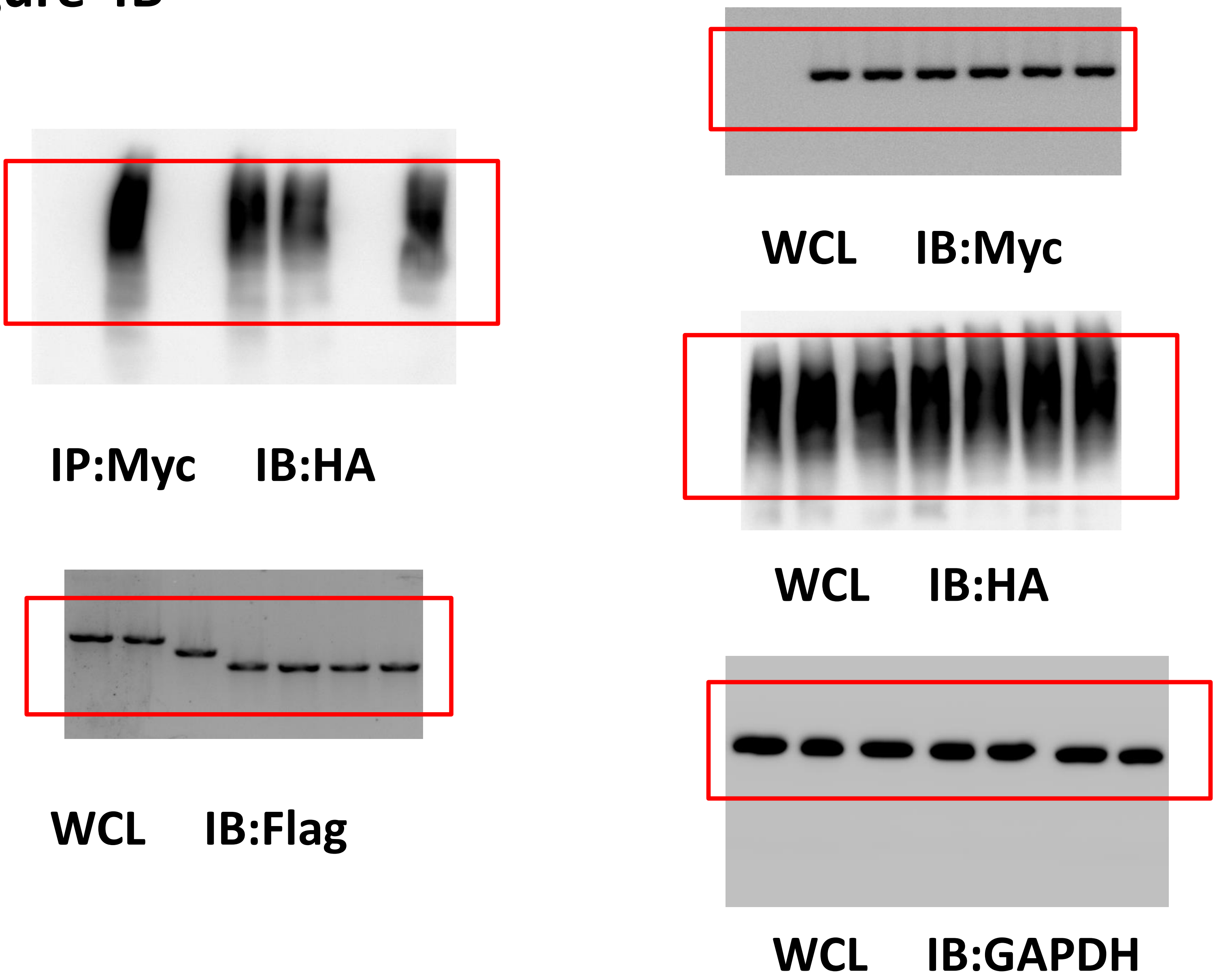

# Figure 4C

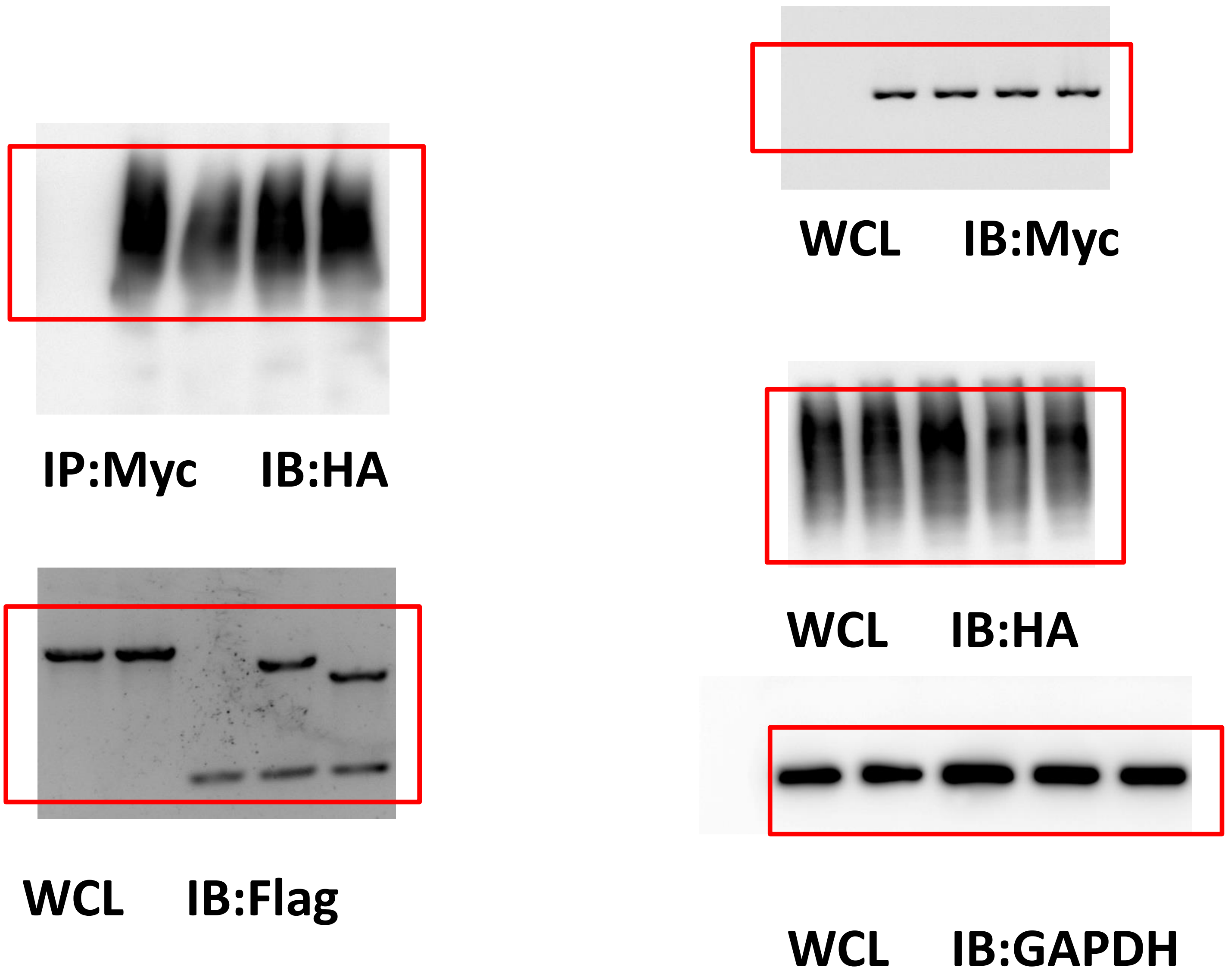

# Figure 4E

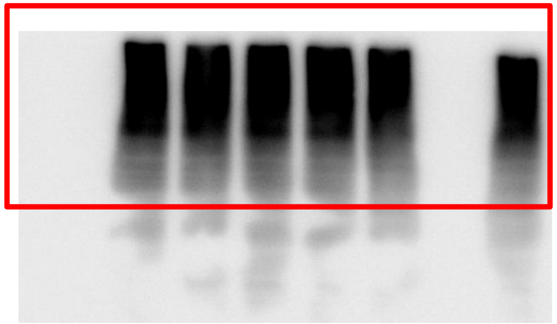

IP:Myc IB:HA

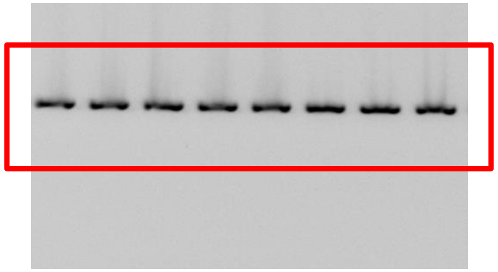

WCL IB:Myc

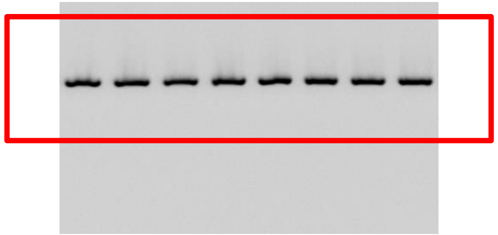

WCL IB:Flag

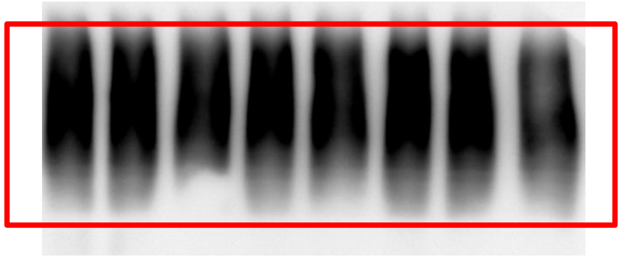

WCL IB:HA

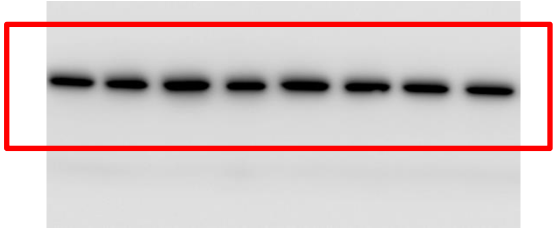

WCL IB:GAPDH

Figure 4F

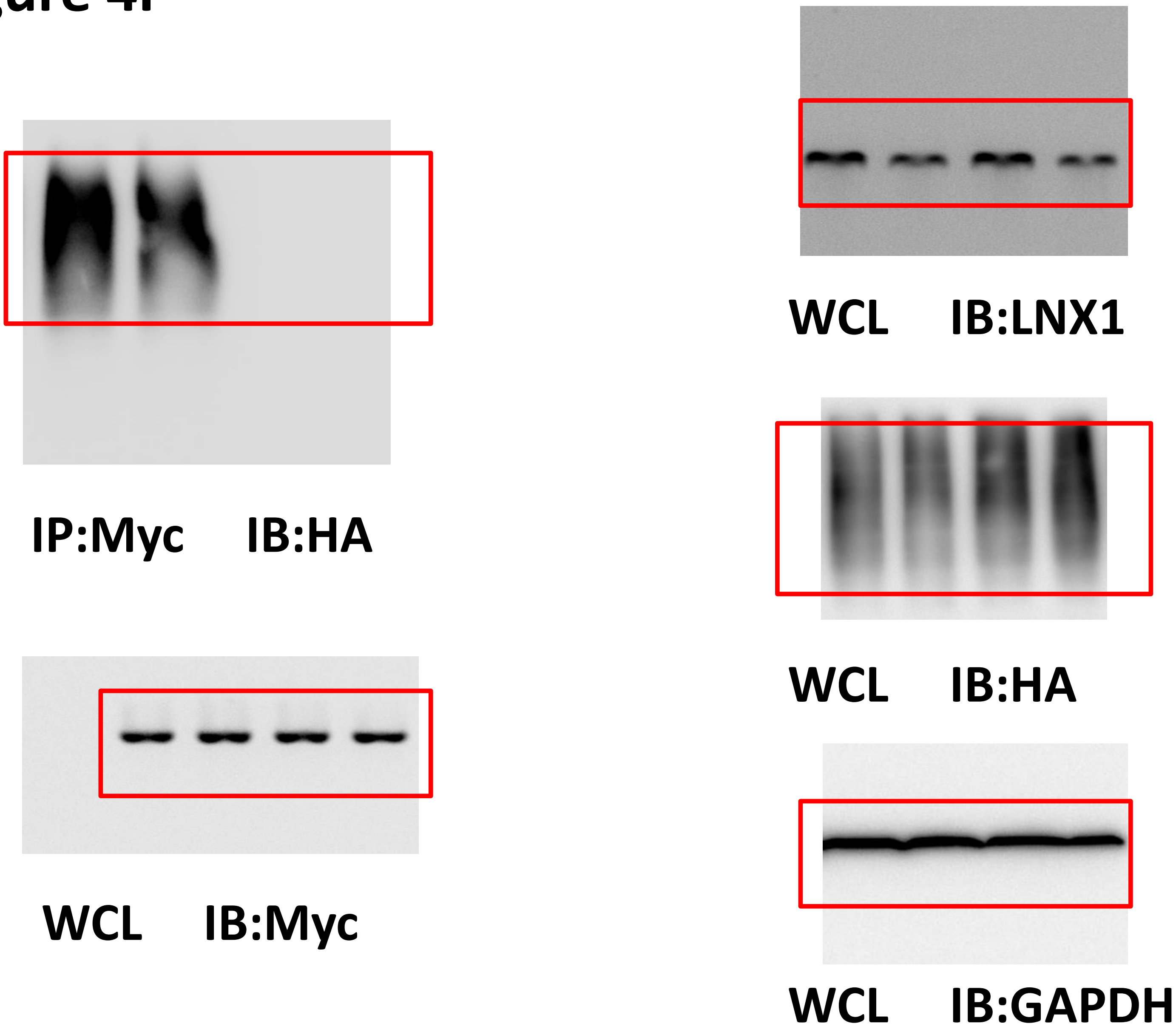

## Figure 4H

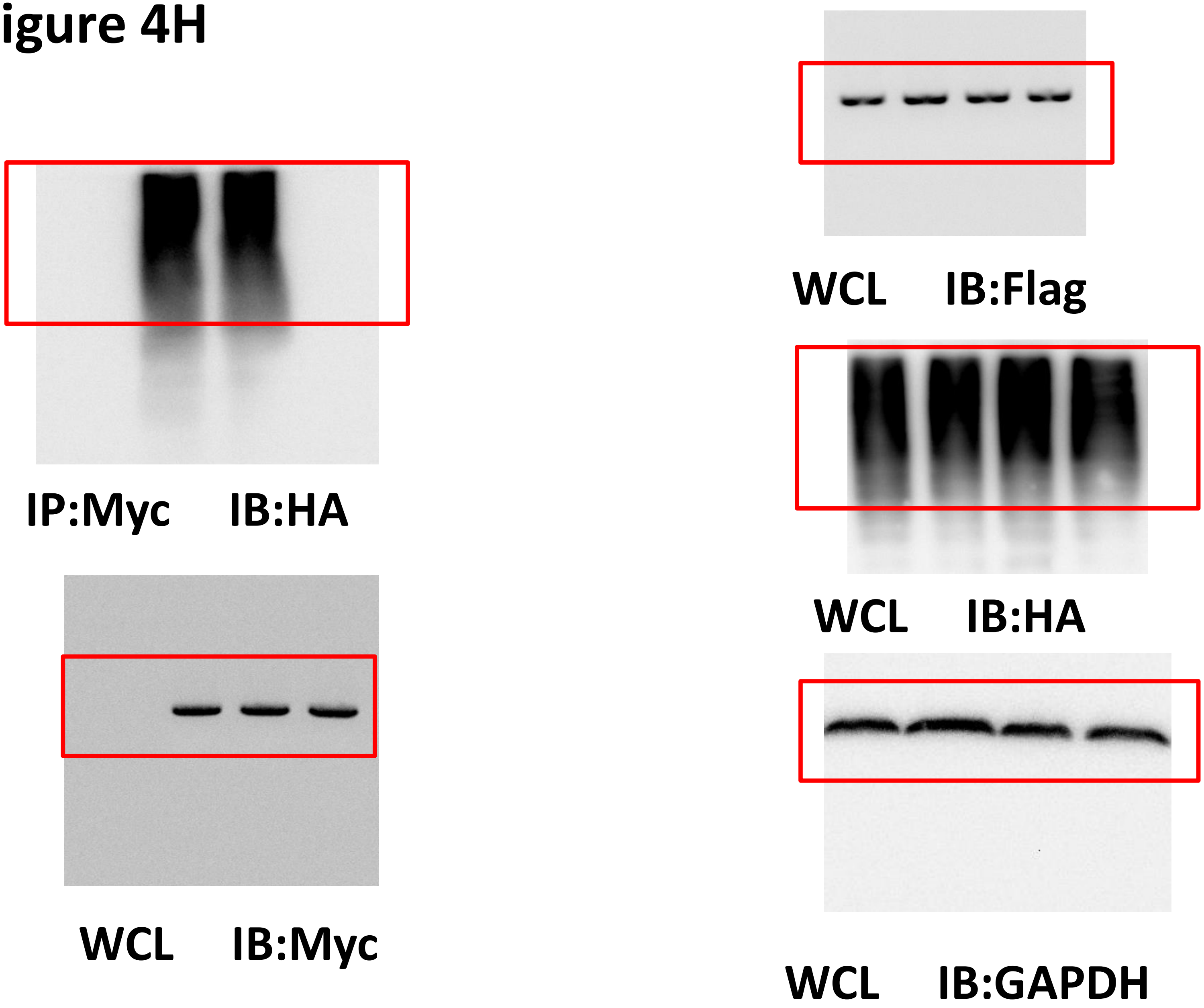

# Figure 5A

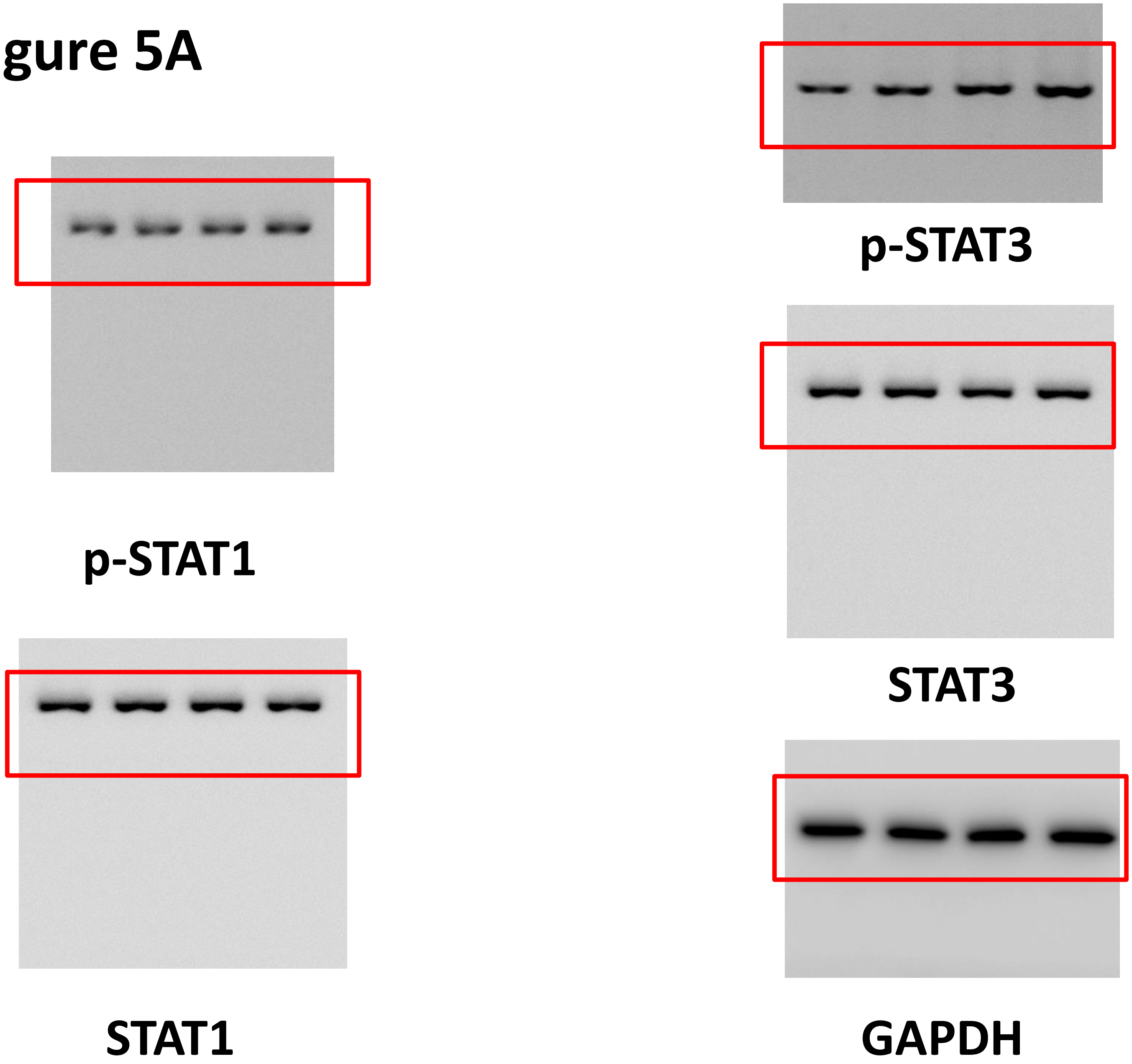

## Figure 5B

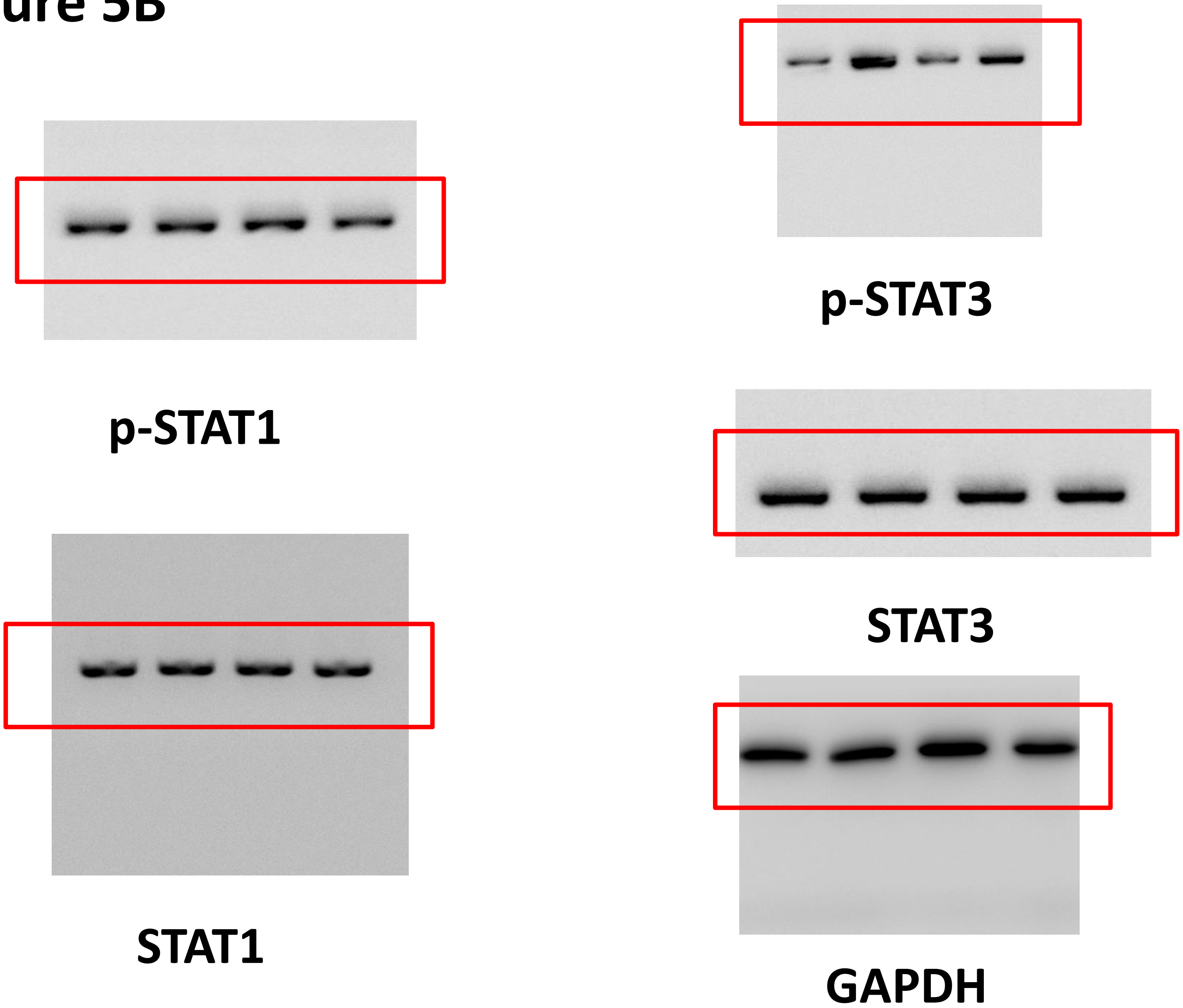

Figure 5C

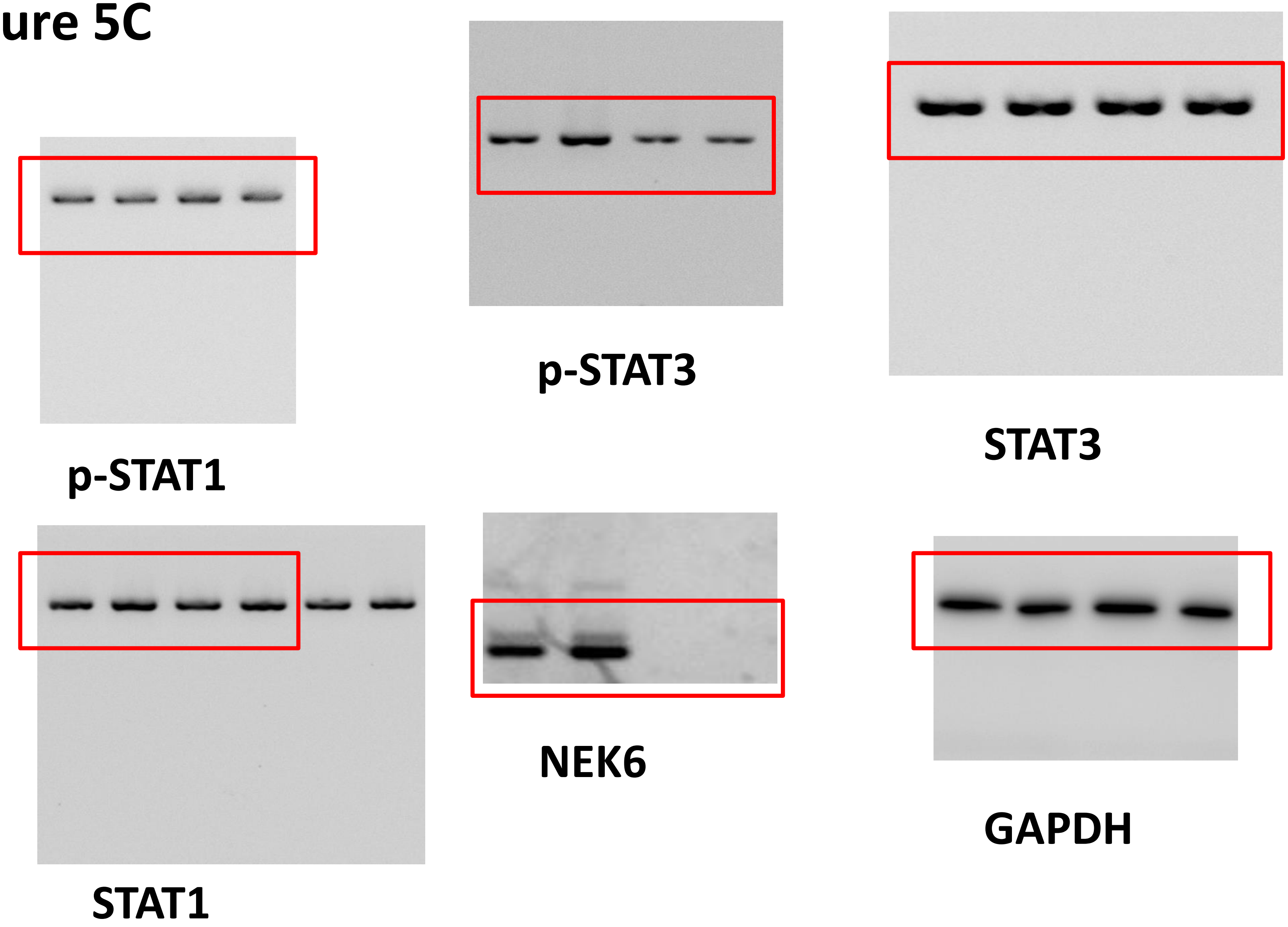

# Figure 5D

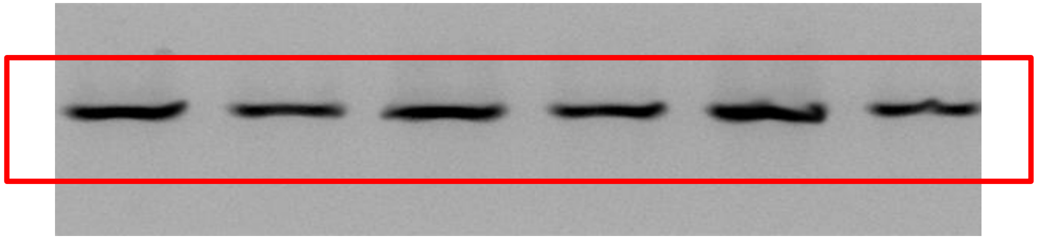

p-STAT3

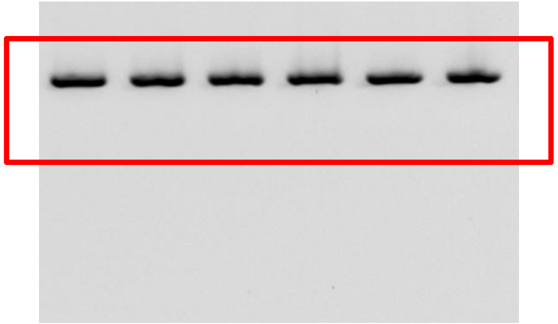

STAT3

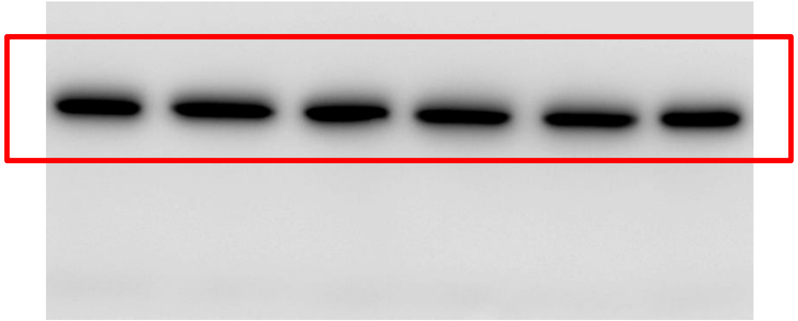

GAPDH

## Figure 5G

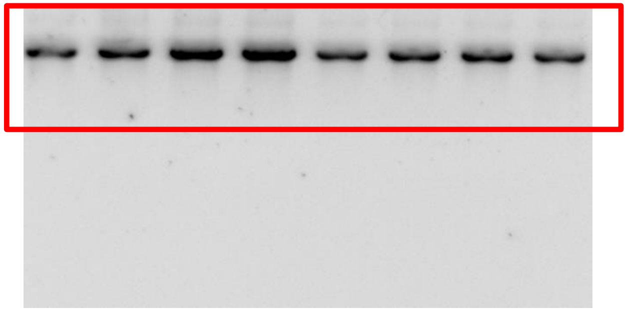

**BCL-2**

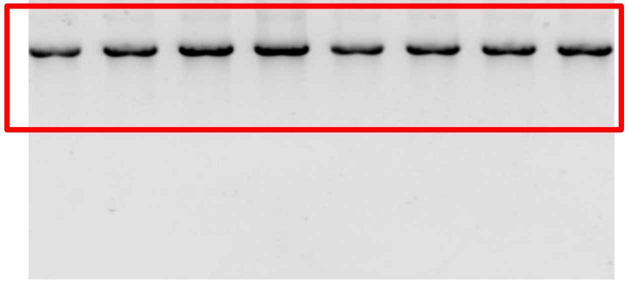

**BCL-$X_L$**

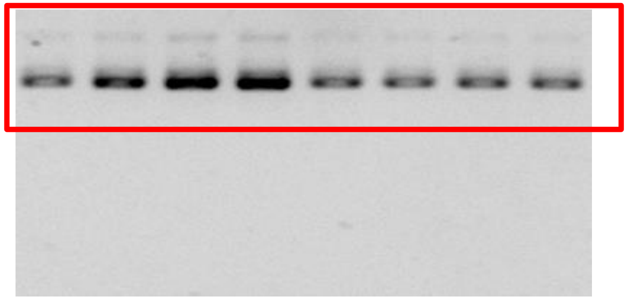

**BCL-W**

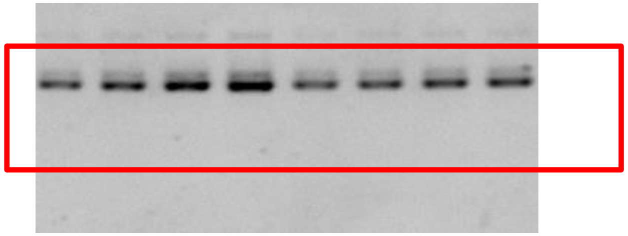

**MCL-1**

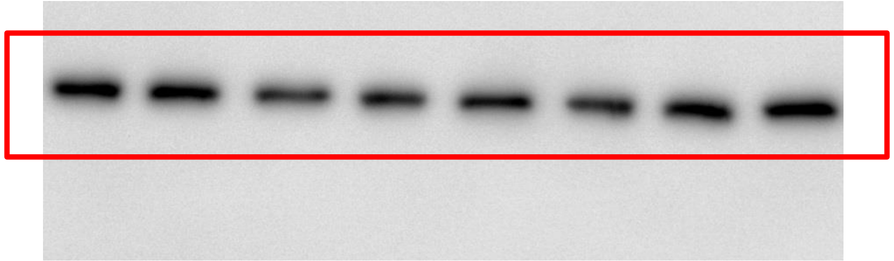

**GAPDH**

Figure 5H

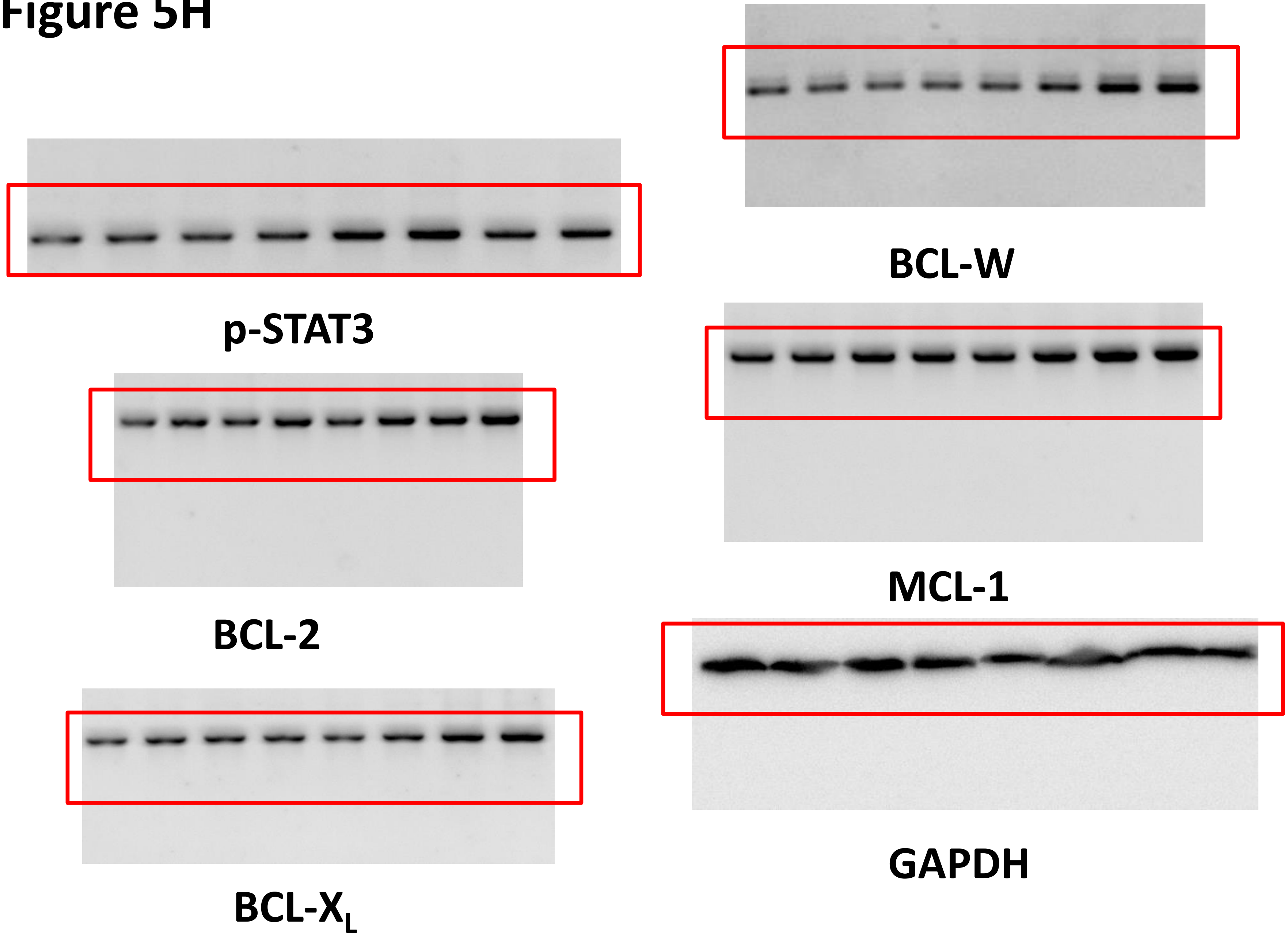

# Figure S3A

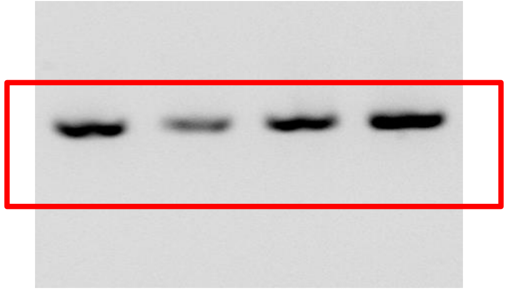

LNX1

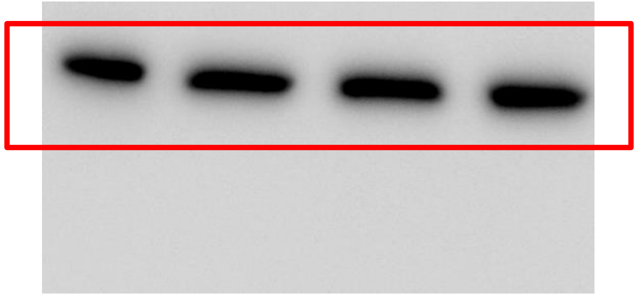

NEK6

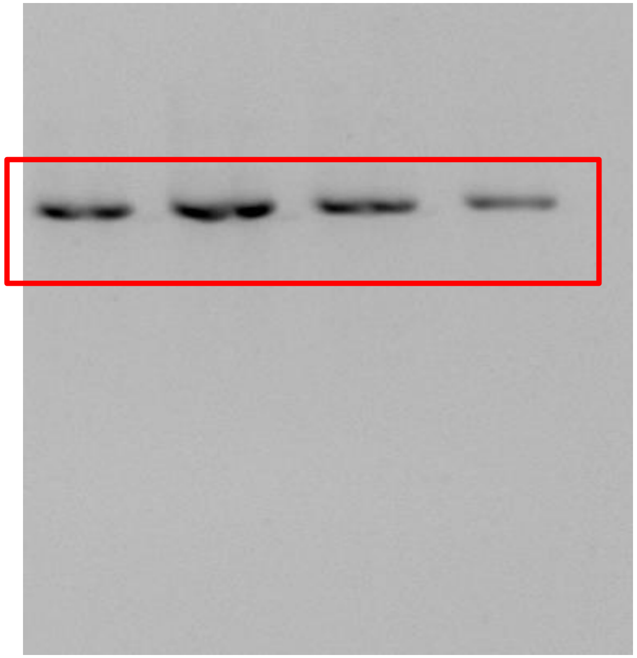

GAPDH

# Figure S3B

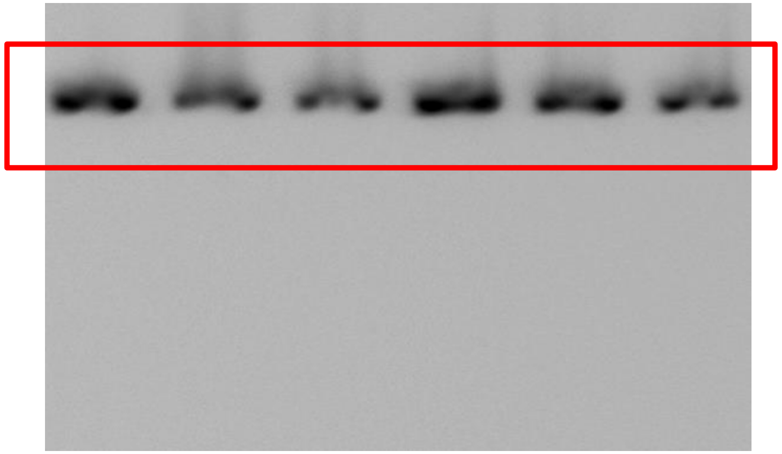

LNX1

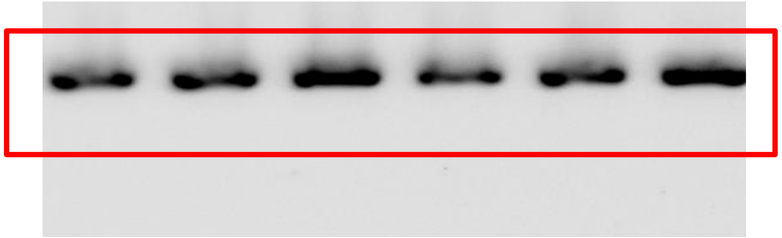

NEK6

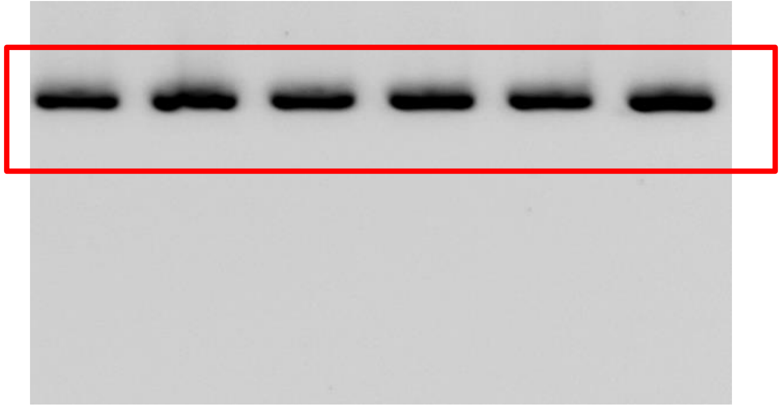

GAPDH

# Figure S3C

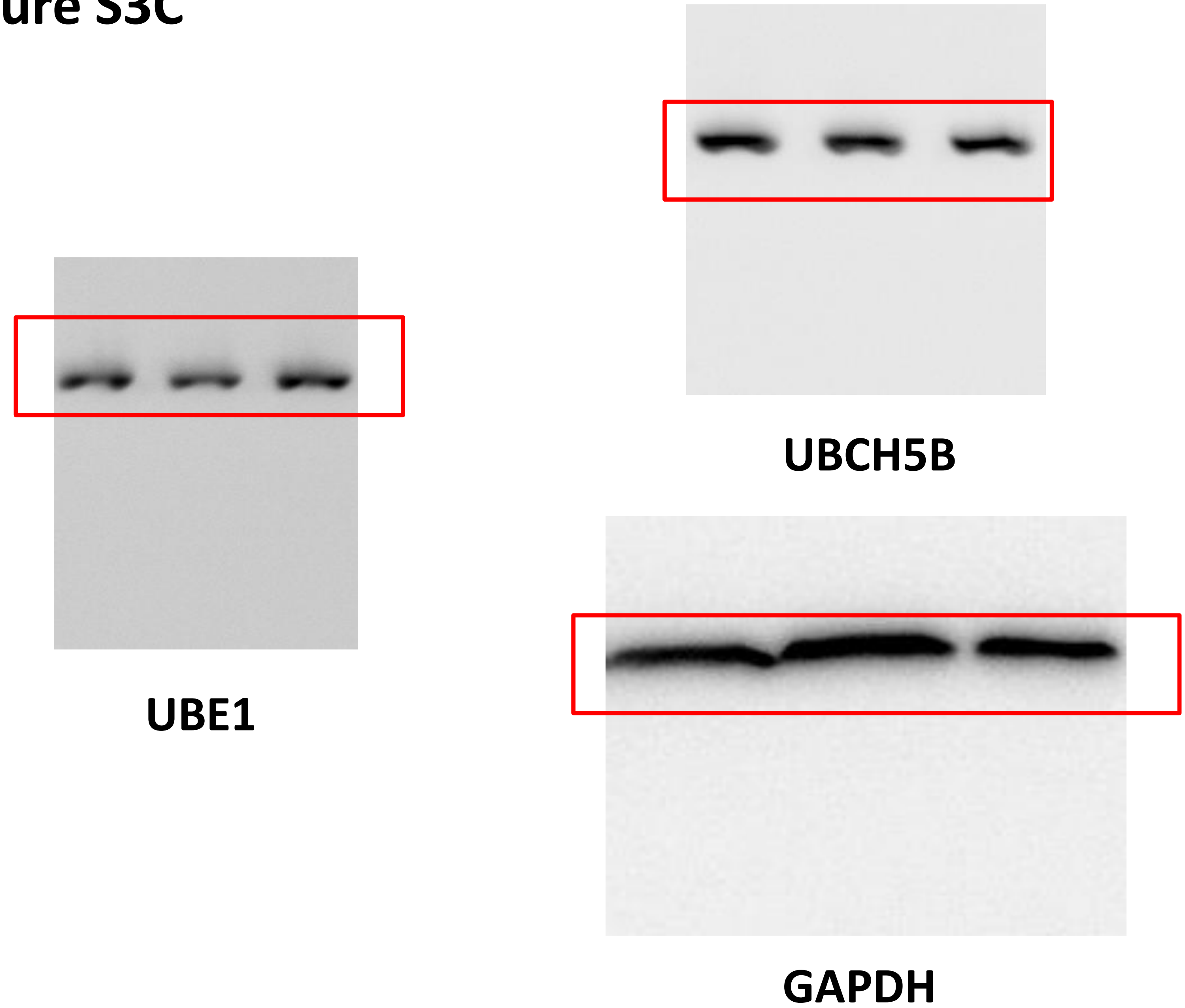

# Figure S3D

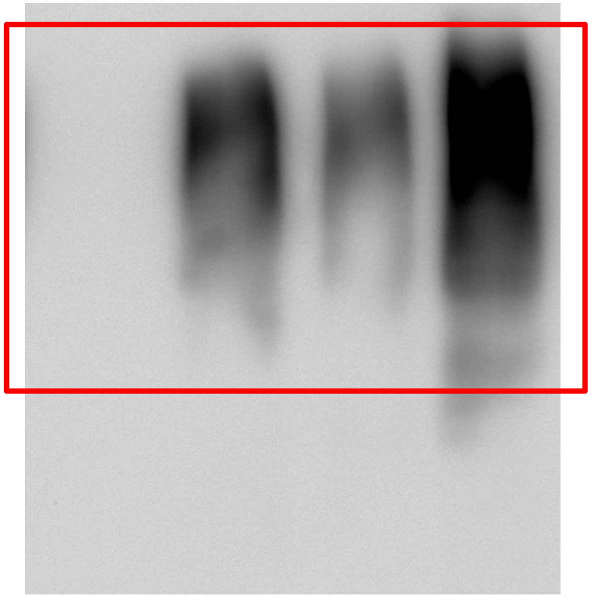

IP:NEK6 IB:HA

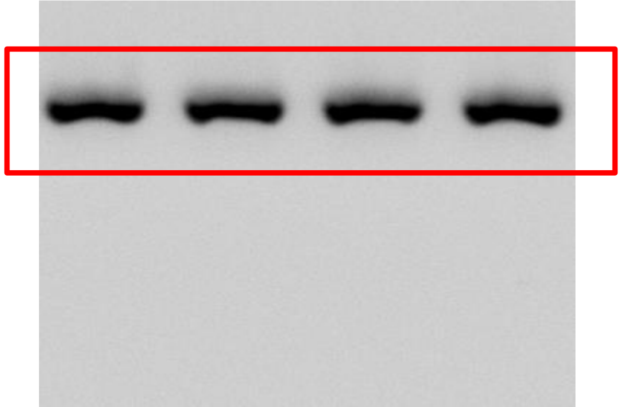

WCL IB:NEK6

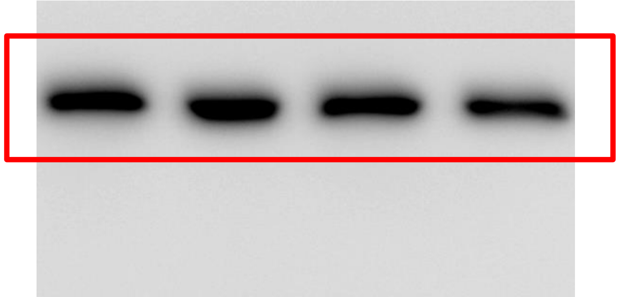

WCL IB:GAPDH

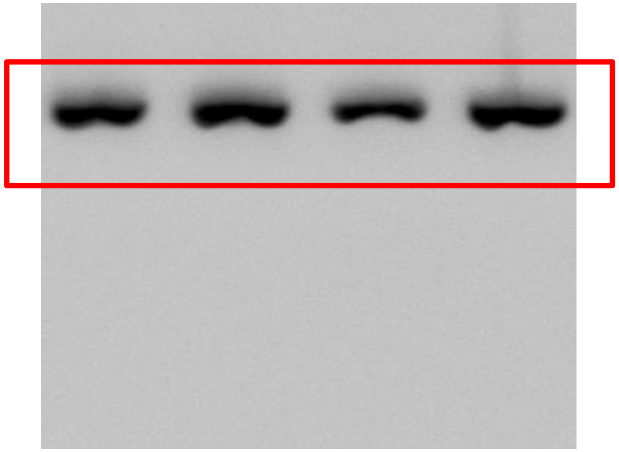

WCL IB:LNX1

# Figure S3E

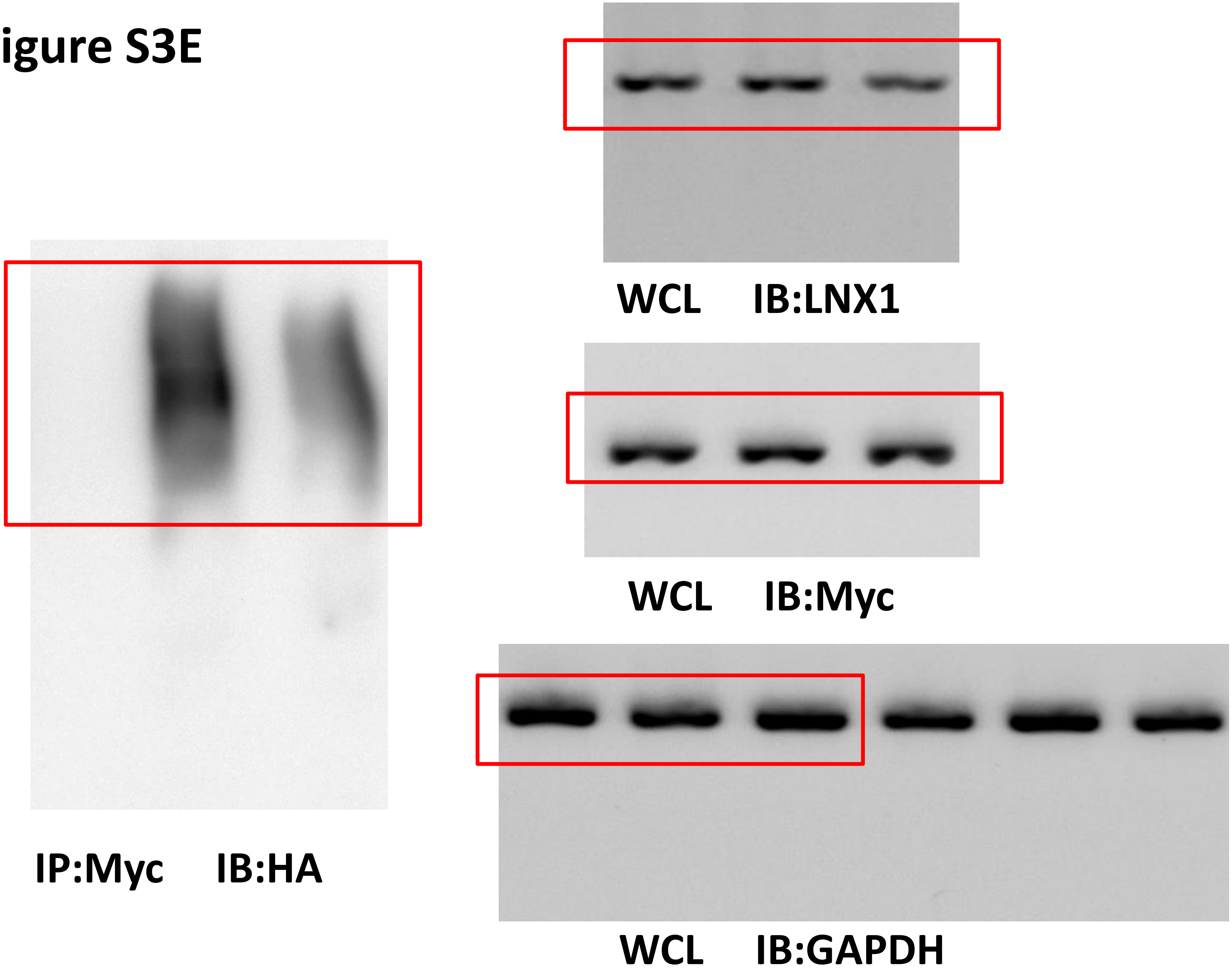

# Figure S3F

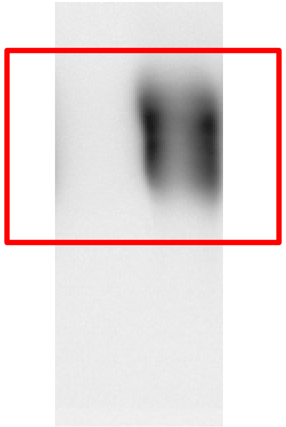

Ub

## Figure S4A

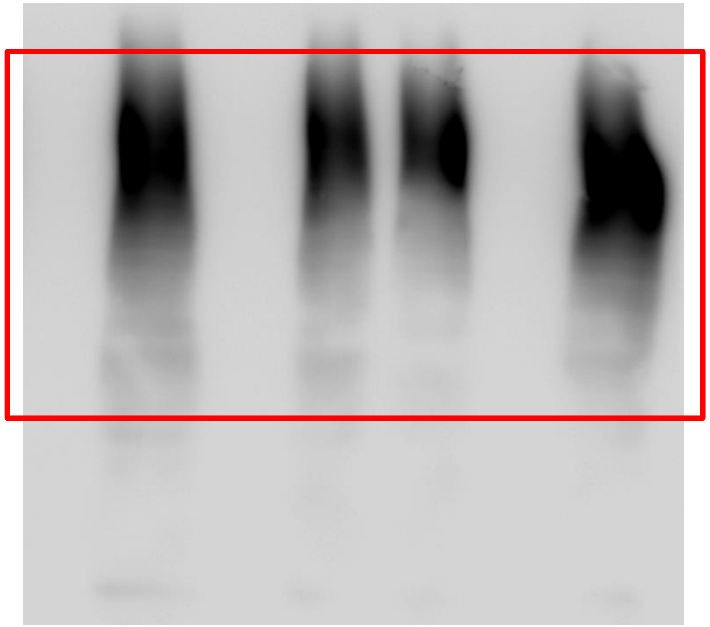

IP:Myc IB:HA

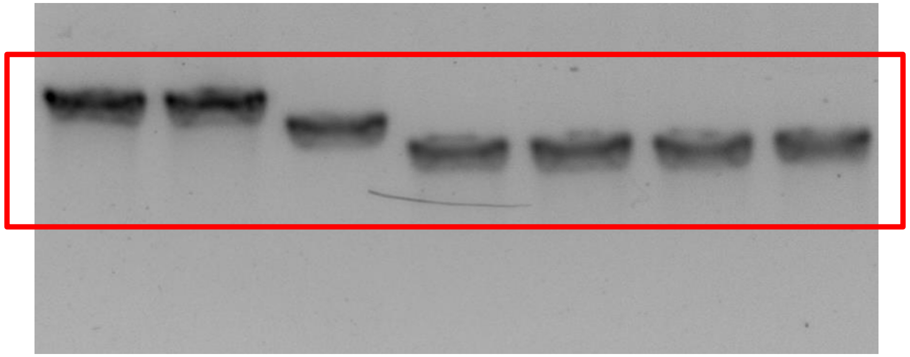

WCL IB:Flag

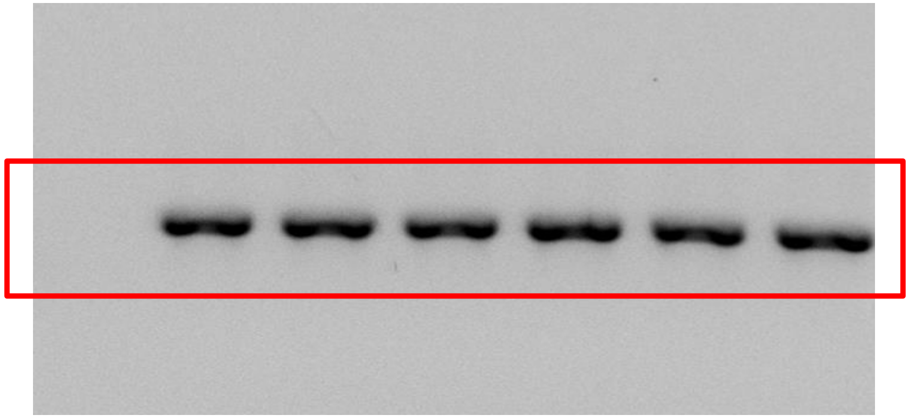

WCL IB:Myc

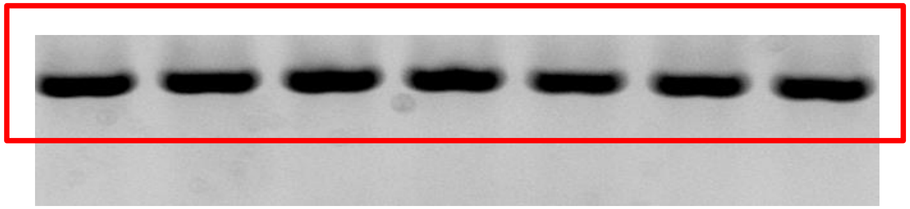

WCL IB:GAPDH

# Figure S4B

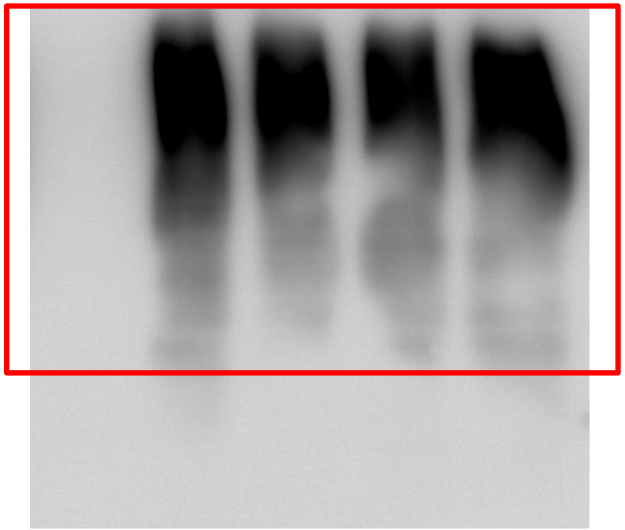

IP:Myc IB:HA

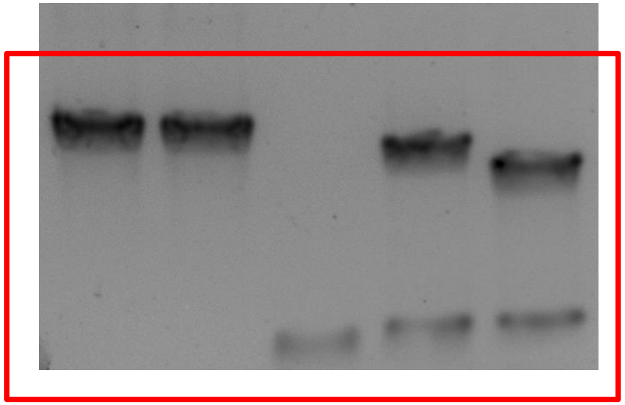

WCL IB:Flag

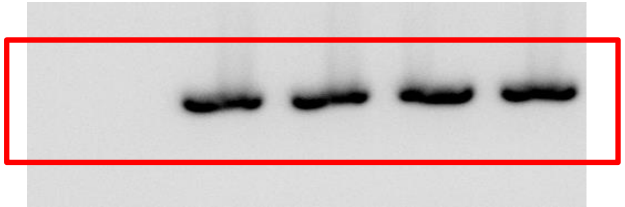

WCL IB:Myc

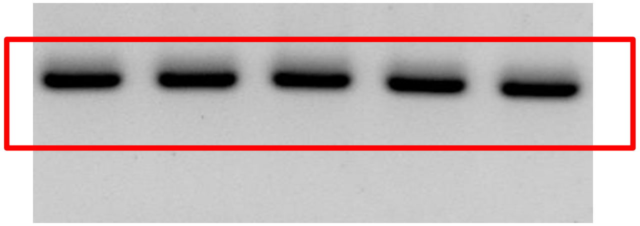

WCL IB:GAPDH

# Figure S4C

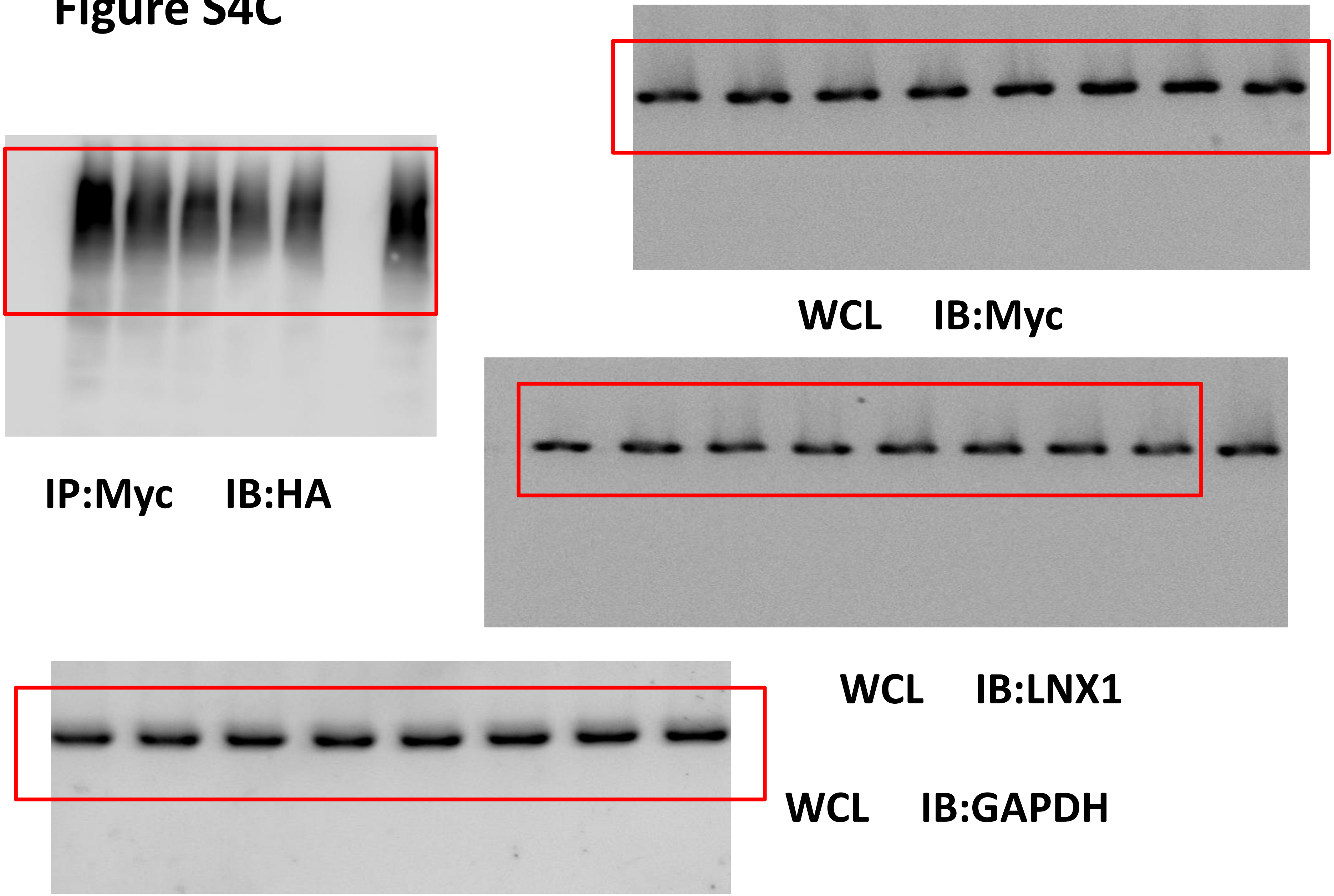

# Figure S4D

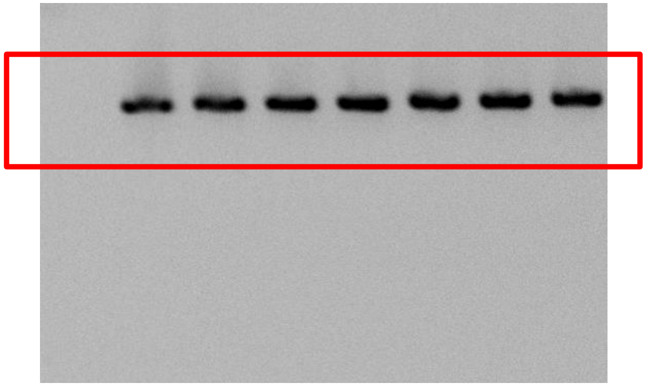

WCL IB:Myc

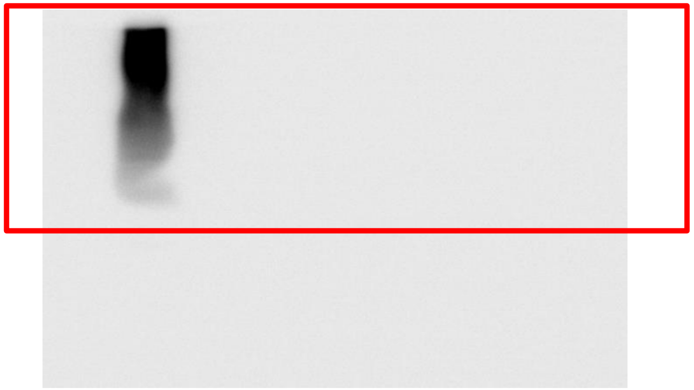

IP:Myc IB:HA

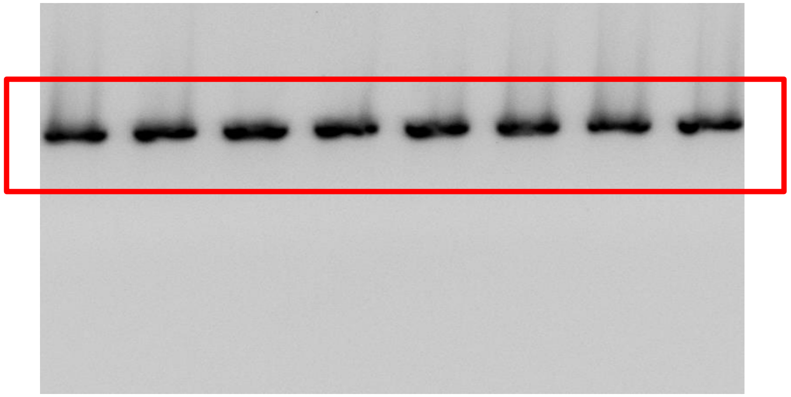

WCL IB:Flag

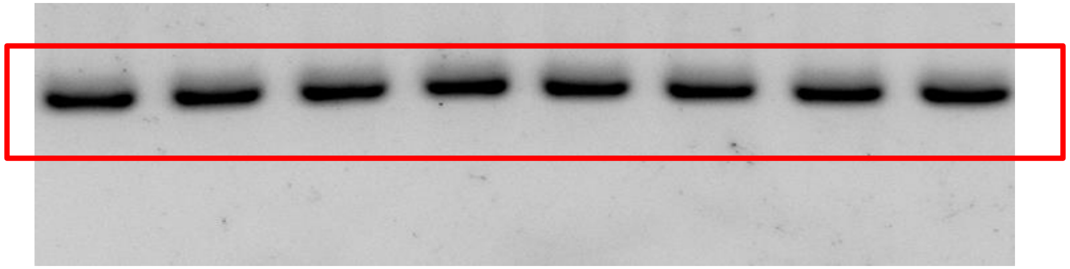

WCL IB:GAPDH

## Figure S5A

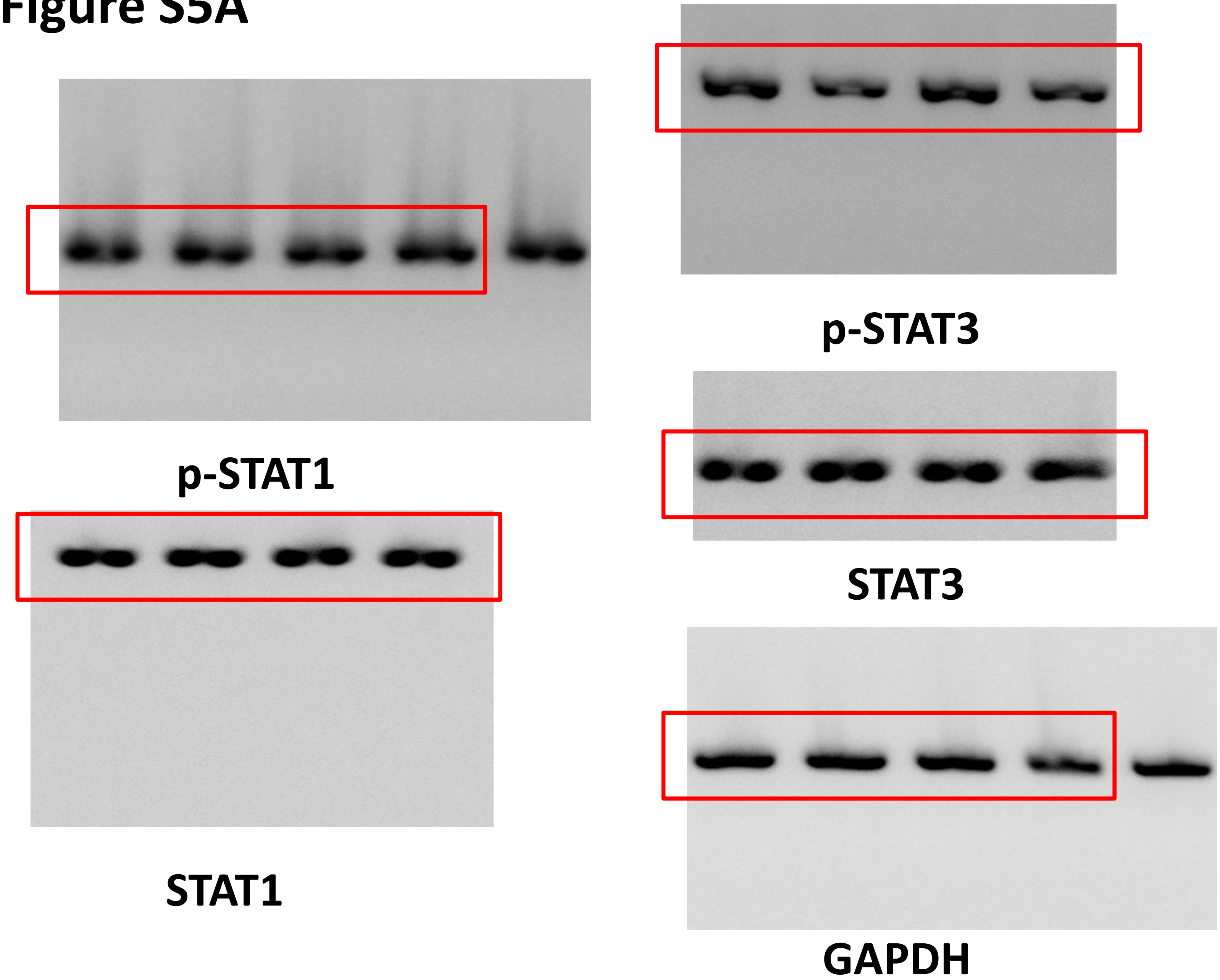

Figure S5F

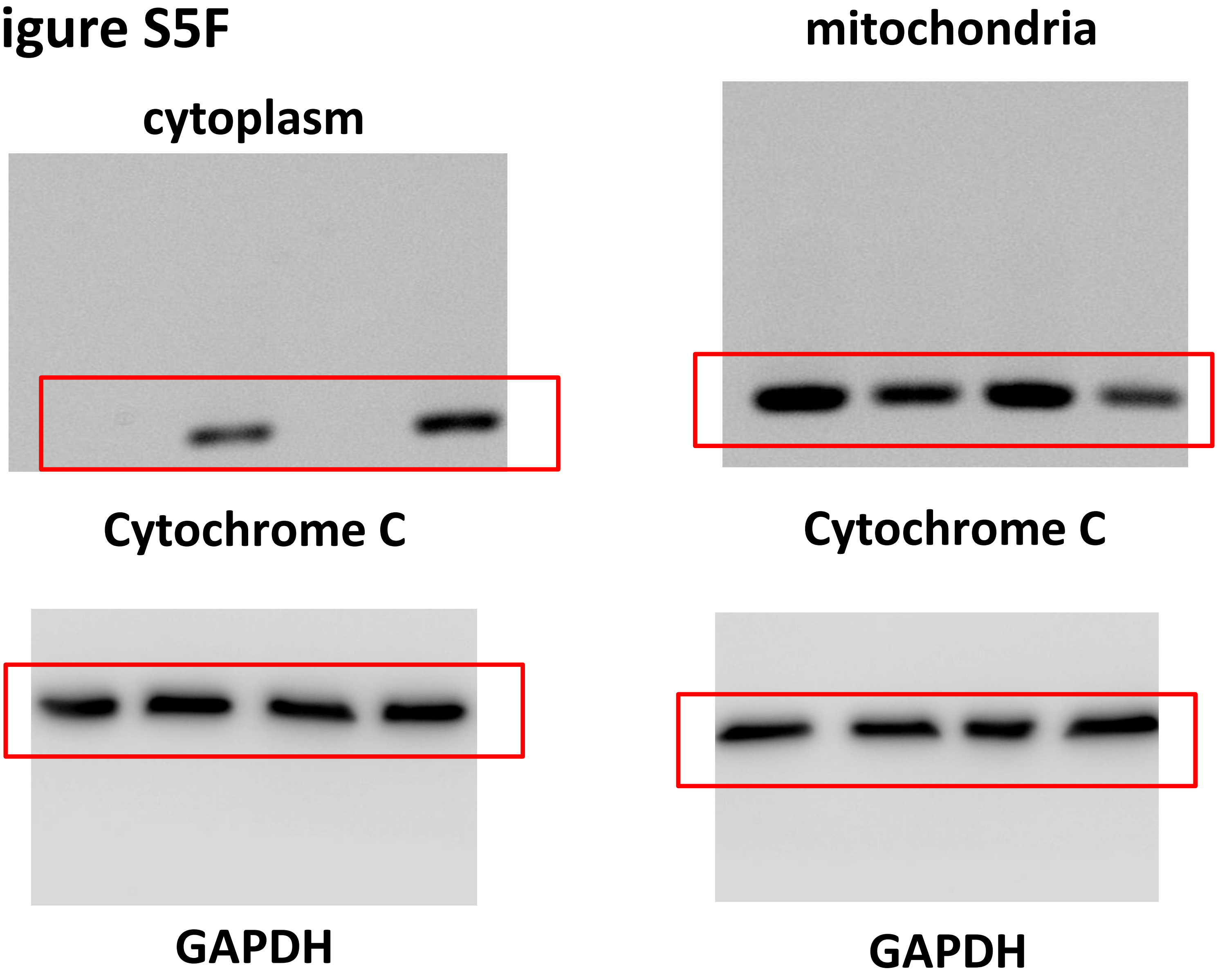
mitochondria
cytoplasm
Cytochrome C
Cytochrome C
GAPDH
GAPDH
